# Covalent adaptable networks using boronate linkages by incorporating TetraAzaADamantanes

**DOI:** 10.3389/fchem.2023.1148629

**Published:** 2023-02-23

**Authors:** Simon van Hurne, Marijn Kisters, Maarten M. J. Smulders

**Affiliations:** Laboratory of Organic Chemistry, Wageningen University, Wageningen, Netherlands

**Keywords:** dynamic covalent chemistry, covalent adaptable network (CAN), boronic acid, TetraAzaADamantane, polymer (re)processing

## Abstract

Boronic esters prepared by condensation of boronic acids and diols have been widely used as dynamic covalent bonds in the synthesis of both discrete assemblies and polymer networks. In this study we investigate the potential of a new dynamic-covalent motif, derived from TetraAzaADamantanes (TAADs), with their adamantane-like triol structure, in boronic ester-based covalent adaptable networks (CANs). The TetraAzaADamantane-boronic ester linkage has recently been reported as a more hydrolytically stable boronic ester variant, while still having a dynamic pH response: small-molecule studies found little exchange at neutral pH, while fast exchange occurred at pH 3.8. In this work, bi- and trifunctional TetraAzaADamantane linkers were synthesised and crosslinked with boronic acids to form rubber-like materials, with a Young’s modulus of 1.75 MPa. The dynamic nature of the TetraAzaADamantane networks was confirmed by stress relaxation experiments, revealing Arrhenius-like behaviour, with a corresponding activation energy of 142 ± 10 kJ/mol. Increasing the crosslinking density of the material from 10% to 33% resulted in reduced relaxation times, as is consistent with a higher degree of crosslinking within the dynamic networks. In contrast to the reported accelerating effect of acid addition to small-molecule TetraAzaADamantane complexes, within the polymer network the addition of acid increased relaxation times, suggesting unanticipated interactions between the acid and the polymer that cannot occur in the corresponding small-molecules analogues. The obtained boronate-TetraAzaADamantane materials were thermally stable up to 150°C. This thermal stability, in combination with the intrinsically dynamic bonds inside the polymer network, allowed these materials to be reprocessed and healed after damage by hot-pressing.

## Introduction

The poor recyclability of most modern plastics, in particular of thermoset materials, has created a focus on making more sustainable and better recyclable materials. This recyclability issue can be tackled by incorporating dynamic covalent bonds (Rowan et al., 2002) inside crosslinked polymer materials to create so-called covalent adaptable networks (CANs) (Kloxin and Bowman, 2013; Winne et al., 2019; Bowman et al., 2020; Zheng et al., 2021). While dynamic covalent bonds have proven very successful in the creation of dynamic combinatorial libraries (Hamieh et al., 2013; Mondal and Hirsch, 2015), discrete cage(-like) assemblies (Mastalerz, 2010), or covalent organic frameworks (Hu et al., 2020), their use in polymer networks has only more recently been acknowledged (Kloxin et al., 2010). Instead, polymer chemists previously focussed primarily on reversible, non-covalent interactions (notably hydrogen bonds) (Yang et al., 2015). However, by utilising dynamic covalent bonds, a more robust network can be created with crosslinks that can be made to exchange under specific conditions (e.g., by application of heat or light), thus allowing for reprocessing and recycling. The dynamicity of the introduced reversible crosslinks also means that the polymer material can undergo self-healing, which can extend the lifetime of the material (Kissounko et al., 2018; Zhang et al., 2018; Hayashi, 2020). Crucially, the use of dynamic bonds that are covalent in nature means that while these CANs can be recycled and/or demonstrate self-healing behaviour, at their operating temperature they still form a robust network–as a direct consequence of these covalent crosslinks–that impart mechanical strength to the material. Over the last few decades various dynamic bonds have been explored for use in CANs; these include the Diels-Alder reaction (Briou et al., 2021; Gaina et al., 2021), imines (Taynton et al., 2014; Schoustra et al., 2020), esters (Montarnal et al., 2011; Liu et al., 2020) and vinylogous urethanes (Denissen et al., 2015). Also, boronic acids (Bapat et al., 2020; Gosecki and Gosecka, 2022) have received an increasing amount of interest, due to their dynamic reversible condensation reaction with diols and general biocompatibility (Yang et al., 2005; Cambre and Sumerlin, 2011; Banach et al., 2021). Coupled with the affinity of boronic acids towards catechols (He et al., 2011) and sugars (Cai et al., 2017; Geethanjali et al., 2017), this has led to various applications to include boronic acids in medical applications, such as drugs (Plescia and Moitessier, 2020) and medical gels (Yesilyurt et al., 2016; Marco-Dufort and Tibbitt, 2019; Amaral et al., 2020), and biodegradable materials (Li et al., 2013; Zych et al., 2021). Boronic esters have also been used to form dynamic assemblies such as micelles (Zhao et al., 2014; Zhang et al., 2020a) or cage structures (Takata et al., 2020; Giraldi et al., 2021). Even a dynamically self-assembled nanoscale boronate ester ladder has been reported (Drogkaris and Northrop, 2020).

**FIGURE 1:**
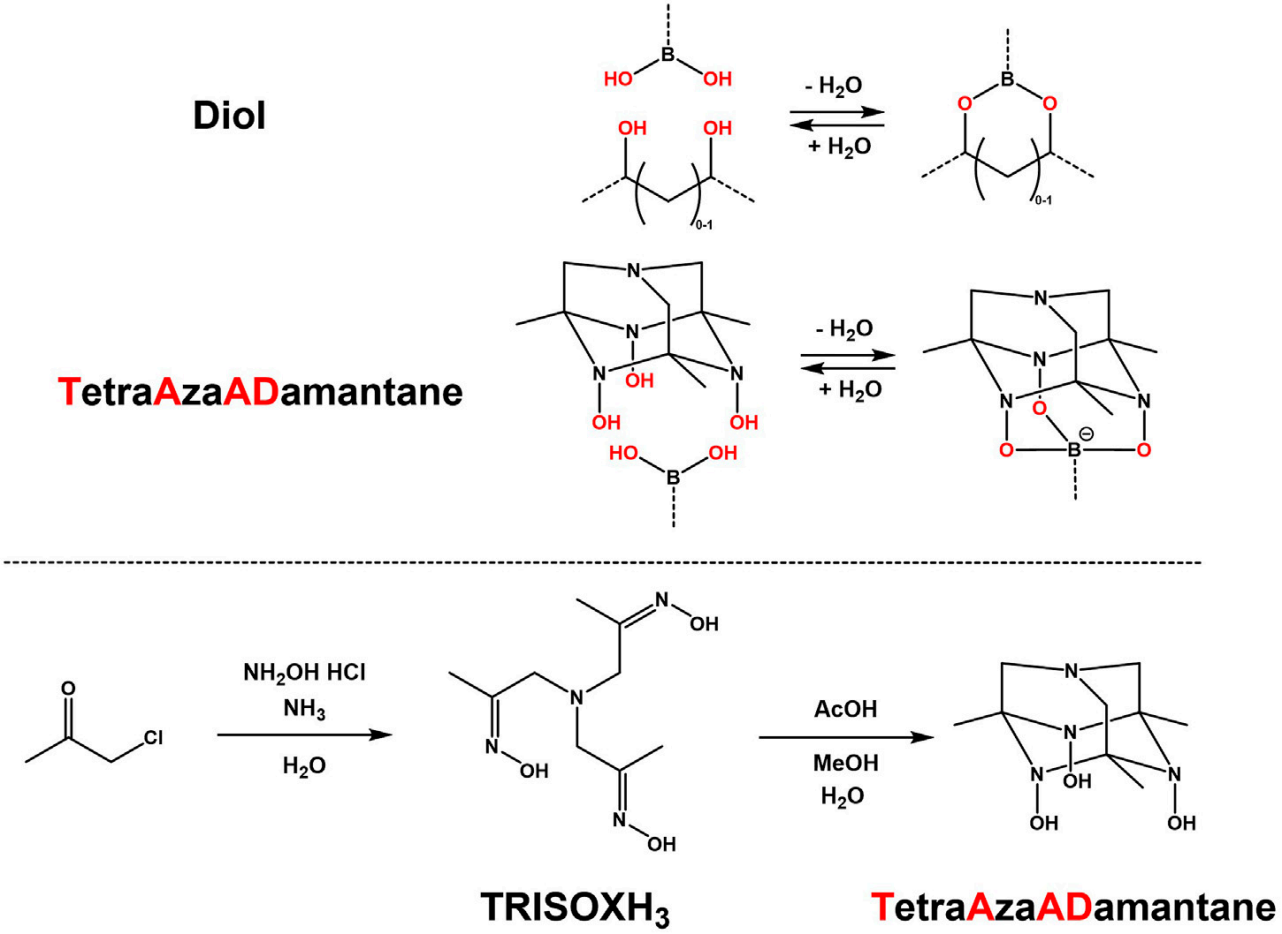
Comparison between (conventional) bidentate complexation of a boronic acid with a diol, and novel tridentate complexation by TetraAzaADamantane. Below, the synthesis pathway of tris-oxime TRISOXH_3_ and subsequent conversion into TetraAzaADamantane as reported (Semakin et al., 2009).

Typically, bidentate boronic acid linkages are used *via* reactions with diols or poly-ols, which usually result in the formation of soft (hydro)gels (Roberts et al., 2007; Wang et al., 2018; Perera and Ayres, 2020; Yang et al., 2021). However an interesting moiety (Semakin et al., 2009) was found to be able to consistently give rigid tridentate complexes of boronic acids, due to its well defined adamantane-like structure and conformation. This triol was named TetraAzaADamantane (TAAD) and is shown in Figure 1. TAAD can be obtained from the precursor tris-oxime named **TRISOXH**_**3**_ (Golovanov et al., 2018), which is prepared in a one-pot synthesis, after which an acid is used to catalyse the conversion into the final adamantane-like conformation (see Figure 1).

This TAAD moiety was then further investigated (Golovanov et al., 2015; Golovanov et al., 2018). It was found that the tridentate boronate complexation was stable at neutral to high pH with little dynamic exchange. However, around pH 3-4 dynamic exchange of the boronate groups was triggered, while at even lower pH complete disassembly of the boronate linkages was observed (Golovanov et al., 2018). Coupled with a quick and simple synthesis, and the more rigid multicyclic TAAD structure, we envisioned that this dynamic response makes the TAAD moiety an interesting candidate for use in CANs to create novel boronic acid-based materials.

**FIGURE 2:**
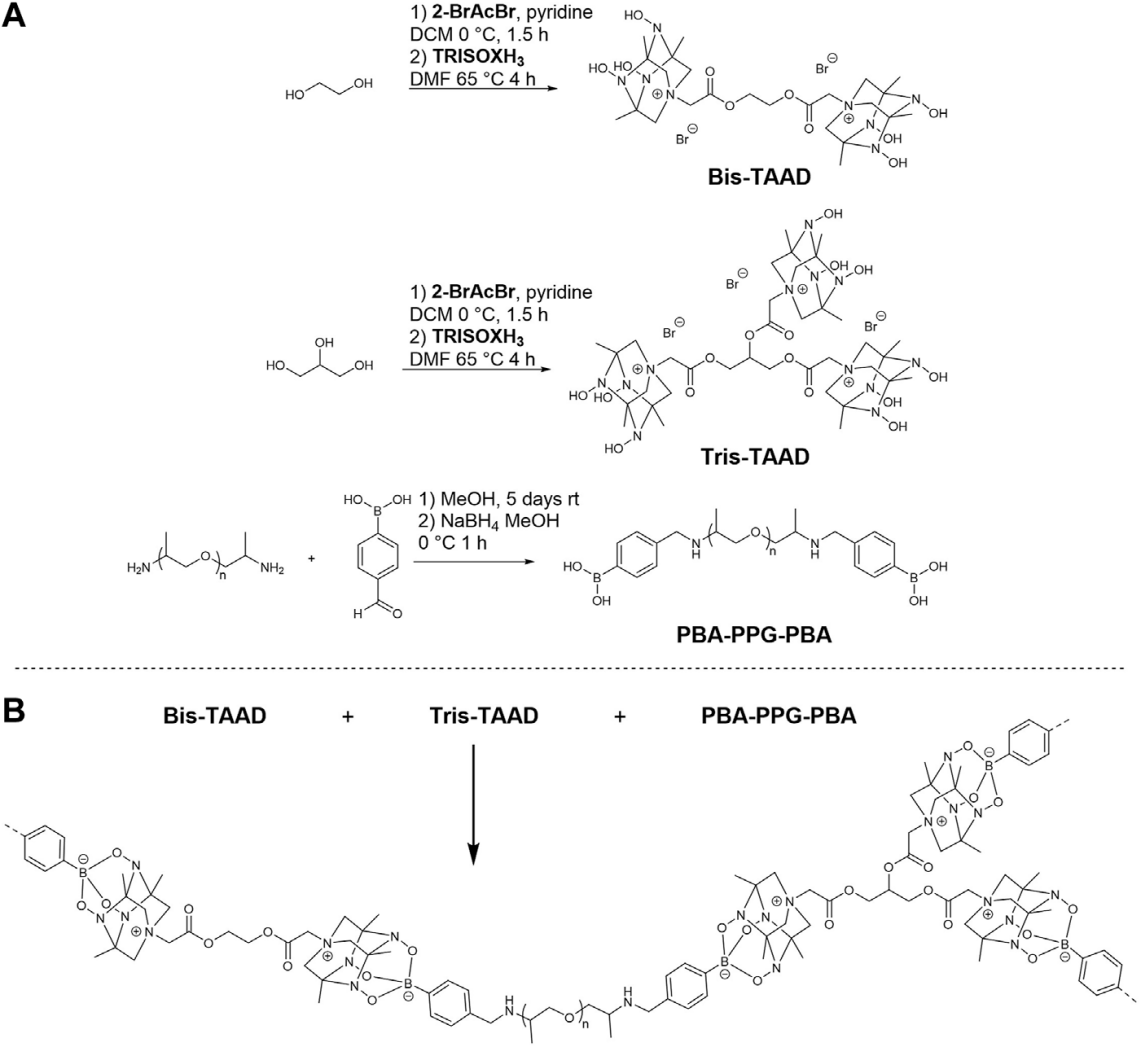
Synthesis and structure of the phenylboronic acid and TAAD linkers used in the boronate network **(A)**, and synthesis of the TAAD-based polymer networks **(B)**. For clarity, the counter anions have been omitted in the bottom figure.

In this work, we investigate the potential of the TAAD moiety in dynamic CANs. To this end, bifunctional boronic acid linkers and bi- and trifunctional TAAD linkers were designed and synthesised, as shown in Figure 2. These linkers were then reacted to form a crosslinked dynamic network. We found that the TAAD moieties can be successfully used for the formation of CANs. By using a small molecule network approach soft, rubber-like materials were prepared that were suitable for thermal reprocessing without the need for additional solvent and that could undergo stress relaxation.

## Results and discussion

To incorporate TetraAzaADamantane (TAAD) groups in a boronate ester network, different linker molecules were synthesised. Both a linear bifunctional boronic acid and TAAD linker were prepared, as well as a trifunctional TAAD linker to enable crosslinking. For the linear boronic acid crosslinker it was chosen to react bis-amine terminated PPG_2000_ with 4-formylphenylboronic acid and subsequently reducing the formed imine bond (Bao et al., 2018). For the bi- and trifunctional TAAD linkers **Bis-TAAD** and **Tris-TAAD**, ethylene glycol and glycerol, respectively, were reacted with bromoacetyl bromide. After a short work up, **TRISOXH**_**3**_ was attached *via* nitrogen quaternisation and converted to the desired TAAD-containing building blocks (see Supplementary Material for full details of the synthetic procedures and characterisation).

To show the reversibility and exchange of the boronate-TAAD bond a small series of exchange studies was performed using differently substituted boronic acids, expanding on the existing exchange experiment involving only 4-bromophenylboronic acid (Golovanov et al., 2018). A model TAAD molecule coupled to a phenylboronic acid, resembling the boronate-TAAD linkages used in the materials, was synthesised and exchanged with three different *para*-substituted phenylboronic acids (see Supplementary Material). This exchange was followed using ^1^H-NMR spectroscopy, where both the disappearance of the signals corresponding to starting boronate-TAAD and the appearance of signals of the exchanged product were monitored in time. All showed slow exchange over the course of 3 days, resulting in only ∼20% conversion. Subsequently, the pH was adjusted to 3.8 to enter the dynamic pH regime and the exchange reaction was followed for 5 h. All three substituted phenylboronic acids showed an increase in exchange rate as expected (Supplementary Figures S35–S38), reaching 40%–60% conversion after only 5 h. This shows that the boronate-TAAD linkages that are to be incorporated in the network can undergo exchange reactions.

Next, polymer materials were prepared by dissolving the **Bis-TAAD**, **Tris-TAAD**, and **PBA-PPG-PBA** in separate methanol stock solutions. These stock solutions were then combined using a stoichiometry TAAD:boronic acid of 1:1, and initially with 10% of the crosslinker **Tris-TAAD**. The resulting mixture was vortexed, then cast in a silicone mould and left to dry in the fume hood for 2 days. Using this method, a soft rubber-like material was obtained that was slightly yellowish in colour. Initial characterisation by thermal gravimetric analysis (TGA, Supplementary Figure S65) showed that the material was thermally stable up to 153°C with only 5% weight loss. This thermal stability suggests that (re)processing of the material at elevated temperatures (e.g., by hot-pressing) can be explored up to temperatures of approximately 150°C. Below, we will further discuss the thermal (re)processing by hot-pressing.

**FIGURE 3:**
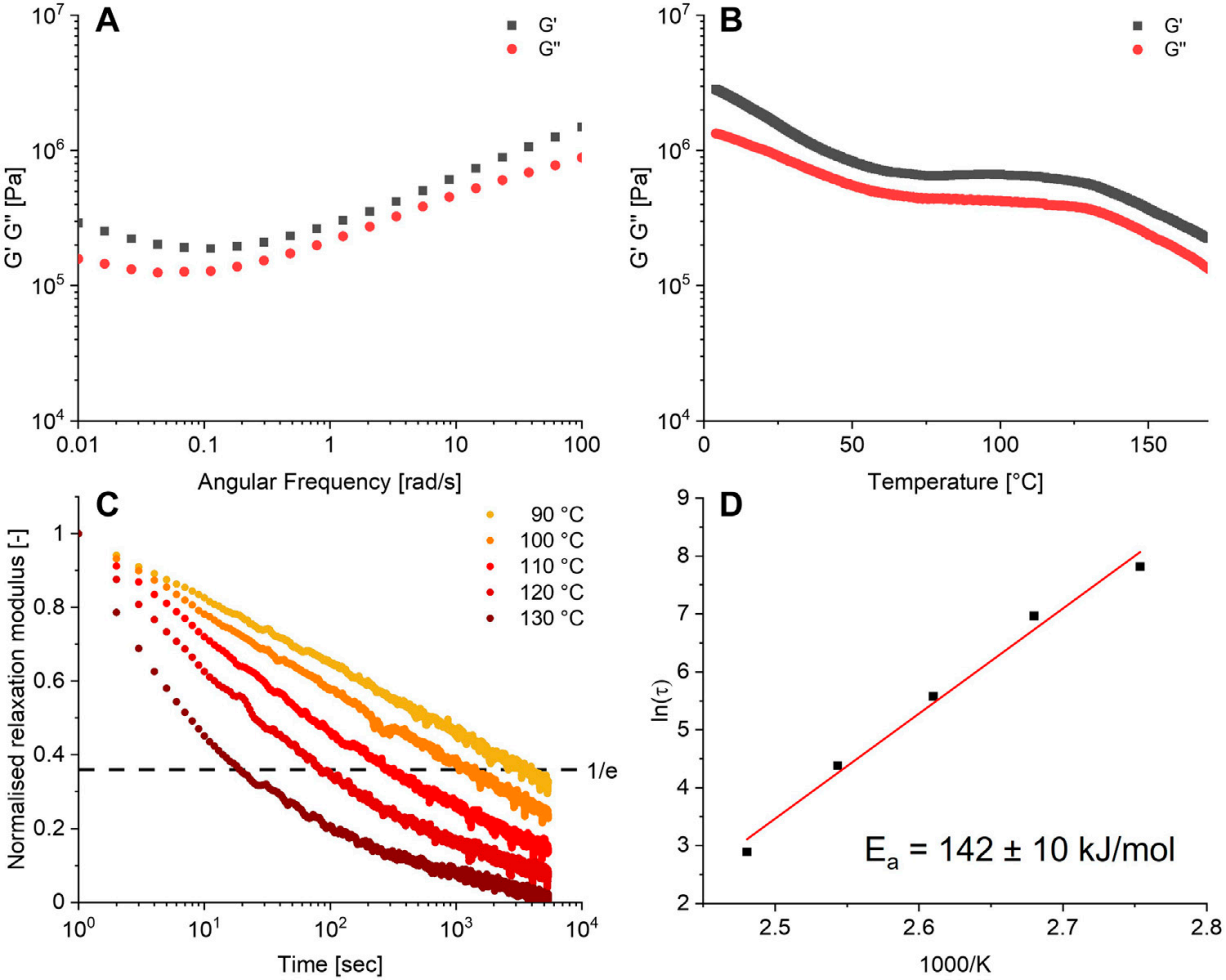
**(A)** Frequency sweep at 0.5% strain, 25°C. **(B)** Temperature sweep at 0.5% strain, 6.28 rad/s. **(C)** Relaxation series at 0.5% strain, 90°C–130°C. **(D)** Arrhenius plot obtained from the relaxation data. All data were obtained from the 10% crosslinked material.

The obtained polymer was then further characterised by rheometry. A frequency sweep of the material, as shown in Figure 3A, yielded a *storage modulus (G′)* and *loss modulus (G″)* between 0.1 and 1 MPa over the frequency domain of 0.01–100 rad/s. Extensional DMA showed that the material had a Young’s modulus of 1.75 MPa, corresponding to a soft rubber. The temperature sweep from 4°C to 170°C (Figure 3B) of the formed network showed a typical rubbery plateau at a *G*_P_ of 1 MPa between 70°C and 125°C suggesting an entangled or crosslinked network, after which the material started to flow. The glassy state was not observed, but could likely be observed at lower temperatures.

A defining feature of CANs is their ability to undergo stress relaxation by dynamic bond exchange. The time for the material to relax imposed stress generally shortens with an increase in temperature, since the dynamic bond exchange becomes faster. This allows faster network rearrangement and thus faster stress relaxation. The Arrhenius equation can be applied to the dependence of the relaxation time (*τ*) with temperature, which will yield the activation energy *E*_a_ of relaxation process due to bond exchange. To this end, the relaxation behaviour of the boronate-TAAD network was investigated in the temperature range of 90°C–130°C. The observed relaxation time *τ* decreased from 2,478 s at 90°C to 18 s at 130°C, as can be seen in Figure 3C. From the corresponding Arrhenius plot (Figure 3D) for the boronate-TAAD exchange reaction an activation energy of 142 ± 10 kJ/mol could be determined, which is quite high compared to previously reported materials based on boronic esters (Cromwell et al., 2015; Röttger et al., 2017; Zhang et al., 2020b; Kang and Kalow, 2022) and more in the upper range of diethanolamine esters (Zhang et al., 2020b; Gosecki and Gosecka, 2022). However, it is important to note that most reported boronic ester materials were prepared as a (hydro)gel, which can heavily influence the determined activation energy. Remarkably, in contrast to the pH range of 3-4 necessary for dynamic exchange in the reactions (Golovanov et al., 2018), within this dynamic network, (stress) relaxation already manifested itself at neutral pH.

**FIGURE 4:**
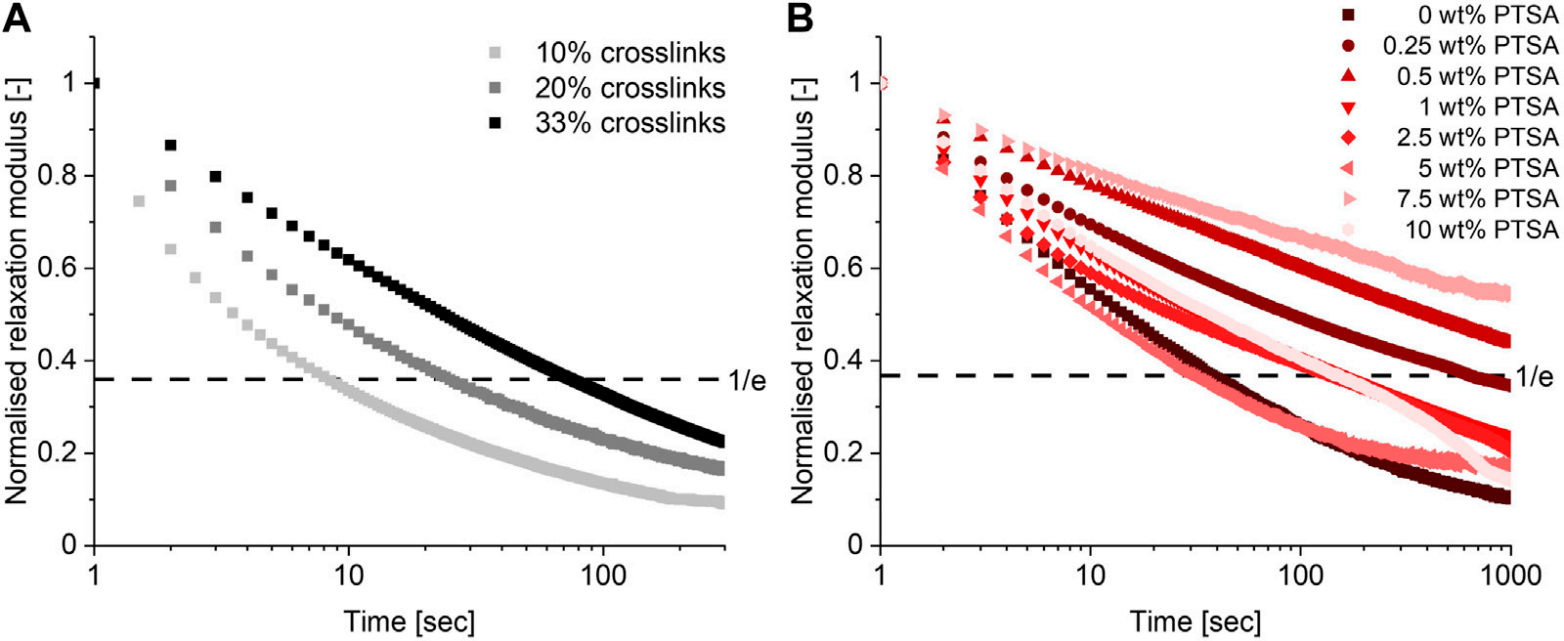
Relaxation curves (at 0.1% strain, 25°C) of materials with varying crosslink densities **(A)** and the effect of acid addition (at 0.1% strain, 25°C) on the material properties **(B)**.

Having established that a TAAD-based CAN could be prepared with a 10% crosslinker content, the effect of the crosslink density on the CAN’s material properties was investigated. Additional CANs with 20% and 33% crosslinks were prepared and compared. Frequency sweeps (Supplementary Figures S39, S42) did not show a significant change to the moduli as *G′* and *G″* of the different materials were in the range of 0.1–1 MPa. Temperature sweeps (Supplementary Figures S41, S44) did show that increasing the crosslinking density shifted the onset of the rubbery plateau from around 50 °C to around 120°C–130 °C, which can be explained by considering the higher crosslink density. Next to this, the relaxation time (*τ*) of the material increased by one order of magnitude with increasing crosslink density from 10% to 33%, as can be seen in Figure 4A. The added crosslinks reduce network mobility, thus slowing the material relaxation process (Hajj et al., 2020). This shows that it is possible to tune material properties by varying the crosslink density of these networks.

Since the boronate-TAAD network features pH-dependent bonds (Golovanov et al., 2018), also the effect of acid addition to the material was investigated. Previously, researchers showed that addition of acid can accelerate the dynamic bond exchange processes inside a CAN (Denissen et al., 2017; Self et al., 2018; Spiesschaert et al., 2020). In these reports, the p*K*_a_ of the added acid was found to correlate with the shortening of the material relaxation time. Combined with the reported findings (Golovanov et al., 2018) of the different pH regimes of the boronate-TAAD linkages, we anticipated that addition of acid in the material would have an effect by reducing the relaxation time of the material, *via* transesterification catalysis.

To investigate the effect of acid on this boronate-TAAD network a series of materials was prepared by mixing in different weight percentages of *p*-toluenesulfonic acid (PTSA), ranging from 0.25 wt% to 10 wt%. The relaxation behaviour of these materials was then measured and evaluated, as shown in Figure 4B. The observed relaxation behaviour was more complex than initially anticipated: *i.e.*, the addition of acid did not simply lead to faster relaxation. In contrast, at low PTSA content (0.25–0.5 wt%) an increase in the relaxation time of the material compared to the material without PTSA was observed. These materials displayed a relaxation time that is about one order of magnitude longer than the original acid-free material. At moderate PTSA content (1–5 wt%) the relaxation time is still generally longer than without PTSA, but it started to decrease until at 5 wt% PTSA the material had a relaxation time similar to the control without PTSA. At high PTSA content (5–10 wt%) again an increase in relaxation time was observed. While the precise underlying mechanism of these trends remains unclear, one possible explanation might be that the PTSA is partially excluded from the network due to unfavourable interactions between the apolar aromatic ring of the acid and the polar building blocks, thus reducing its effectiveness as a catalyst while possibly introducing crystalline regions in the matrix. Another possible explanation could be an interaction with the secondary amines of the PBA-PPG-PBA linker. PTSA could protonate the secondary amines of this linker, resulting in the formation of charged nitrogen species similar to polyelectrolyte complexes, which often become stiff and brittle upon drying (Fares et al., 2019; Krishna et al., 2022). This unexpected acid response in the material relaxation shows that, although the characteristics of this boronate-TAAD network can be tuned through acid addition, predicting the resulting material behaviour might prove complex. Fortunately, since the control material was already quite dynamic even at neutral pH there was little need for additional acid catalysis. More generally, it also serves as a reminder that translating molecular processes (i.e., acid-catalysed transesterification) to the macroscopic material level, is not always straightforwardly possible, as other macroscopic phenomena, such as crystallisation, can affect the process.

Another interesting observation could be seen in the temperature sweeps of these materials, where higher PTSA weight percentages resulted in the material melting at high temperatures, as shown by the loss factor steeply increasing (Supplementary Figure S64) This was not observed for the control material without PTSA.

**FIGURE 5:**
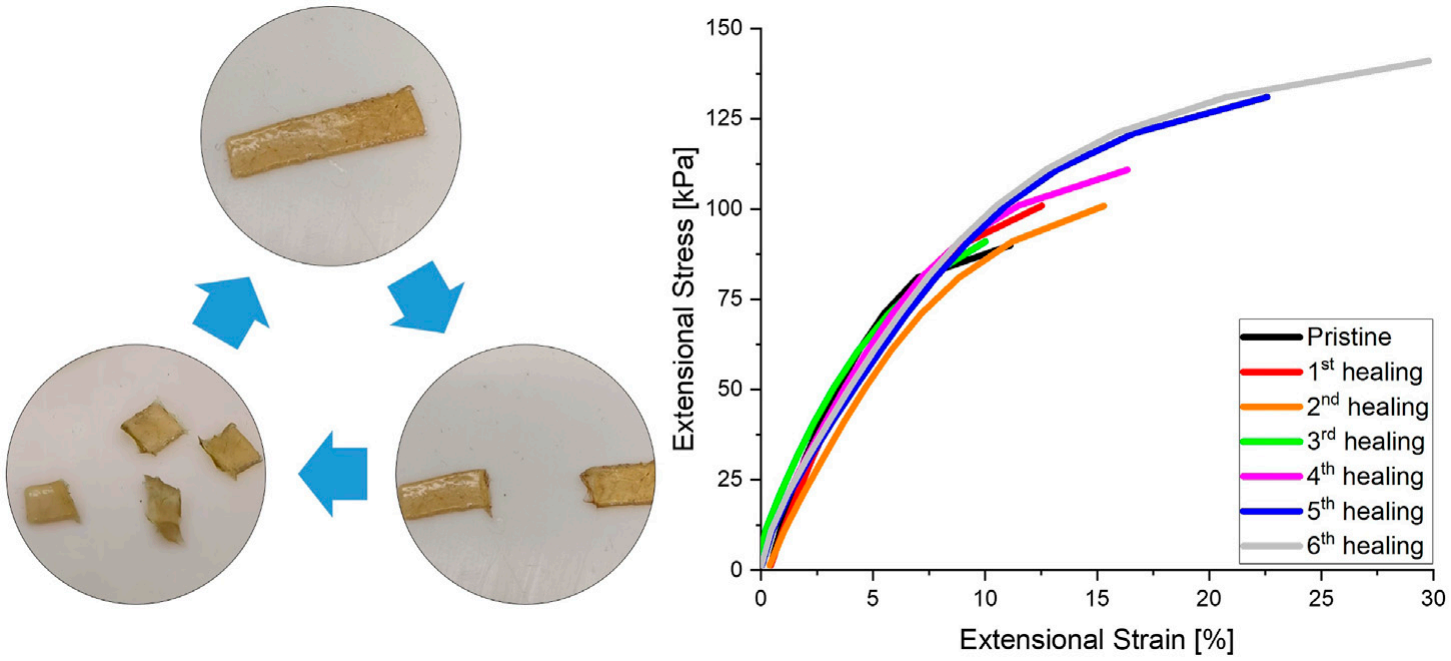
Healing cycles performed by extensional DMA testing. Reprocessing was done by hot-pressing for 1 h at 80°C in a Teflon mould (10 × 5 × 1 mm). DMA was performed by linearly increasing the extensional stress till material breakage. After breakage the sample was cut into small pieces before reprocessing.

The self-healing behaviour and recyclability of the material, as might be enabled by the dynamic bond exchange within the network, was also investigated using extensional DMA. The strain at which material breakage occurred was measured by linearly increasing the extensional stress of a CAN with 10% crosslinker. After breakage the material was cut in fine pieces and hot-pressed for 1 h at 80°C to allow the material to heal. As can be seen in Figure 5, the material is able to completely heal after multiple reprocessing cycles. The strengthening of the last few healing cycles seems to suggest that the network configuration is optimised during the applied thermal treatment.

## Conclusion

In this study we synthesised a novel covalent adaptable network incorporating dynamic boronate-TAAD linkages using a small molecule network approach, resulting in a soft, rubber-like material. The material was thermally stable up to 150°C as determined by TGA, thus allowing for hot-press moulding. In contrast to the behaviour of previously reported small-molecule boronate-TAAD complexes that could only undergo (fast) exchange under acidic conditions (pH 3.8), the corresponding CANs could already undergo (stress) relaxation under neutral conditions. Surprisingly, the addition of acid did not have a further accelerating effect on the exchange. The boronate-TAAD material showed complete self-healing after multiple thermal reprocessing cycles and Arrhenius analysis gave a relatively high activation energy of 142 ± 10 kJ/mol for the crosslink relaxation process compared with regular boronic esters.

This first investigation into boronate-TAAD networks showed that the novel boronate-TAAD linkages can indeed be used to develop CANs. Coupled to the relatively simple synthesis of the TAAD moiety, this unique boronate-TAAD linkage offers an interesting alternative to the more commonly used boronic esters.

## Data Availability

The raw data supporting the conclusions of this article will be made available by the authors, without undue reservation.
