# Supplementary material for: Covalent adaptable networks using boronate linkages by incorporating TetraAzaADamantanes
Source: Front Chem. 2023 Feb 23;11:1148629. doi: 10.3389/fchem.2023.1148629 (PMC9995436; doi:10.3389/fchem.2023.1148629)
Supplement: Supplementary file 1 [file DataSheet1.pdf]

Supplementary Material

Contents

1 Methods 2
1.1 NMR 2
1.2 IR 2
1.3 Rheology & DMA 2
1.4 Hot-press 2
1.5 TGA 2
1.6 Mass spectrometry 2
1.7 Synthesis of TRISOXH_3 as reported by (Golovanov et al., 2018) 3
1.8 Synthesis of bis(bromoacetoxy)ethane 3
1.9 Synthesis of tris(bromoacetoxy)propane 4
1.10 Synthesis of bis(TAADacetoxy)ethane [bis-TAAD] 4
1.11 Synthesis of tris(TAADacetoxy)propane [tris-TAAD] 5
1.12 Synthesis of bisboronic acid crosslinker PBA-PPG-PBA adapted from (Bao et al., 2018) 6
1.13 Synthesis of N-methylacetate-O-phenylboronate-TAAD adapted from (Golovanov et al., 2018) 7
1.14 Preparation of boronic acid-TAAD networks 7
1.15 Self-healing properties 8
1.16 Exchange equilibrium study 8
2 NMR data 9
3 IR data 36
4 Mass spectrometry data 40
5 Exchange equilibrium data 41
6 Rheology data 45
7 TGA data 60
8 References 60

1 Methods

Acetic acid, ammonium hydroxide ge 25% solution, chloroacetone, glycerol, hydroxylamine hydrochloride, magnesium sulfate and p-tolylboronic acid were bought from Fisher Scientific B.V. Ethylene glycol, poly(propylene glycol) bis(2-aminopropyl ether) Mn 2000, Li_2CO_3, methyl bromoacetate, phenyl boronic acid, pyridine and sodium borohydride were bought from Merck Life Sciene N.V. Bromoacetyl bromide and HCl (37% in water) were obtained from Acros Organics. Anhydrous Na_2CO_3 was bought from Alfa Aesar. 4-formylphenylboronic acid was bought from abcr.

Pur-A-Lyzer mega dialysis kit with a volume of 20 ml and a cut-off of 1 kDA was obtained from Merck Life Science N.V.

Common laboratory solvents were used from various suppliers. All chemicals were used without further purification.

1.1 NMR

Spectra were recorded on a 400 MHz Bruker NMR (101 MHz ^{13}C).

1.2 IR

Bruker TENSOR 27 Platinum FTIR spectrometer in Attenuated Total Reflection (ATR) mode, controlled by Bruker's OPUS software. Spectra were recorded from 600 to 4000 cm^{-1} with a resolution of 4 cm^{-1} and were averaged over 60 scans.

1.3 Rheology & DMA

Rheology experiments were done on an Anton Paar MCR 501 and a 702e space using 10 mm parallel plate geometries. The 501 used a N_2 heat mantle, while the 702e space used a N_2 driven piezzo oven for heating.

DMA was performed on the 702e space using the linear drive setup at ambient temperature with extensional clamps.

1.4 Hot-press

Samples were hot-pressed in a Specac Atlas Series Heated Platens with a WEST 6100+ Temperature Controller Unit with water cooling in a Teflon mold between Teflon sheets. Samples were hot-pressed for 1 hour at 80 °C.

1.5 TGA

TGA was measured using a Perkin Elmer Simultaneous Thermal Analyzer (STA) 6000. Samples were measured over the temperature range of 30 to 900 °C with a temperature increase of 10 °C/min.

1.6 Mass spectrometry

Mass spectrometry data were acquired using MS-ESI Thermo scientific Exactive in positive mode. The compounds were ionized without fragmentation at 0 kV spray voltage and with a capillary temperature of 150 °C.

1.7 Synthesis of $TRISOXH_3$ as reported by (Golovanov et al., 2018)

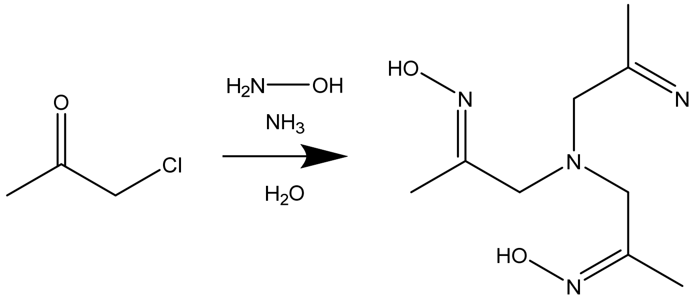

Supplementary Scheme S1. Synthesis of $TRISOXH_3$

In a 250 ml roundbottom flask 10.2 g (0.15 mol) of hydroxylamine hydrochloride was dissolved in 60 ml water. 60 ml of a 25% ammonium hydroxide solution in water was added. The flask was put in a water bath to provide passive cooling. Then 11.8 ml (0.15 mol) chloroacetone was added via syringe. The reaction was stirred for 1 hour, afterwhich the white precipitate was filtered off and washed with plenty of water and finally with diethyl ether. The white powder was then dried overnight at 50 °C in a vacuum oven. This resulted in a fine white powder (3.38 g; 29.7%).

^1H NMR (400 MHz, DMSO) δ 10.57 (s, 3H), 2.90 (s, 6H), 1.74 (s, 9H).

1.8 Synthesis of bis(bromoacetoxy)ethane

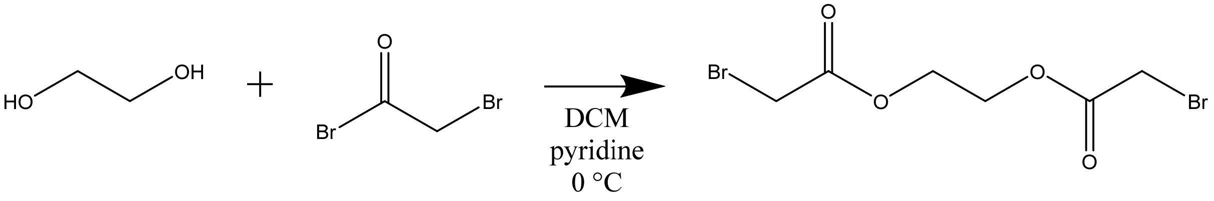

Supplementary Scheme S2. Synthesis of bis(bromoacetoxy)ethane

0.85 ml (9.7 mmol) bromoacetyl bromide was dissolved in 10 ml DCM in a 250 ml round bottom flask and cooled down to 0°C. To this a mixture of 0.25g (4.0 mmol) ethylene glycol and 0.71 ml (8.8 mmol) pyridine in 2 ml DCM was slowly added over 30 minutes at 0°C. After complete addition, the ice bath was removed and the reaction mixture was stirred for 1.5h at room temperature. To the reaction 3 ml 6M HCl was added followed by 3 ml DCM. The two layers were separated and the aqueous layer was extracted with DCM. The organic layers were combined and extracted with 25 ml water, 3x 25 ml Na_2CO_3 (aq), and 40 ml brine. The organic layer was dried over $MgSO_4$ and concentrated under reduced pressure to obtain a pale yellow liquid. Yield: 1.13g (90%).

^1H-NMR (400 MHz, DMSO): 4.35 (s, 4H, CH_2O), 4.16 (s, 4H, CH_2Br).

^{13}C-NMR (100 MHz, DMSO): 167.60 (CH_2CO_2), 63.76 (CH_2O), 27.39 (CH_2Br).

1.9 Synthesis of tris(bromoacetoxy)propane

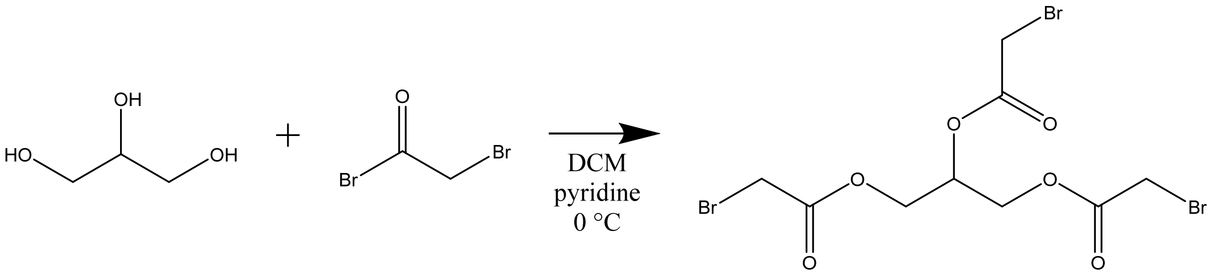

Supplementary Scheme S3. Synthesis of tris(bromoacetoxy)propane

1,2,3-tris(bromoacetoxy) propane 5a was obtained from 0.37 g (4.1 mmol) glycerol 2a, 3.01 g(14.9 mmol) bromoacetyl bromide and 0.98 g (12.4 mmol) pyridine with the same synthesis as 1,2-bis(bromoacetoxy)ethane. Yellowish oil was obtained, yield 1.56g (85%).

^{1}H-NMR (400 MHz, DMSO): 5.30 (m, 1H, CHO) 4.36 (dd, 4H, CH_2O), 4.17 (s, 4H, CH_2Br).

^{13}C-NMR (101 MHz, DMSO): 167.44 (CH_2CO_2), 70.88 (CHO), 63.79 (CH2O), 27.30 (CH_2Br).

1.10 Synthesis of bis(TAADacetoxy)ethane [bis-TAAD]

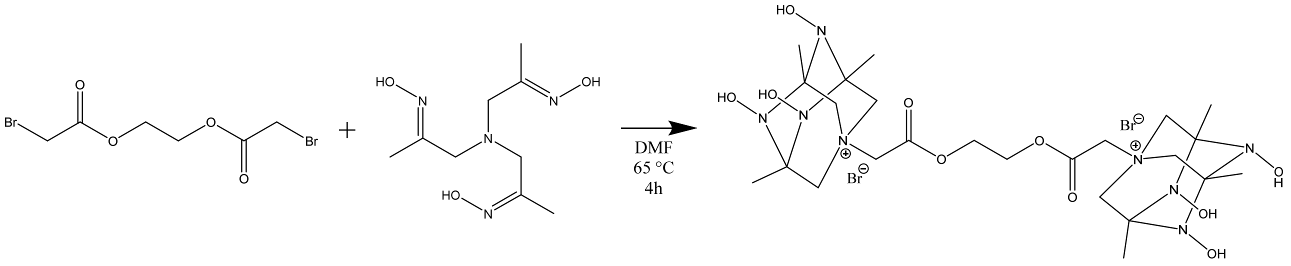

Supplementary Scheme S4. Synthesis of bis(TAADacetoxy)ethane

A mixture of 0.42 g (1.8 mmol) $TRISOXH_3$ and 0.25 g (0.83 mmol) 1,2-bis(Bromoacetoxy)ethane in 5 ml DMF in a 25 ml round bottom flask was stirred at 65°C. After 4h the reaction mixture was gently added to 200 ml acetone, a white precipitate formed and acetone was decanted off. Next, the precipitate was washed 2x with 200 ml acetone, whereafter the precipitate was filtered off and the white solid was dried in a vacuum oven at 50 °C. Yield: 0.49 g (78%).

^{1}H-NMR (400 MHz, DMSO): 8.80 (s, br, 6H, O**H**), 4.81 (s, 4H, $CO_2C\mathbf{H_2}N$), 4.44 (s, 4H, $C\mathbf{H_2}O$), 3.72 (s, br, 12H, $NC\mathbf{H_2}$), 1.24 (s, 18H, $C\mathbf{H_3}$).

^{13}C-NMR (101 MHz, DMSO): 164.52 (CH_2**C**O_2), 75.87 (br, **C**N), 63.62 (**C**H_2O), 61.60 (CO_2**C**H_2N), 56.87 (br, N**C**H_2), 21.09 (**C**H_3).

ESI-MS (m/z): $[M]^{2+}$ found, 302.16.

1.11 Synthesis of tris(TAADacetoxy)propane [tris-TAAD]

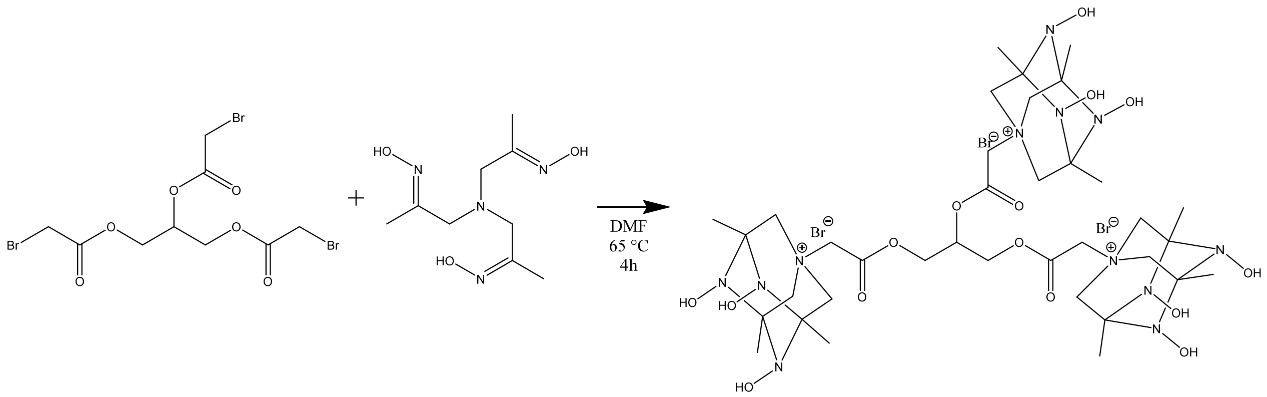

Supplementary Scheme S5. Synthesis of tris(TAADacetoxy)propane

To obtain 1,2,3-tris(TAADacetoxy)propane 0.68 g (1.5 mmol) 1,2,3-tris(bromoacetoxy)propane and 1.1 g (4.9 mmol) TRISOXH3 were dissolved in 20 ml DMF and the same method as 1,2-bis(TAADacetoxy)ethane was used. A white solid was obtained yield 1.5 g (90%).

^{1}H-NMR (400 MHz, DMSO): 8.49 (s, br, 9H, O**H**), 5.46 (m, 1H, C**H**O), 4.95 (d, 4H, CO_2C**H₂**N), 4.76 (d, 2H, CO_2C**H₂**N), 4.52 (d, 4H, C**H₂**O), 3.74 (s, br, 18H, NC**H₂**), 1.26 (s, 27H, C**H₃**).

^{13}C-NMR (101 MHz, DMSO): 164.36 (CH_2**C**O_2), 164.09 (CH_2**C**O_2), 75.47 (br, **C**N), 70.34 (**C**HO), 63.48 (**C**H_2O), 61.65 (CO_2**C**H_2N), 21.10 (**C**H_3).

ESI-MS (m/z): $[M]^{3+}$ found, 301.82.

1.12 Synthesis of bisboronic acid crosslinker PBA-PPG-PBA adapted from (Bao et al., 2018)

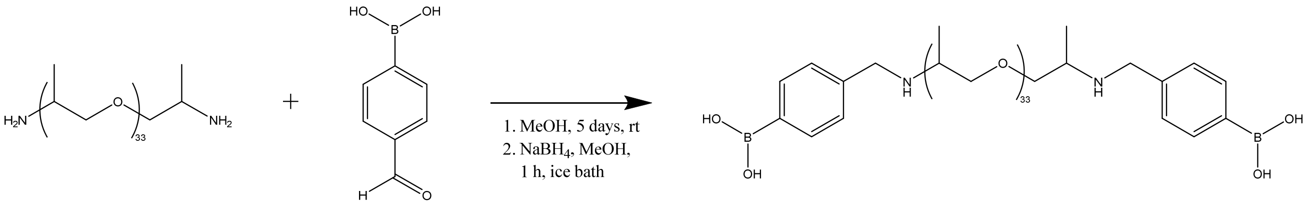

Supplementary Scheme S6. Synthesis of bisboronic acid crosslinker PBA-PPG-PBA

3.0 ml (1.5 mmol) Poly(propylene glycol) bis(2-aminopropyl ether) Mn 2000 (n = 33) and 1.37 g (4.1 mmol) 4-formylphenylboronic acid were dissolved in 10 ml methanol under nitrogen and stirred for 5 days. The solution was then cooled in an ice batch. Then 0.2386 g (5.1 mmol) $NaBH_4$ was added slowly. The solution was stirred for 1 hour at room temperature, after which it was dialysed against methanol for 2 days. After evaporation of the solvent a yellowish sticky plaque was obtained (1.44 g; 42.4%)

^{1}H NMR (400 MHz, MeOD) δ 7.47 (d, J = 7.4 Hz, 4H), 7.14 (d, J = 7.7 Hz, 4H), 3.83 (dq, J = 24.3, 12.4, 11.9 Hz, 3H), 3.59 – 3.22 (m, 106H), 1.04 (dd, J = 6.5, 3.1 Hz, 105H).

^{13}C NMR (101 MHz, MeOD) δ 208.93, 163.45, 133.51, 133.18, 132.93, 132.45, 129.58, 128.21, 126.92, 126.60, 125.43, 75.54, 75.50, 75.42, 75.40, 75.31, 75.28, 75.17, 75.15, 75.08, 75.03, 74.98, 74.95, 73.30, 72.99, 72.93, 72.72, 72.69, 72.62, 72.57, 66.52, 64.27, 56.29, 56.08, 55.86, 52.30, 52.03, 51.89, 50.05, 49.96, 49.85, 48.49, 48.28, 48.06, 47.85, 47.64, 47.42, 47.21, 47.00, 46.44, 39.10, 35.57, 30.29, 28.78, 28.58, 28.39, 17.38, 17.16, 17.02, 16.35, 16.30, 16.25, 16.01, 15.84, 15.77, 14.53.

1.13 Synthesis of N-methylacetate-O-phenylboronate-TAAD adapted from (Golovanov et al., 2018)

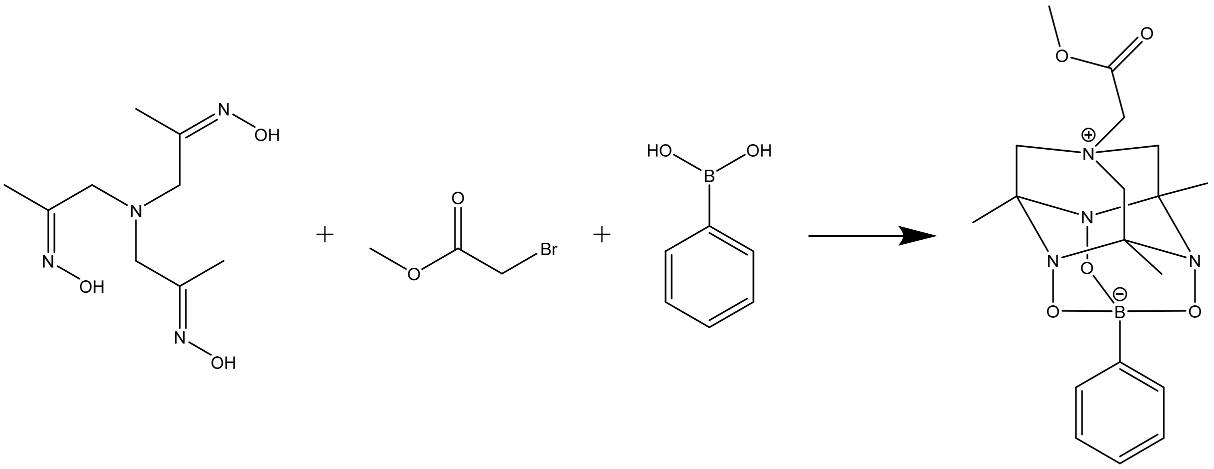

Supplementary Scheme S7. Synthesis of N-methylacetate-O-phenylboronate-TAAD

115 mg (0.5 mmol) $TRISOXH_3$, 61 mg (0.5 mmol) phenylboronic acid and 76.5 mg (0.5 mmol) methyl bromoacetate were dissolved in 1 ml ethanol and stirred for 20 hours. Then 18.5 mg (0.25 mmol) Li_2CO_3 was added. The reaction mixture was stirred for another 4 hours. Afterwards the solvent was evaporated and the solids were triturated diethyl ether : methanol (10 : 1) before drying in a vacuum oven overnight at 50 °C. a white solid was obtained (173 mg, 66%)

^{1}H NMR (400 MHz, D2O) δ 7.49 (m, 3H), 7.30 (m, 4H), 3.97 (s, 6H), 3.90 (t, J = 2.5 Hz, 3H), 1.80 (t, J = 2.5 Hz, 9H), 1.66 (t, J = 2.5 Hz, 2H).

^{13}C NMR (101 MHz, MeOD) δ 164.45, 131.02, 130.91, 130.87, 127.81, 127.30, 127.24, 127.21, 127.13, 127.07, 73.28, 72.91, 72.86, 65.83, 64.67, 64.01, 59.65, 57.31, 48.79, 48.61, 48.39, 48.18, 47.96, 47.75, 47.53, 47.32, 20.50, 20.46, 19.98, 16.93, 14.16.

1.14 Preparation of boronic acid-TAAD networks

Here the method of preparing a general 10% crosslinked boronic acid-TAAD network is described. The 20% and 33% crosslinked networks were prepared similarly, but with different ratios of the TAAD linkers. The experiments with the PTSA additions were performed by preparing 33% crosslinked networks and adding a certain wt% of PTSA.

To make the networks 3 solutions were prepared, which were then combined and cast in a mold. First 41.3 mg (0.045 mmol) Bis-TAAD was dissolved in 0.1 methanol. Secondly, 6.8 mg (0.005 mmol) Tris-TAAD was dissolved in 0.1 ml methanol. Thirdly, 119.1 mg (0.0525 mmol) PBA-PPG-PBA was dissolved in 0.1 ml methanol. All three solutions were vortexed to get homogenous solution. The solutions were then combined and vortexed to get efficient mixing of all components. The solution was then quickly cast distributed over 3 circular silicon molds (h= 1 mm, r=10 mm). the materials were

then left to dry in the fumehood for 2 days. After drying the materials were hot-pressed between Teflon plates in a Teflon mold (h = 1 mm, r = 10 mm) at 80 °C for 1 hour.

1.15 Self-healing properties

To prepare samples for DMA studies, the networks were prepared as normal, but instead of hot-pressing for 1 hour at 80 °C in circular Teflon molds a rectangular Teflon mold (10 x 5 x 1 mm) was used instead.

Self-healing properties of the networks were tested by DMA by applying a linearly increasing extensional stress to the clamped material (10 x 5 x 0.9 mm) till breakage. After breakage occured the material was cut into pieces and hot-pressed again in a rectangular Teflon mold (10 x 5 x 1 mm) for 1 h at 80 °C.

1.16 Exchange equilibrium study

For the kinetic study of the boronic acid-TAAD exchange 0.028 mmol (15 mg) of N-methylacetate-O-phenylboronate-TAAD and 0.028 mmol of p-tolylboronic acid/4-bromophenylboronic acid/4-(trifluoromethyl)phenylboronic acid/methylboronic acid were dissolved in 500 µl d4-MeOD/D_2O 50/50. The solution was left standing for 3 days. After 3 days an ^{1}H-NMR spectrums was recorded to calculate the conversion without catalyst. Then 25 µl AcOH stock solution, prepared by adding 5.6 µl AcOH to 50 µl D_2O, was added and the mixture was shaken well. The exchange was then followed in time by measuring ^{1}H-NMR spectra every 12 minutes for 5 hours. Analysis was perfomed with the Mestrenova Reaction Monitoring software.

2 NMR data

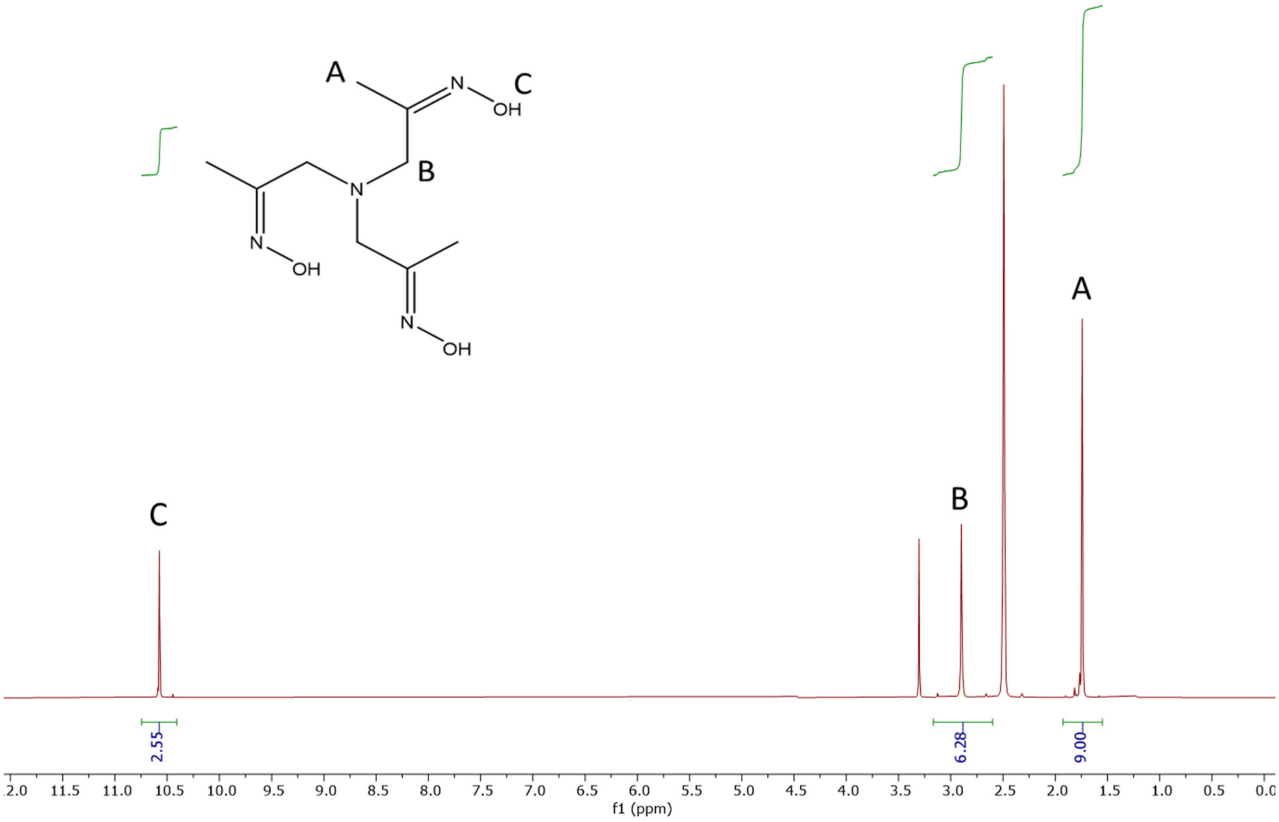

Supplementary Figure S1. ^{1}H-NMR of $TRISOXH_3$ in d6-DMSO

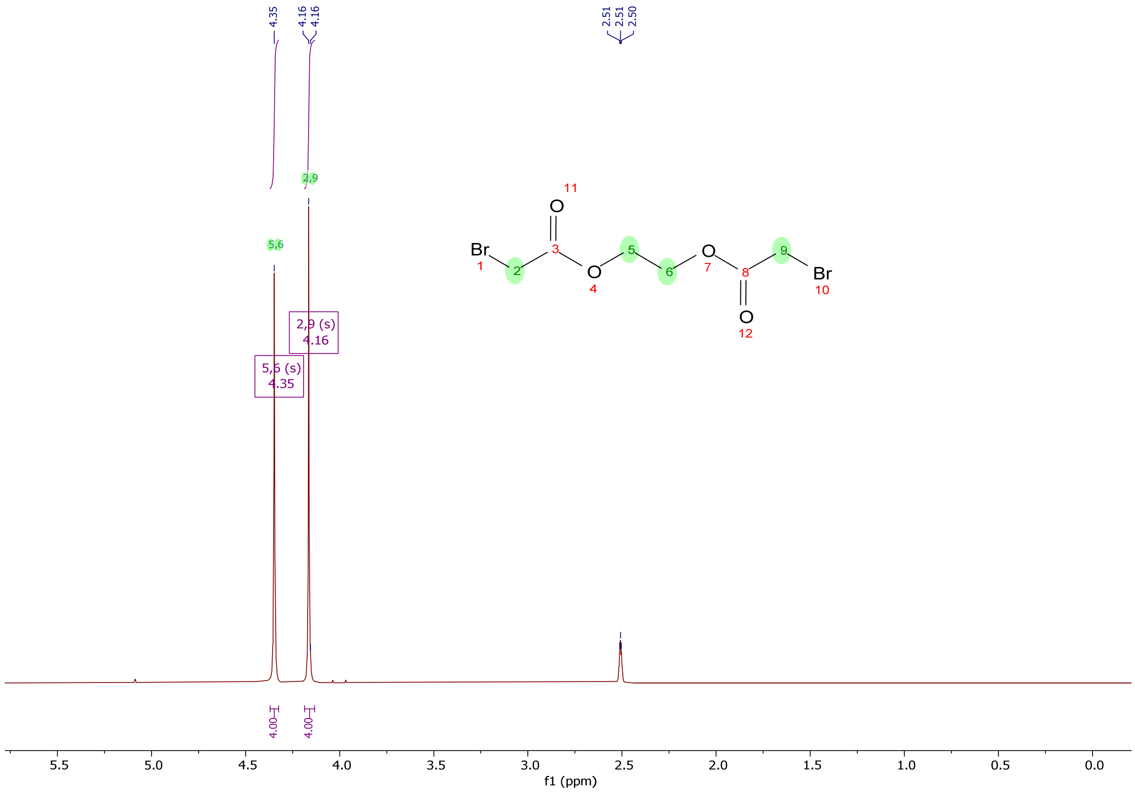

Supplementary Figure S2. ^{1}H-NMR of bis(bromoacetoxy)ethane in d6-DMSO

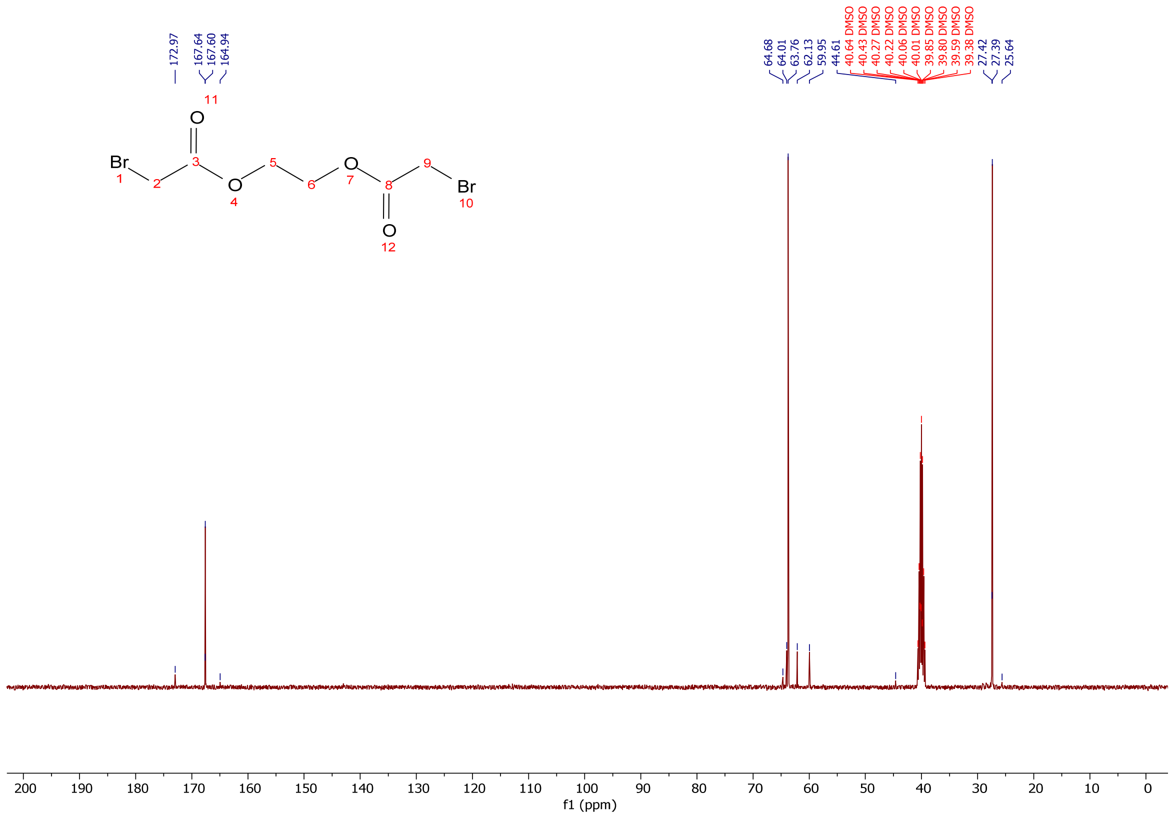

Supplementary Figure S3. ^{13}C-NMR of bis(bromoacetoxy)ethane in d6-DMSO

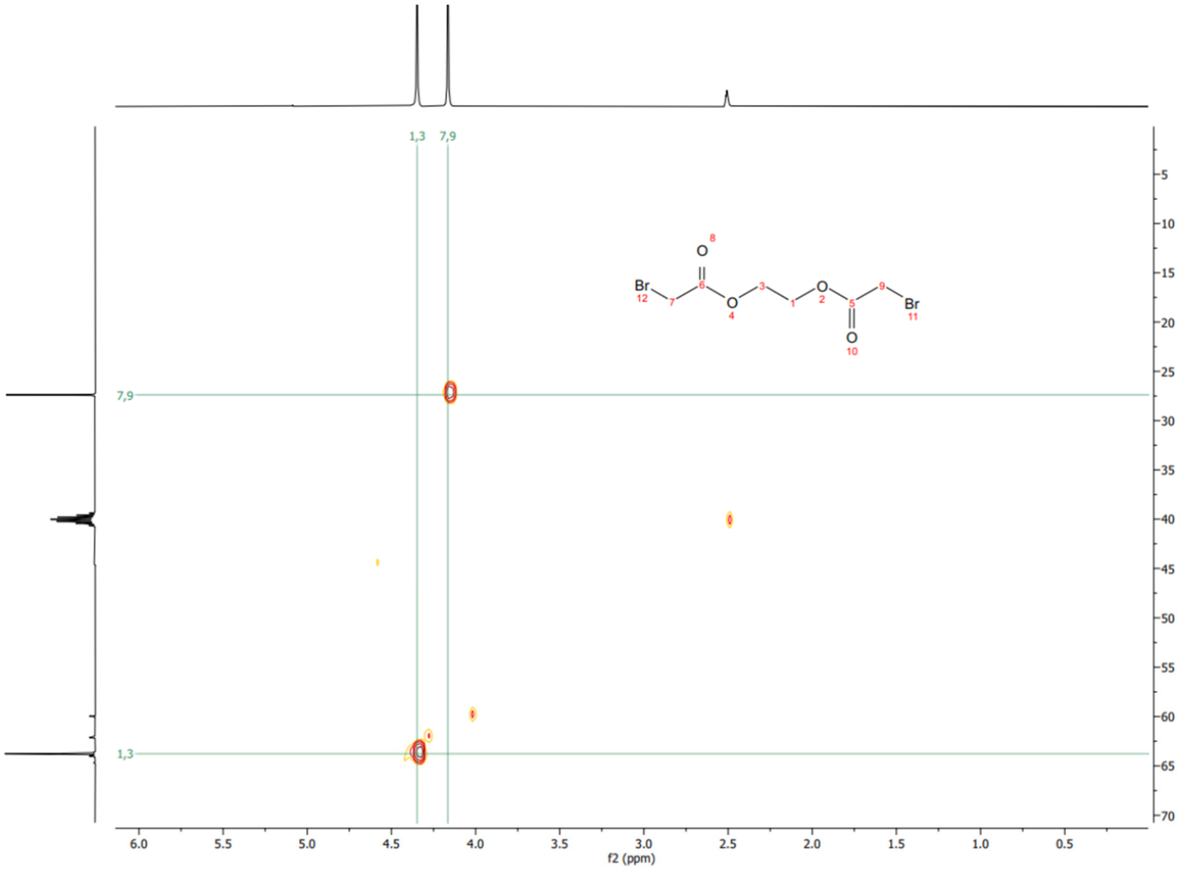

Supplementary Figure S4. COSY of bis(bromoacetoxy)ethane in d6-DMSO

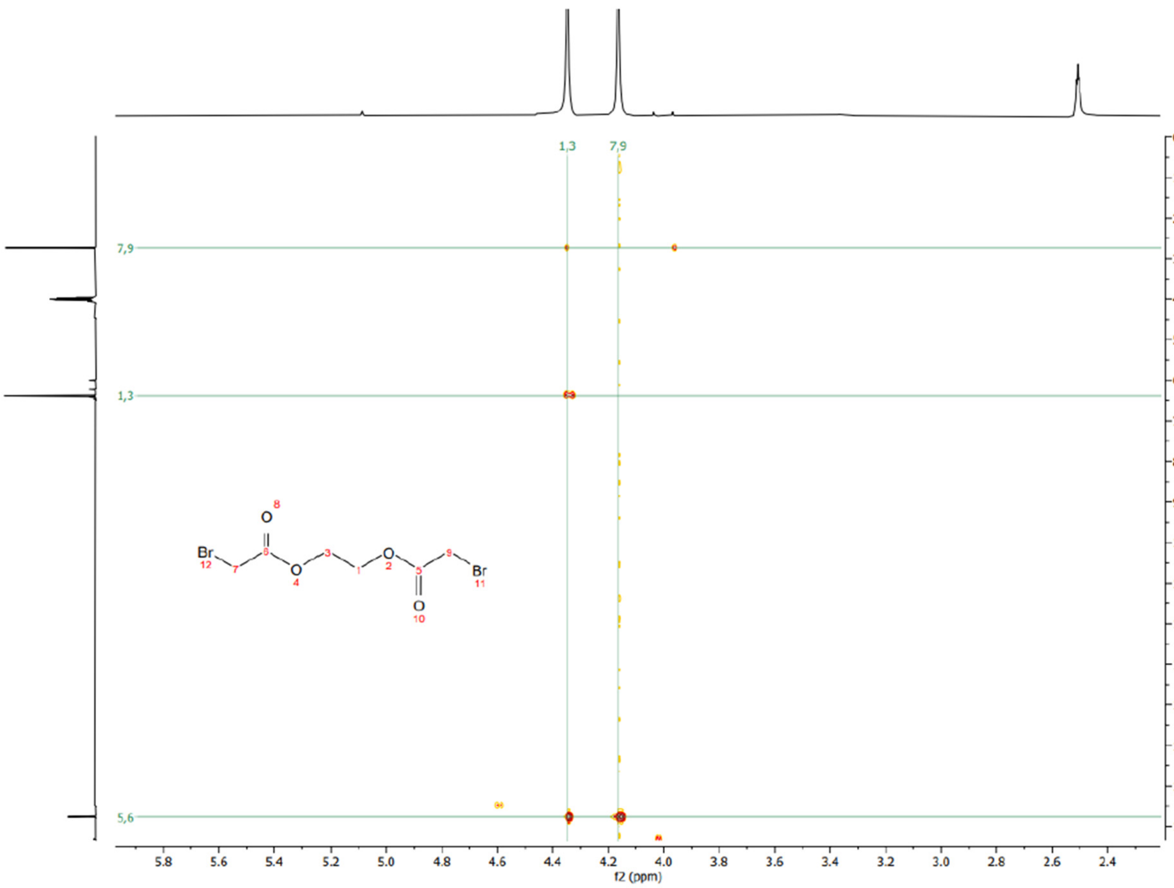

Supplementary Figure S5. HMBC of bis(bromoacetoxy)ethane in d6-DMSO

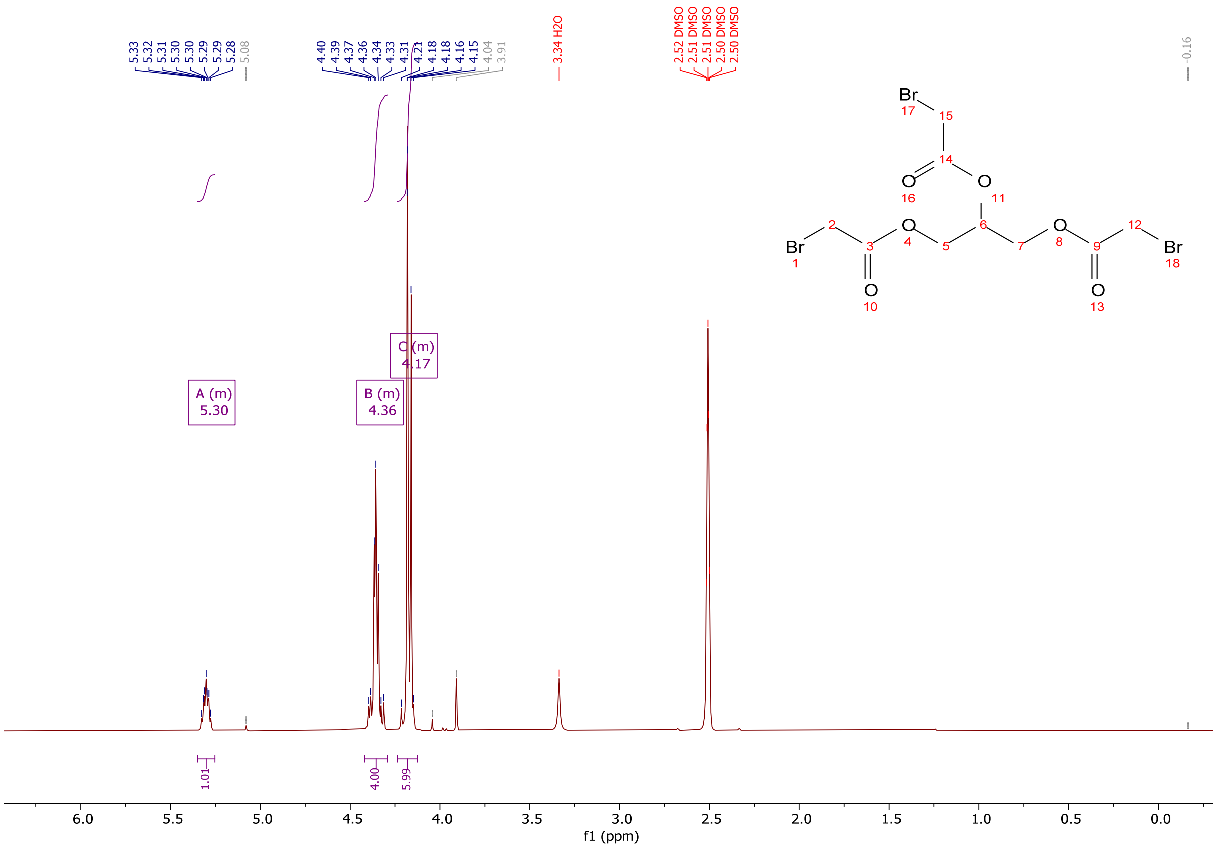

Supplementary Figure S6. ^{1}H-NMR of tris(bromoacetoxy)propane in d6-DMSO

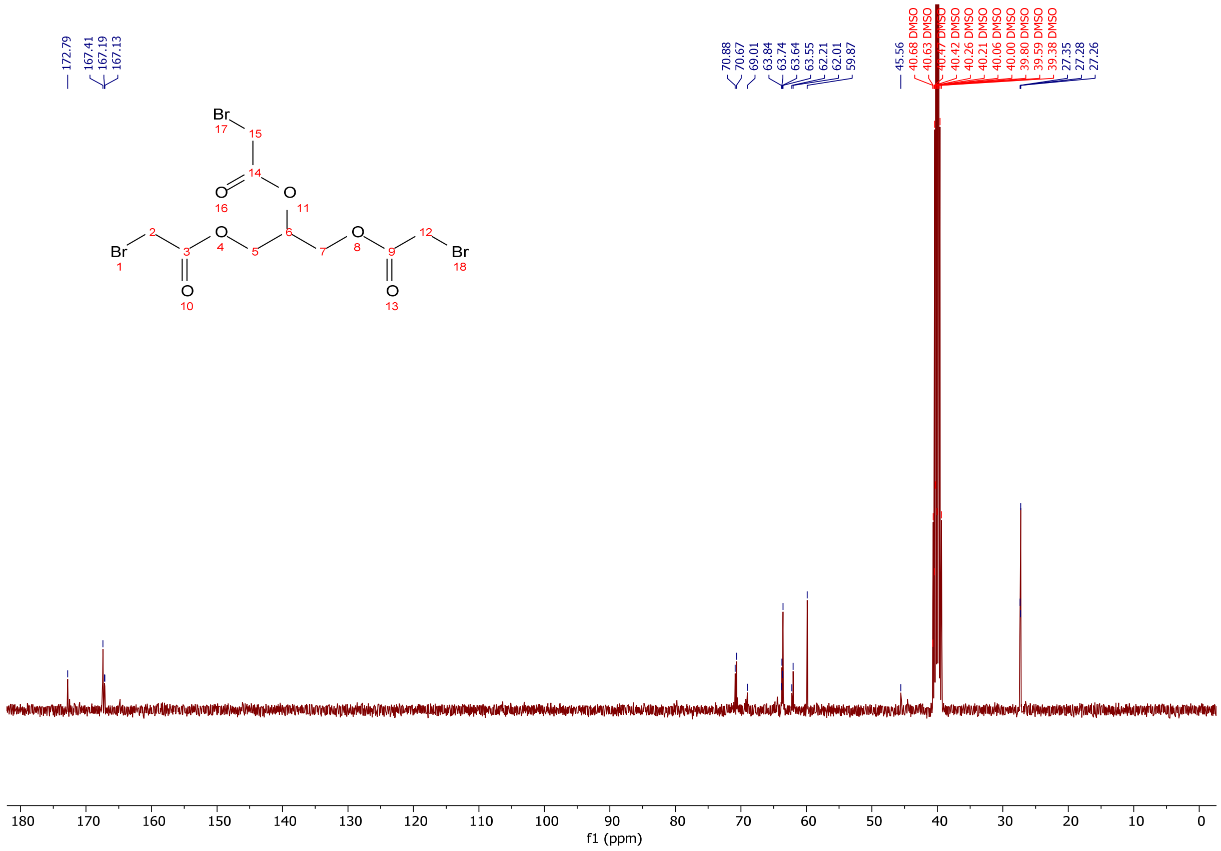

Supplementary Figure S7. ^{13}C-NMR of tris(bromoacetoxy)propane in d6-DMSO

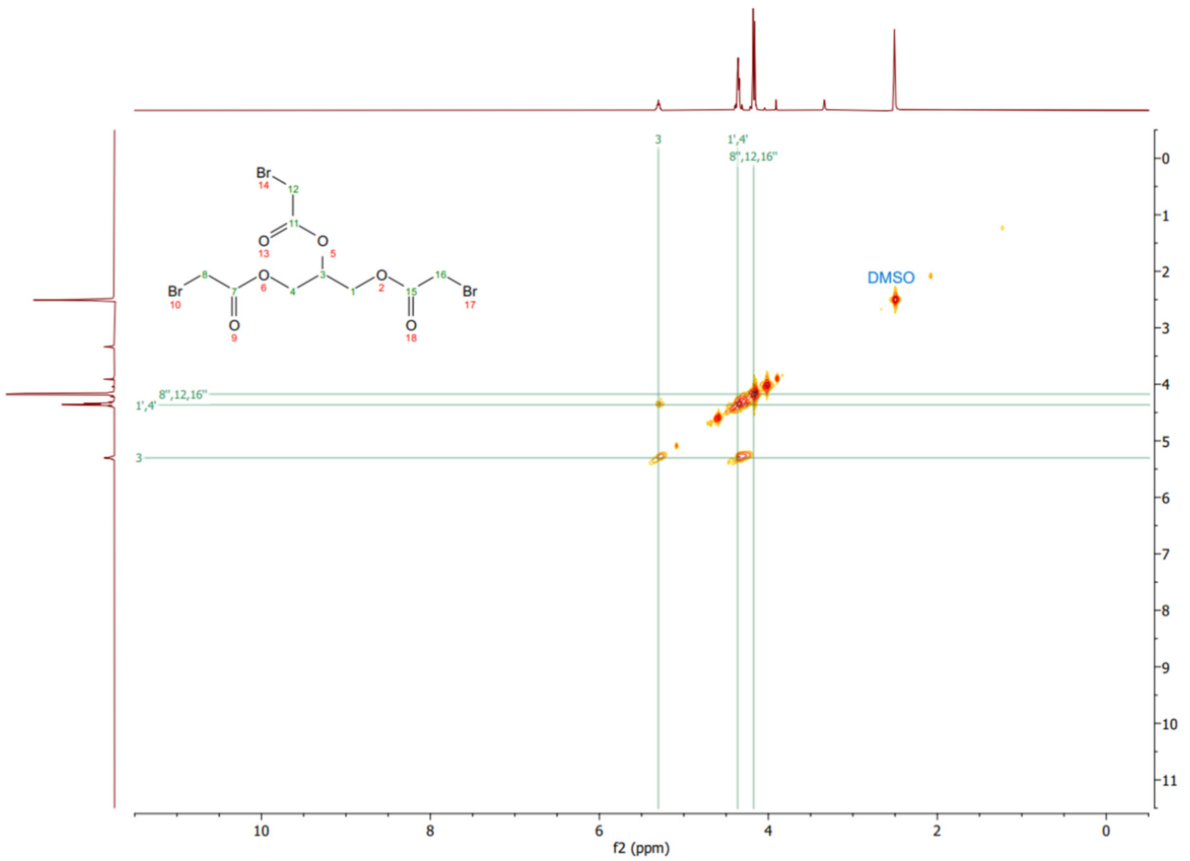

Supplementary Figure S8. COSY of tris(bromoacetoxy)propane in d6-DMSO

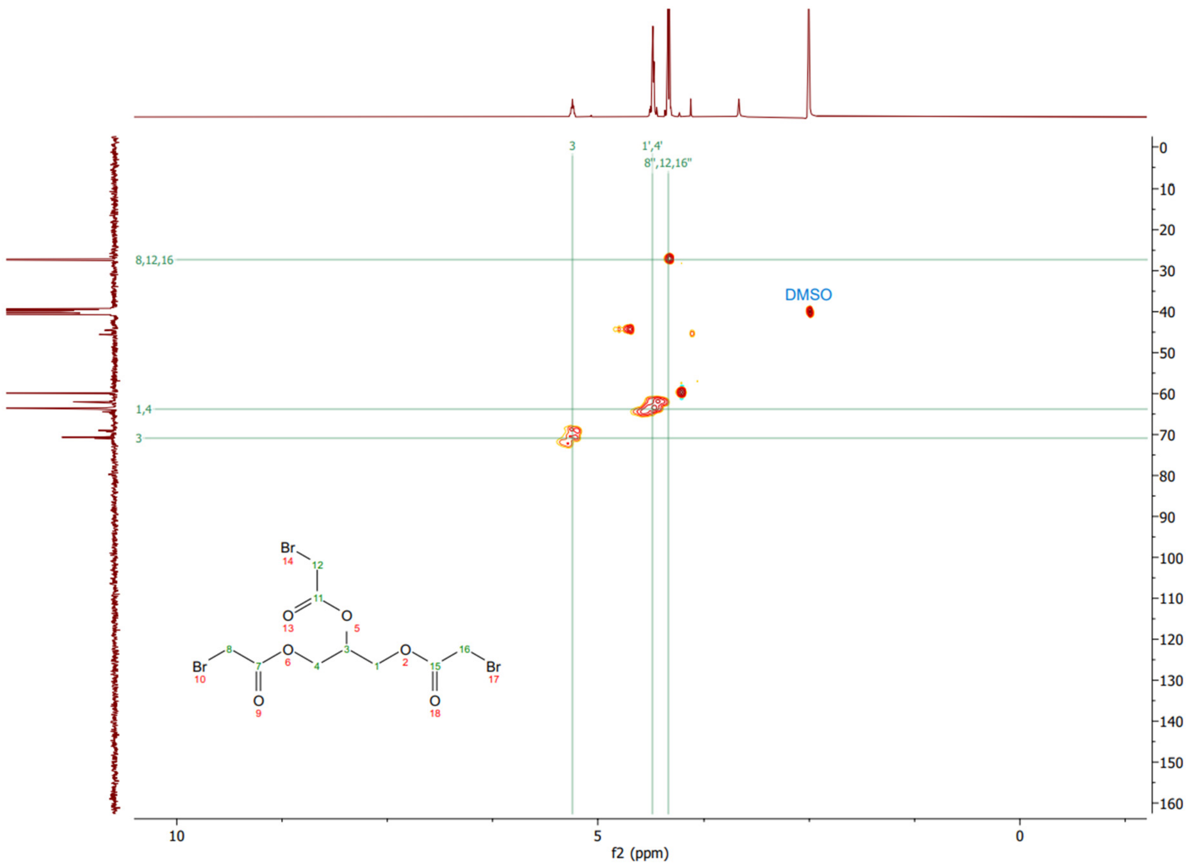

Supplementary Figure S9. HSQC of tris(bromoacetoxy)propane in d6-DMSO

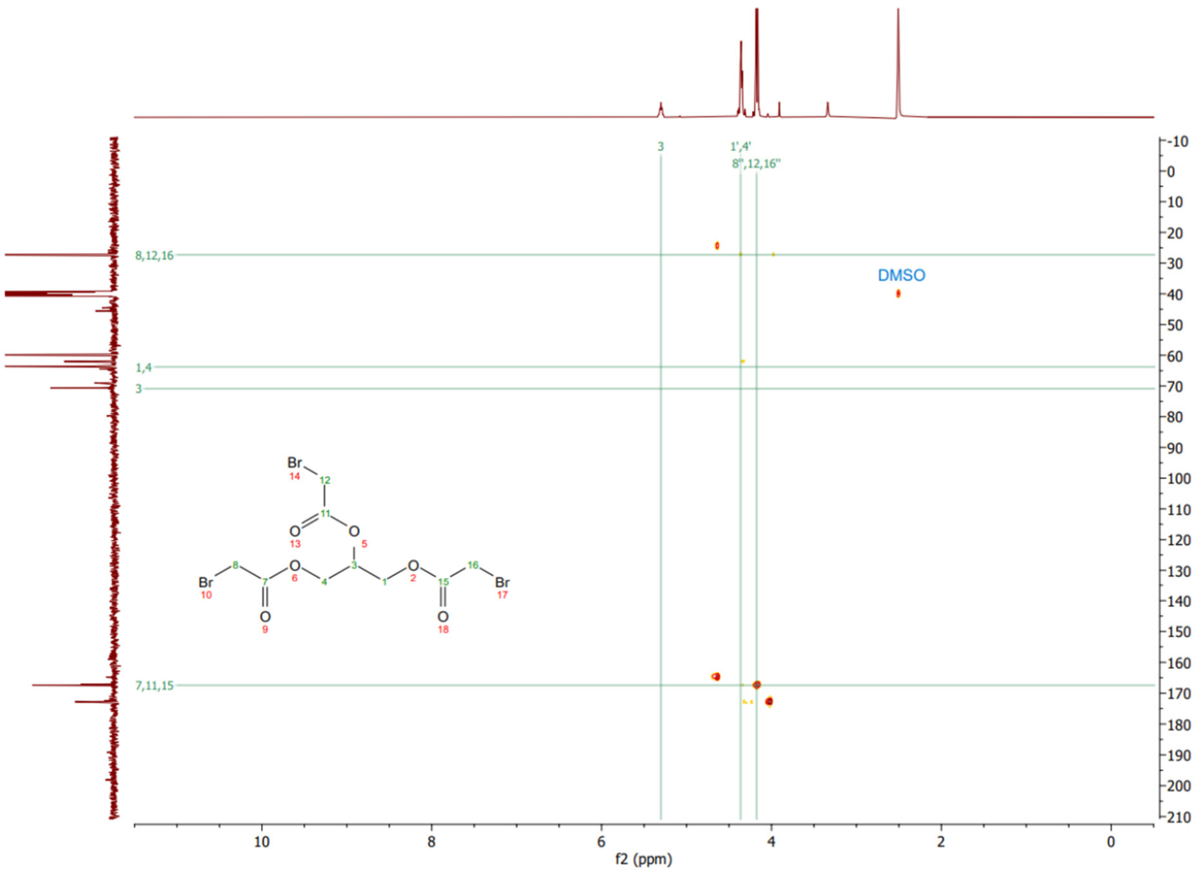

Supplementary Figure S10. HMBC of tris(bromoacetoxy)propane in d6-DMSO

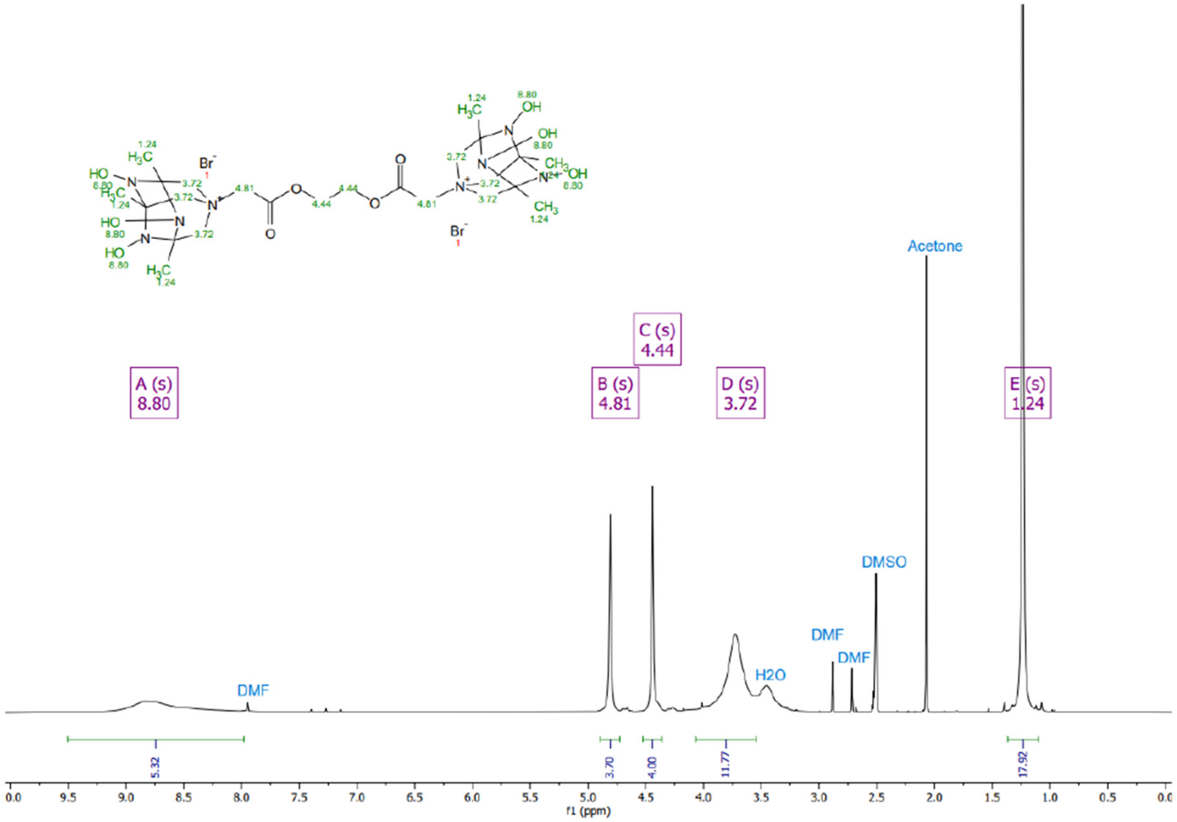

Supplementary Figure S11. ^{1}H-NMR spectrum of bis(TAADacetoxy)ethane in d6-DMSO

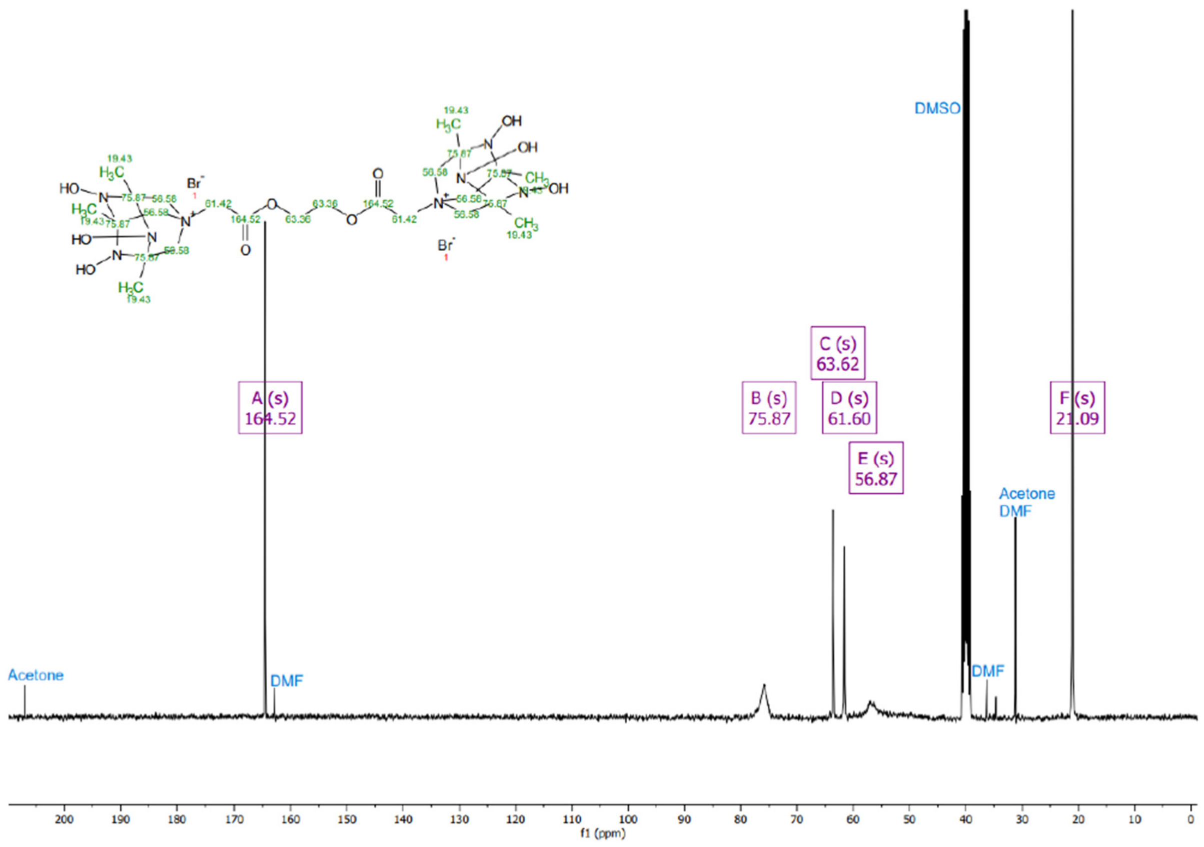

Supplementary Figure S12. ^{13}C-NMR spectrum of bis(TAADacetoxy)ethane in d6-DMSO

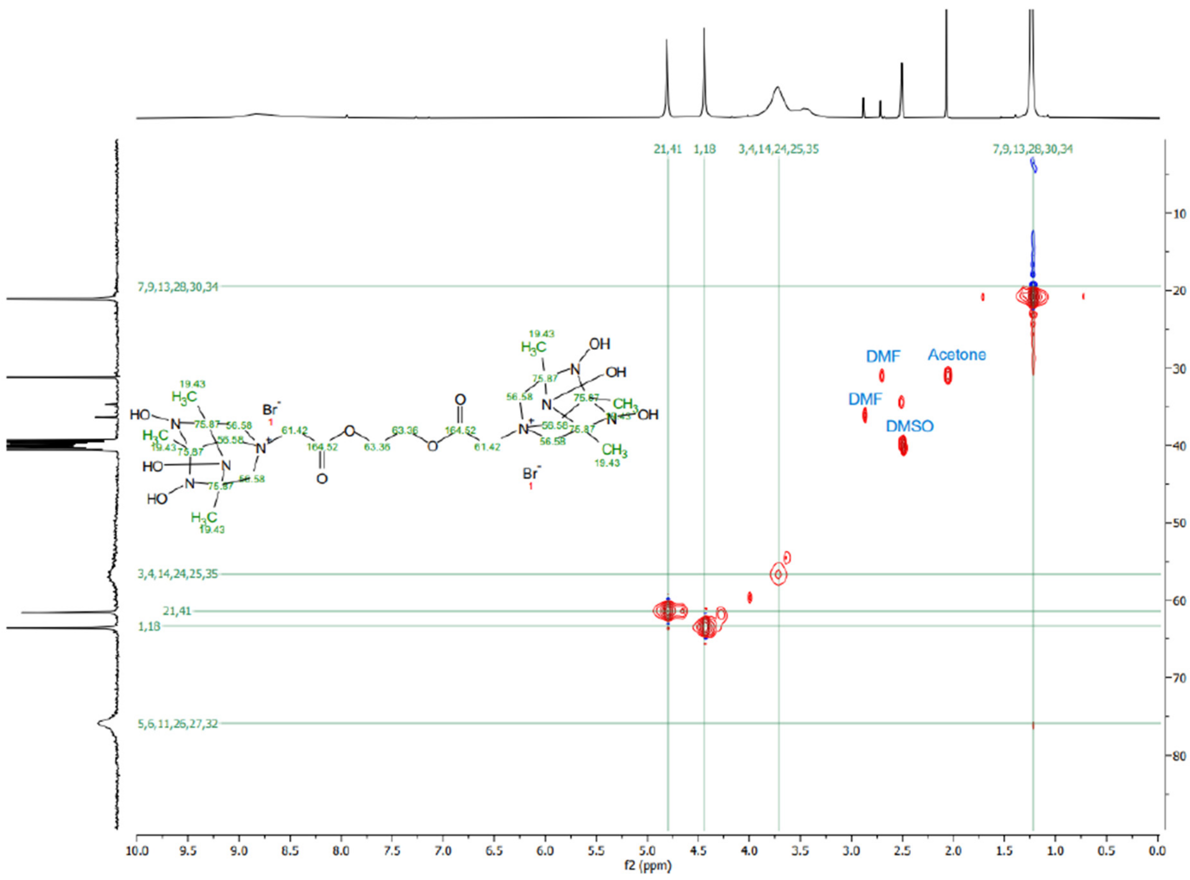

Supplementary Figure S13. HSQC of bis(TAADacetoxy)ethane in d6-DMSO

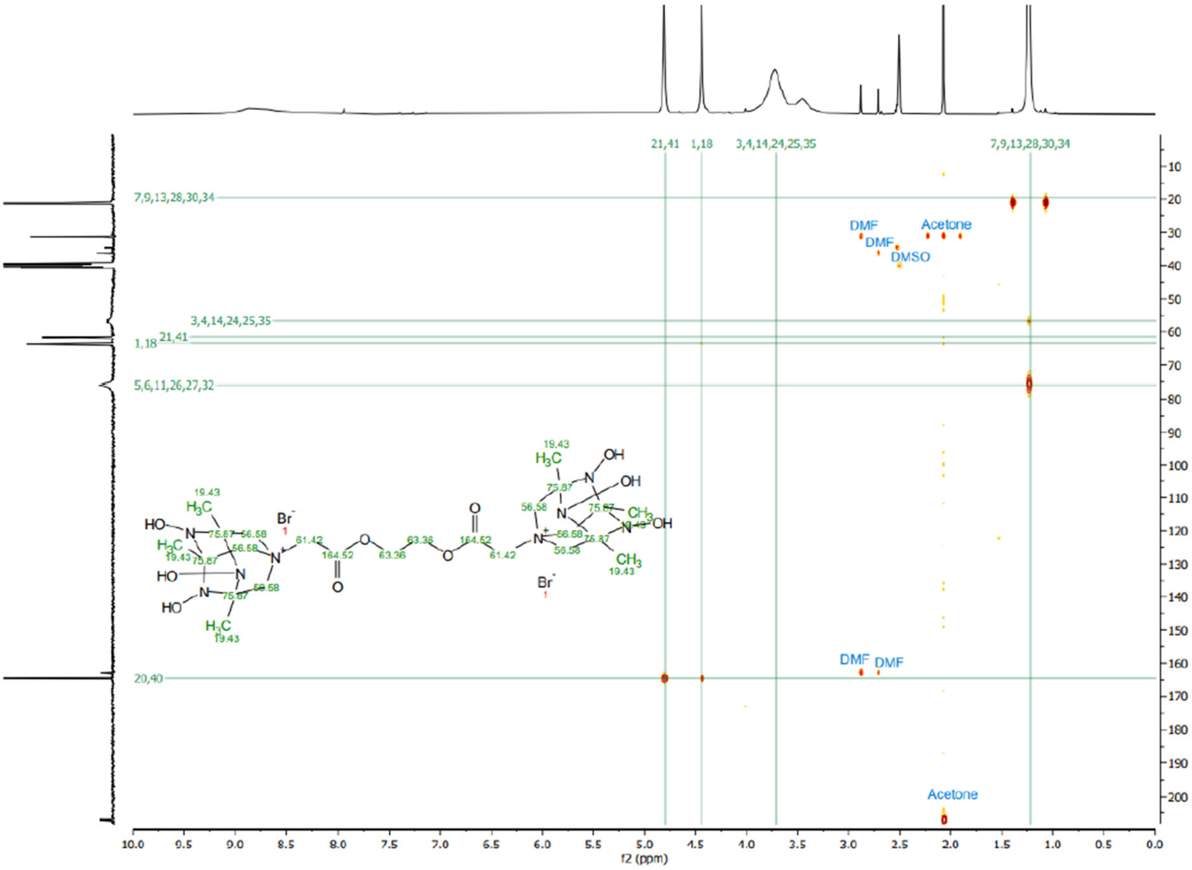

Supplementary Figure S14. HMBC of bis(TAADacetoxy)ethane in d6-DMSO

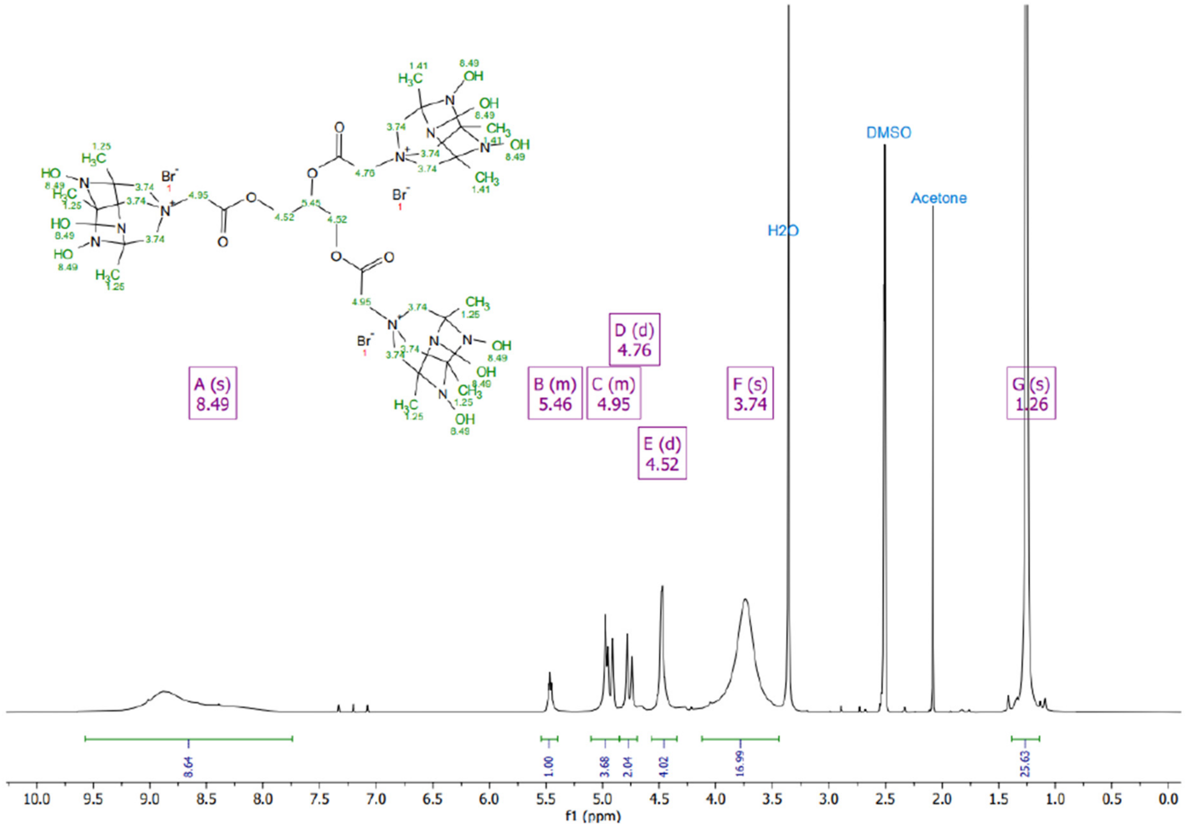

Supplementary Figure S15. ^{1}H-NMR spectrum of tris(TAADacetoxy)propane in d6-DMSO

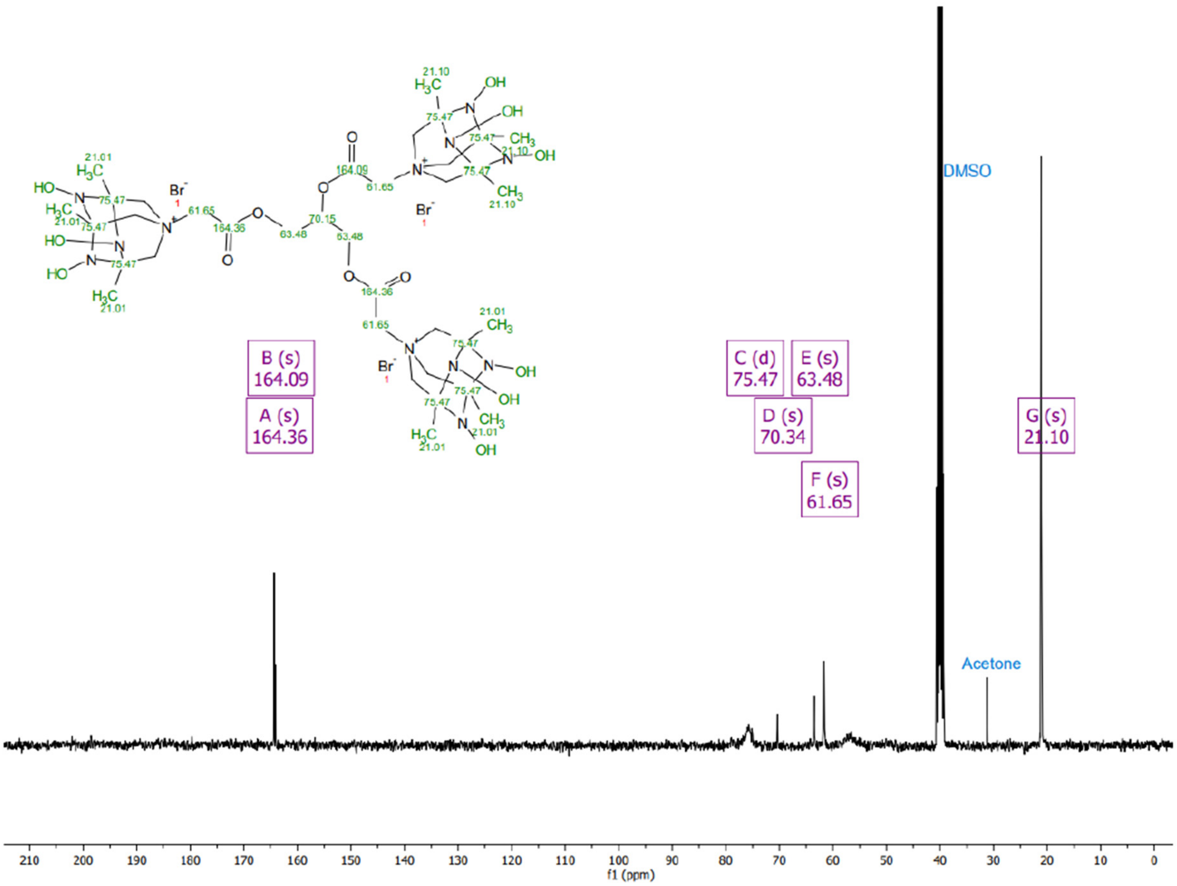

Supplementary Figure S16. ^{13}C-NMR spectrum of tris(TAADacetoxy)propane in d6-DMSO

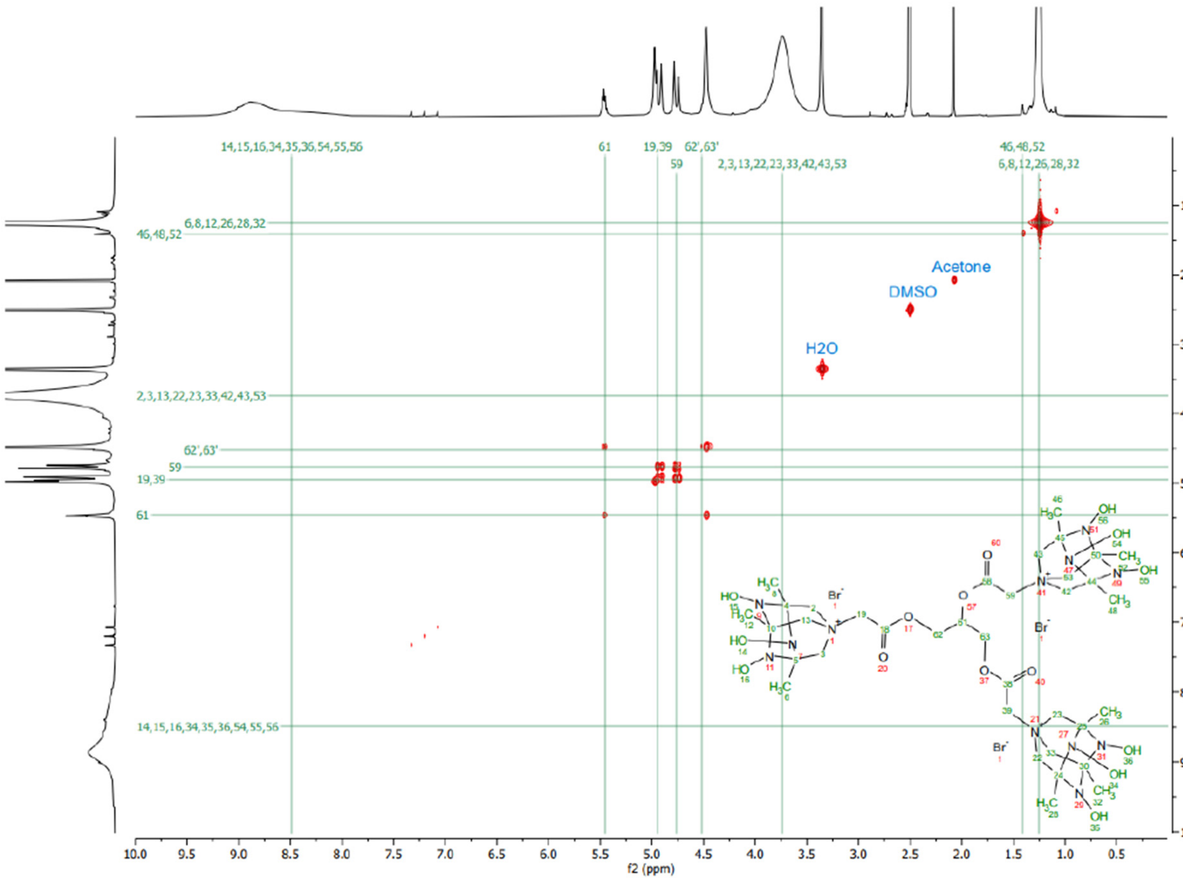

Supplementary Figure S17. COSY of tris(TAADacetoxy)propane in d6-DMSO

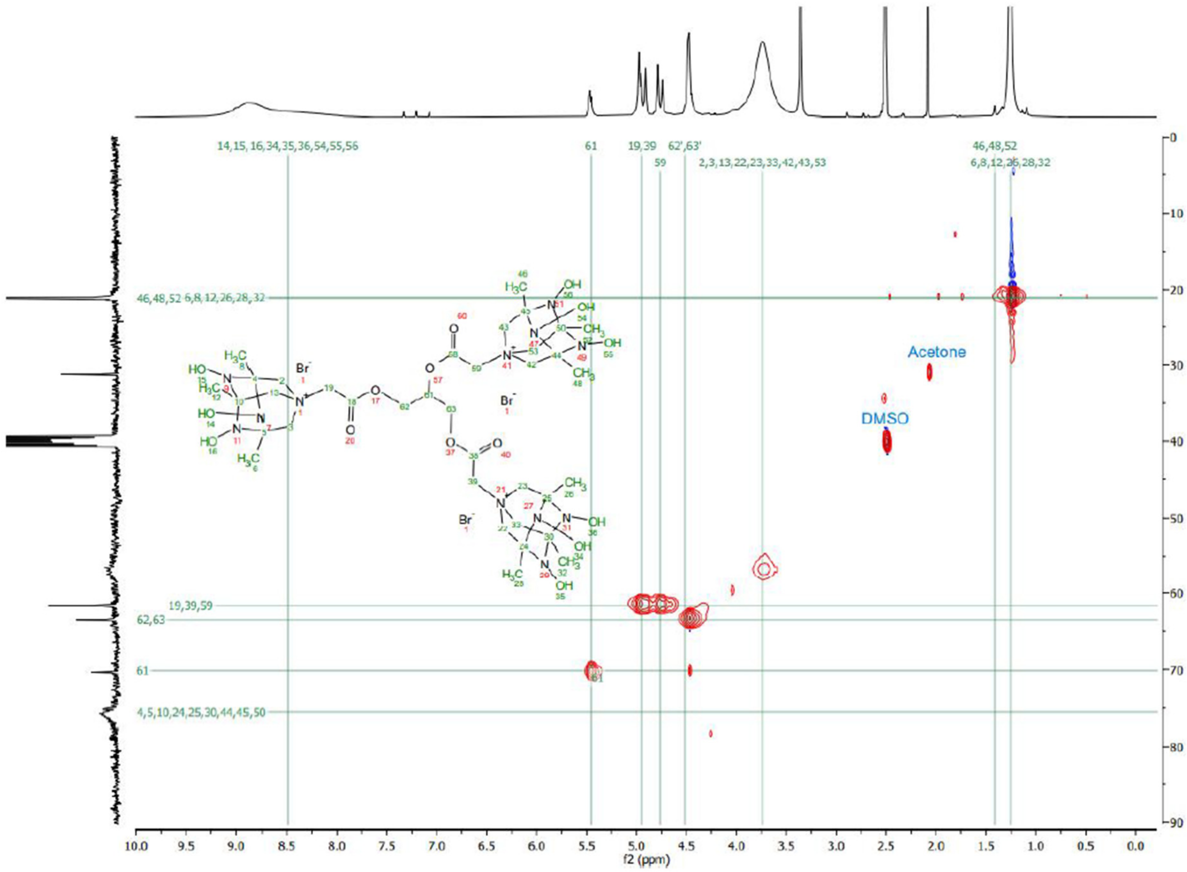

Supplementary Figure S18. HSQC of tris(TAADacetoxy)propane in d6-DMSO

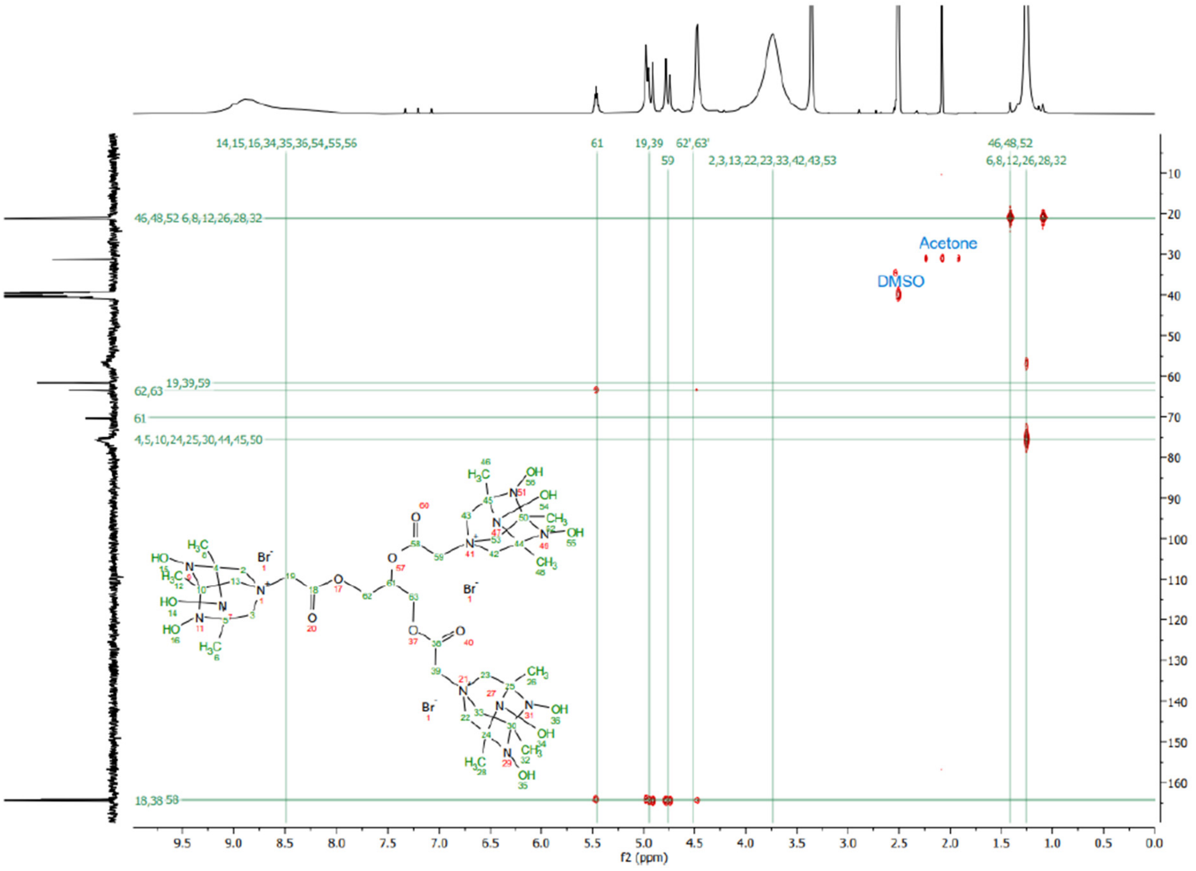

Supplementary Figure S19. HMBC of tris(TAADacetoxy)propane in d6-DMSO

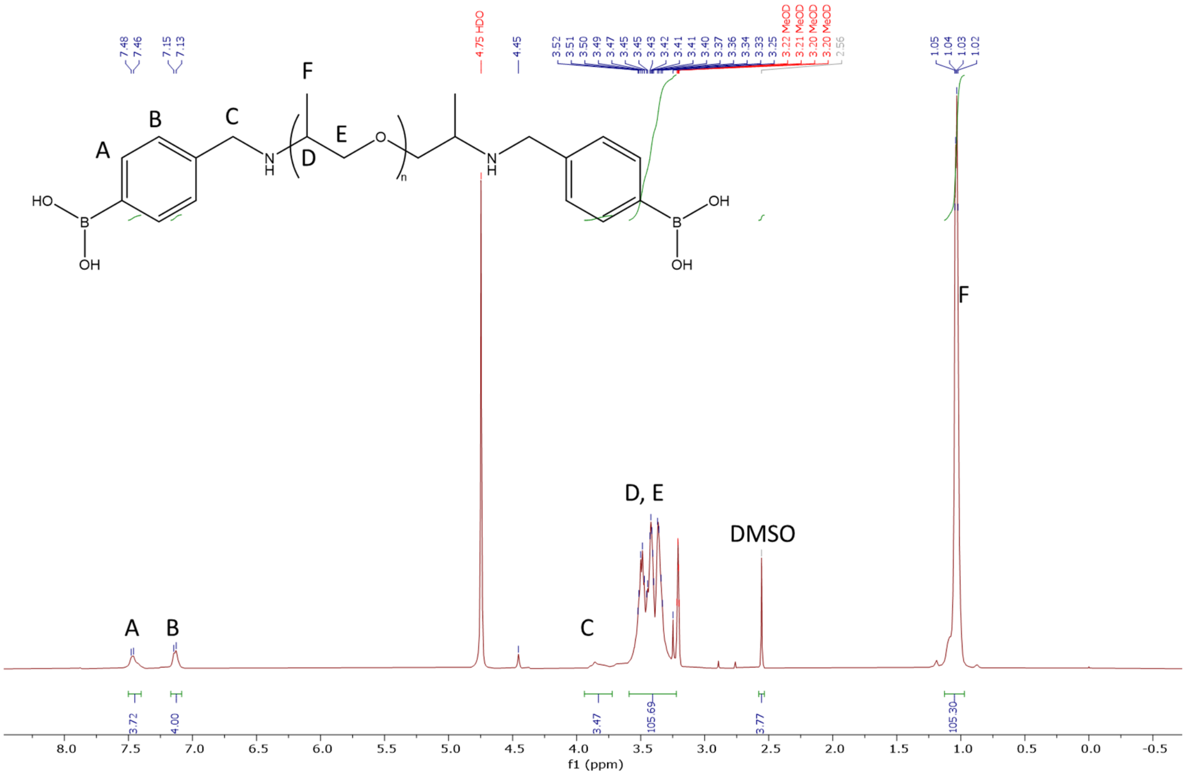

Supplementary Figure S20. ^{1}H-NMR spectrum of PBA-PPG-PBA

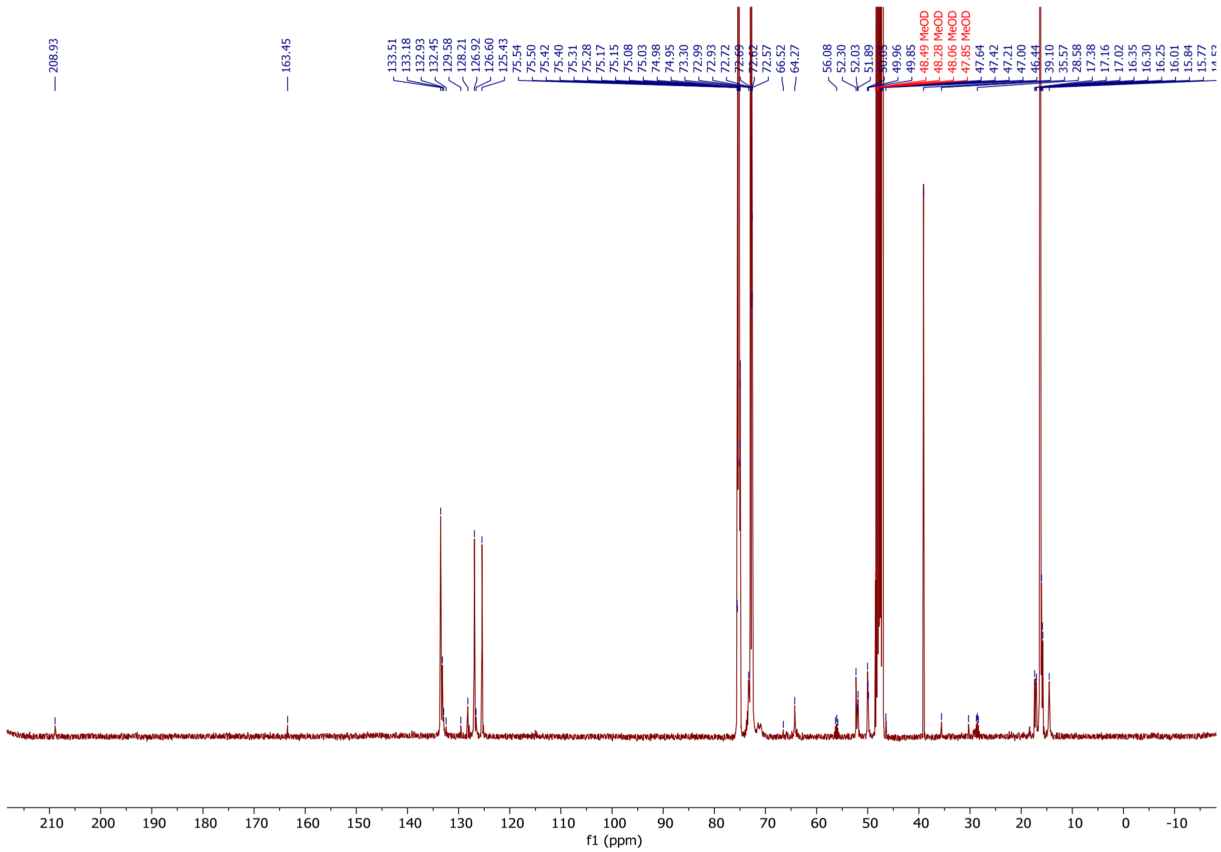

Supplementary Figure S21. ^{13}C-NMR spectrum of PBA-PPG-PBA

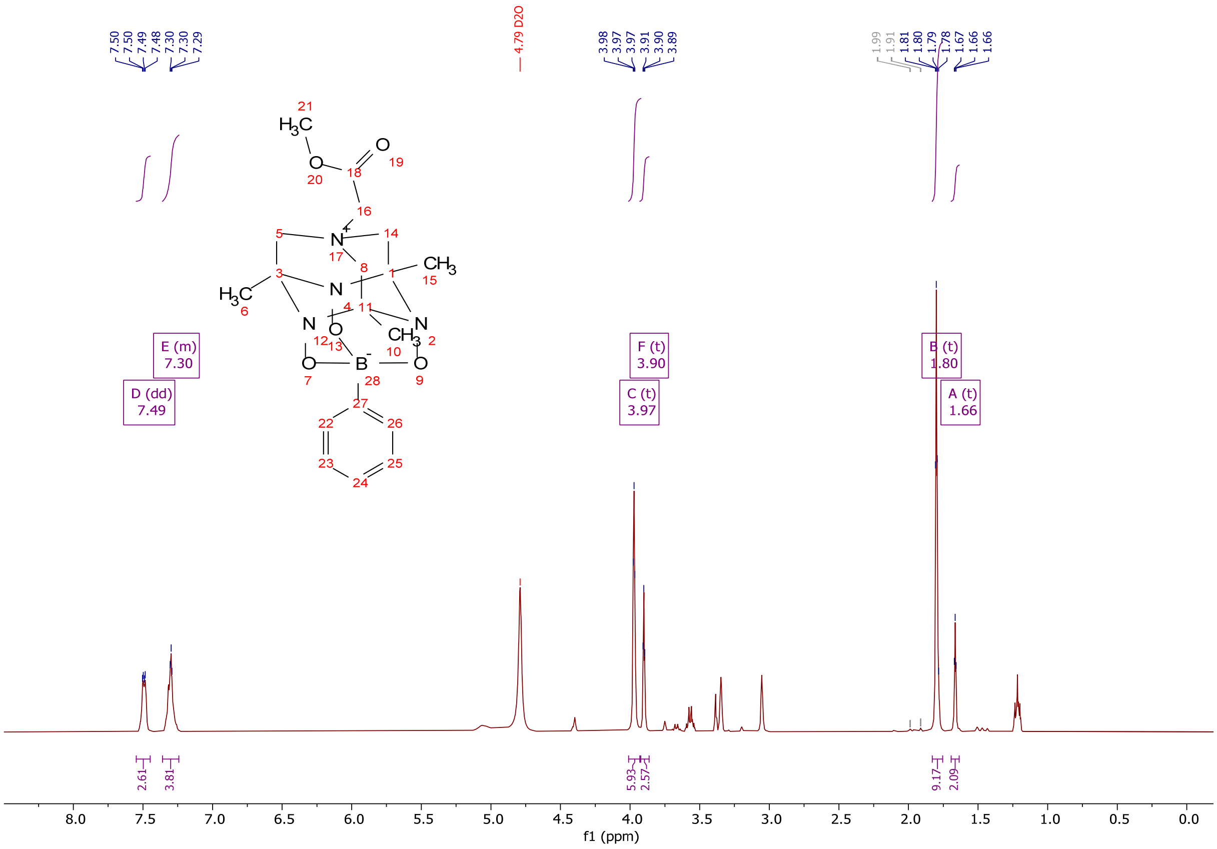

Supplementary Figure S22. ^{1}H-NMR spectrum of N-methylacetate-O-phenylboronate-TAAD

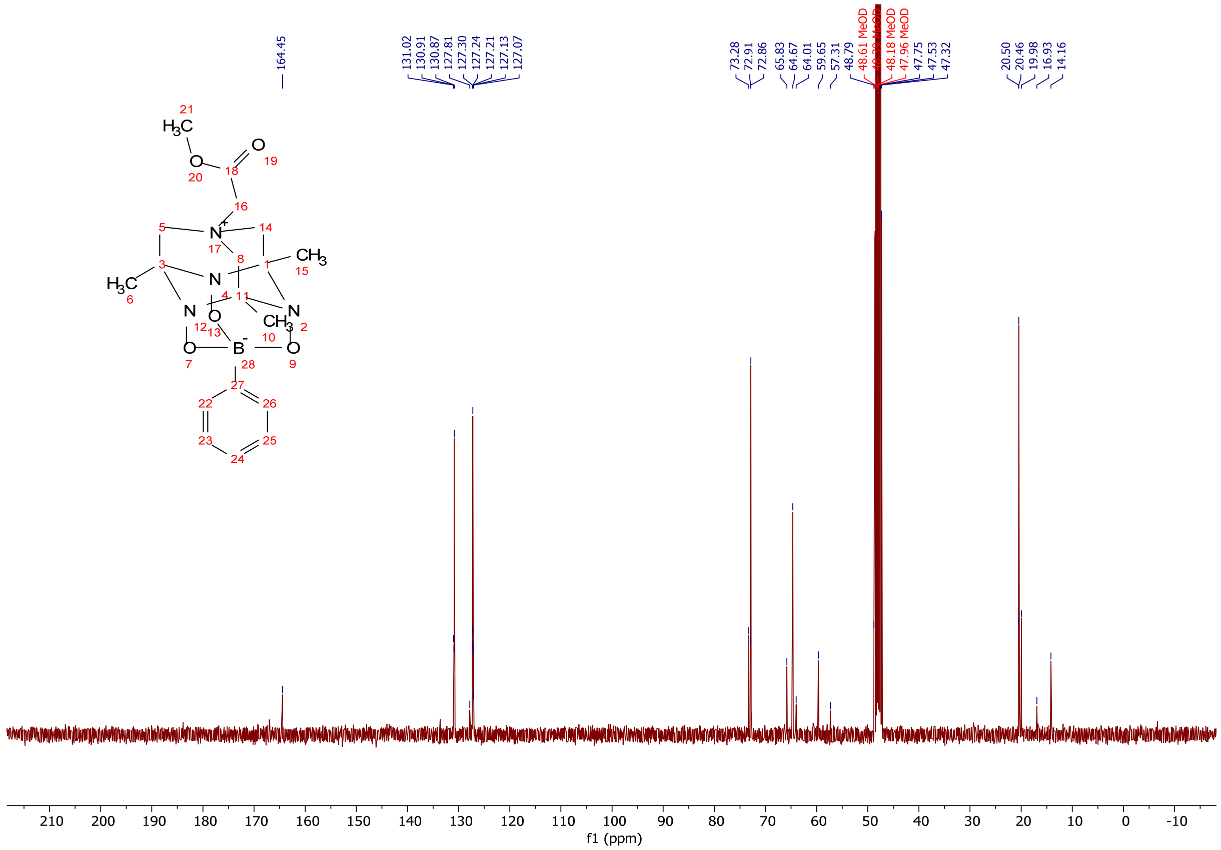

Supplementary Figure S23. ^{13}C-NMR spectrum of N-methylacetate-O-phenylboronate-TAAD

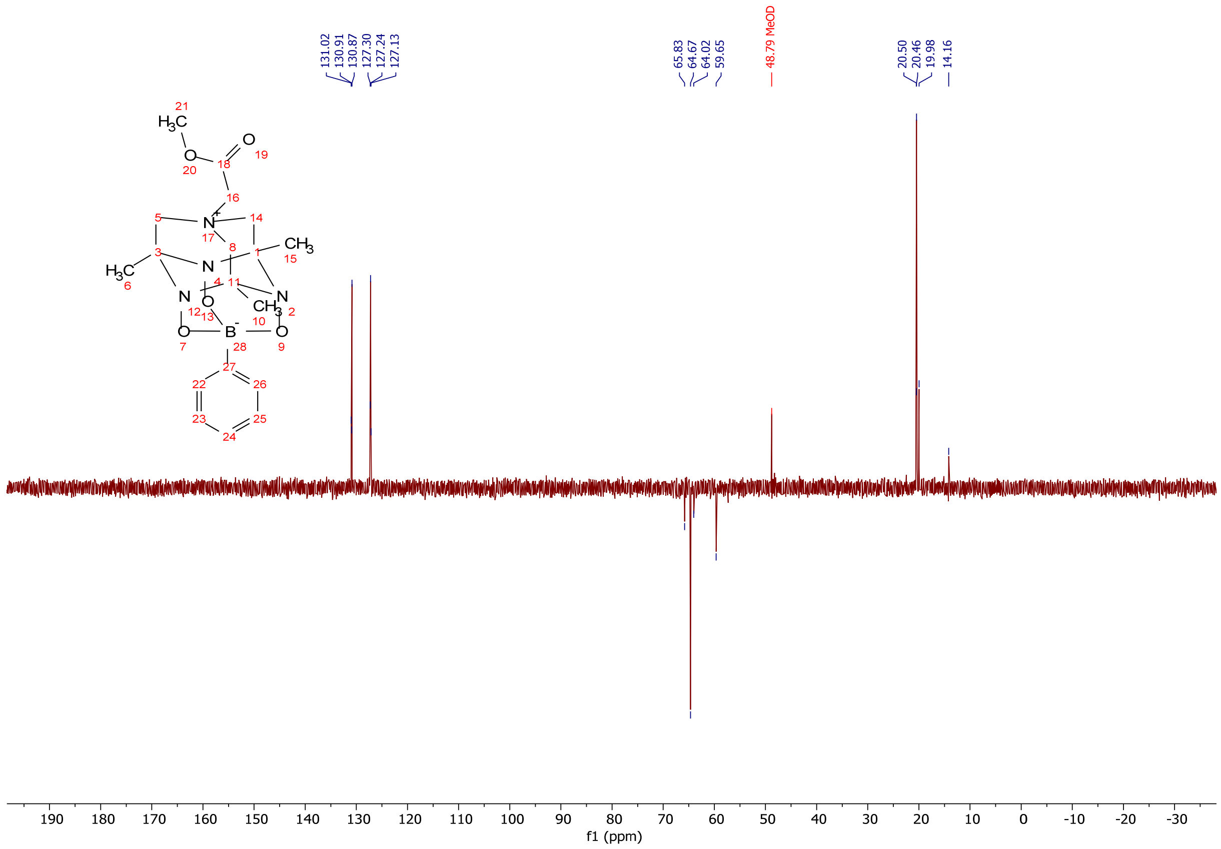

Supplementary Figure S24. DEPT-135 spectrum of N-methylacetate-O-phenylboronate-TAAD

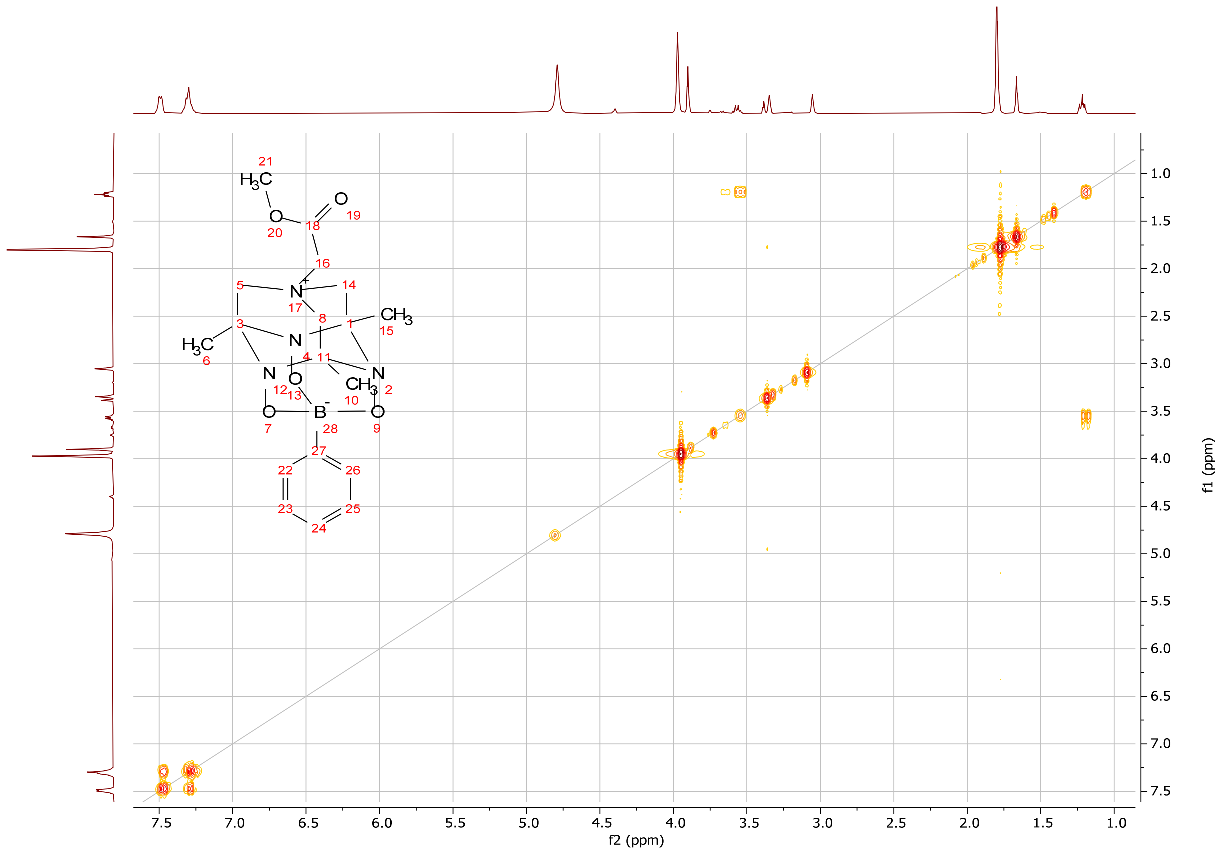

Supplementary Figure S25. COSY of N-methylacetate-O-phenylboronate-TAAD

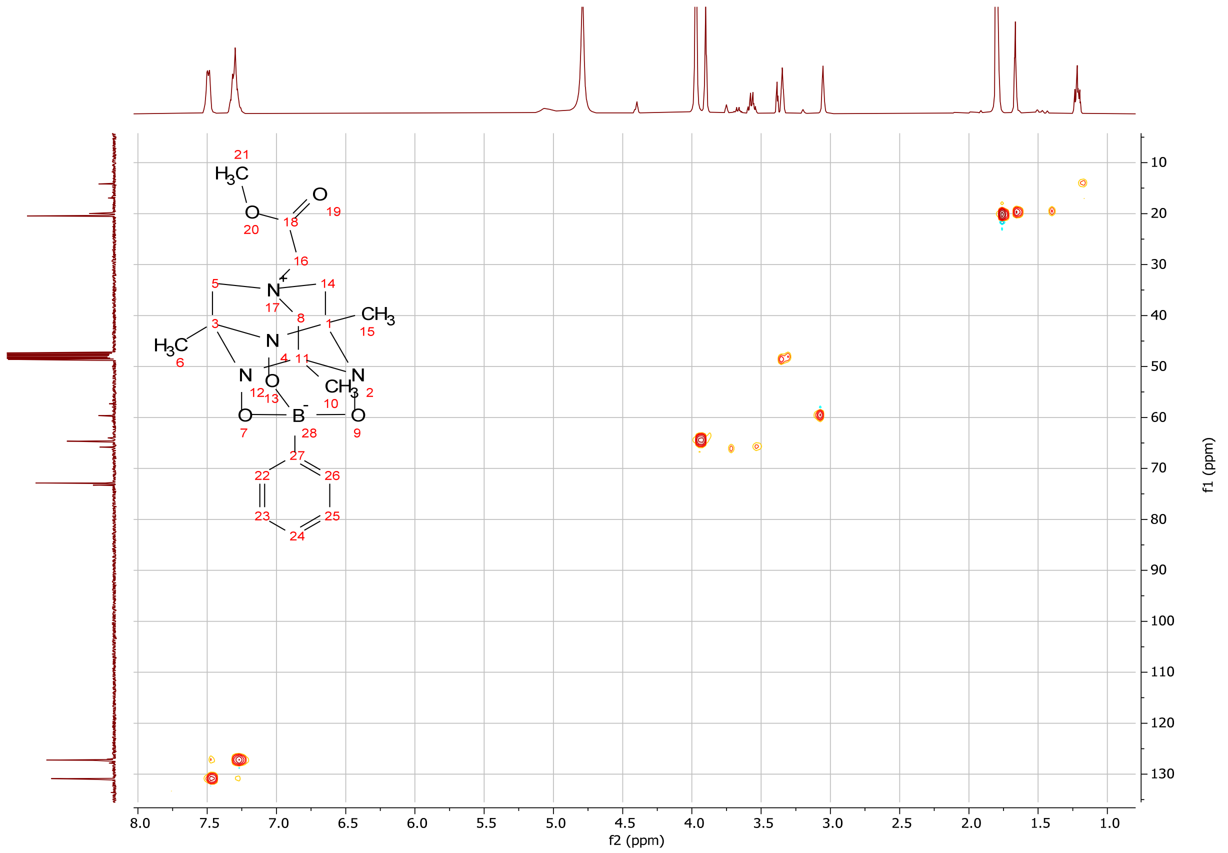

Supplementary Figure S26. HSQC of N-methylacetate-O-phenylboronate-TAAD

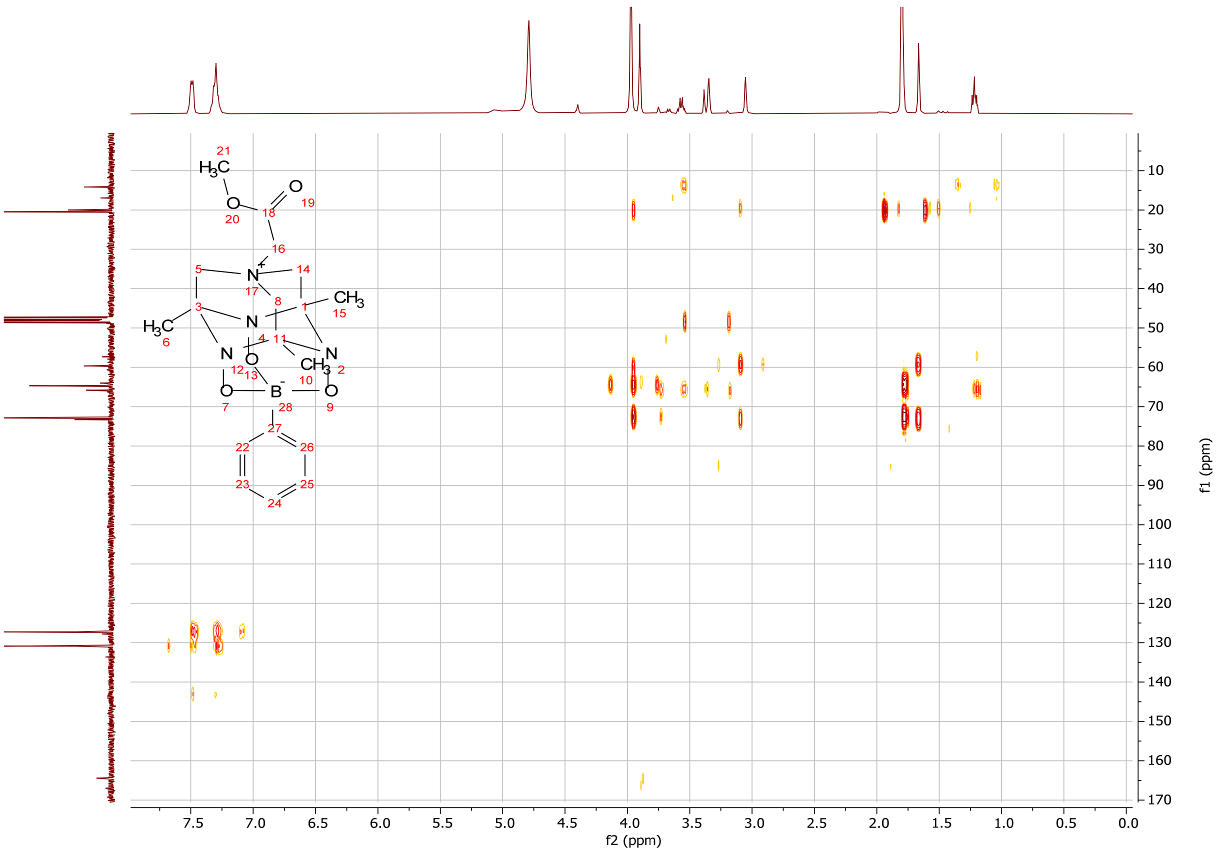

Supplementary Figure S27. HMBC of N-methylacetate-O-phenylboronate-TAAD

3 IR data

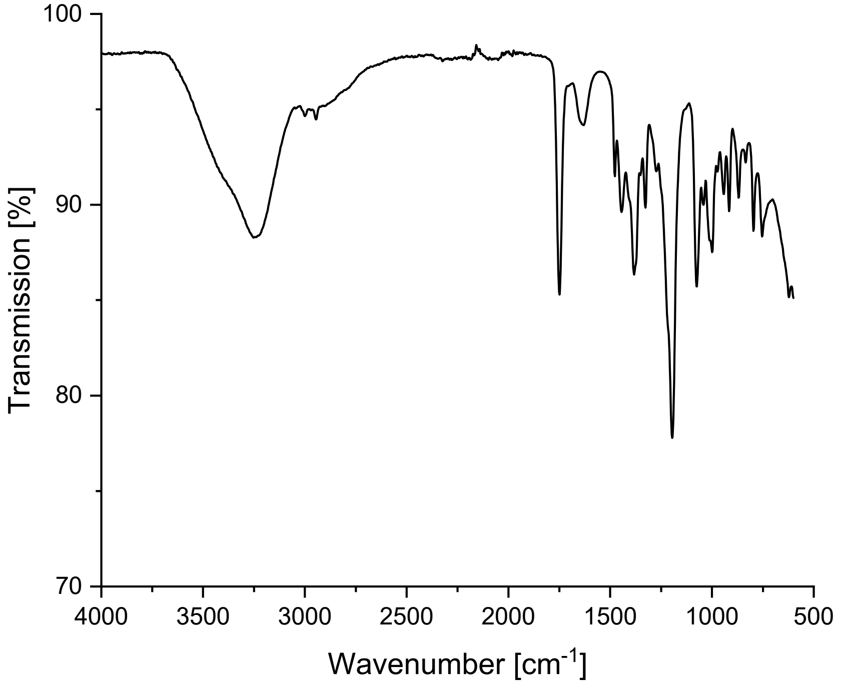

Supplementary Figure S28. IR spectrum of bis(TAADacetoxy)ethane

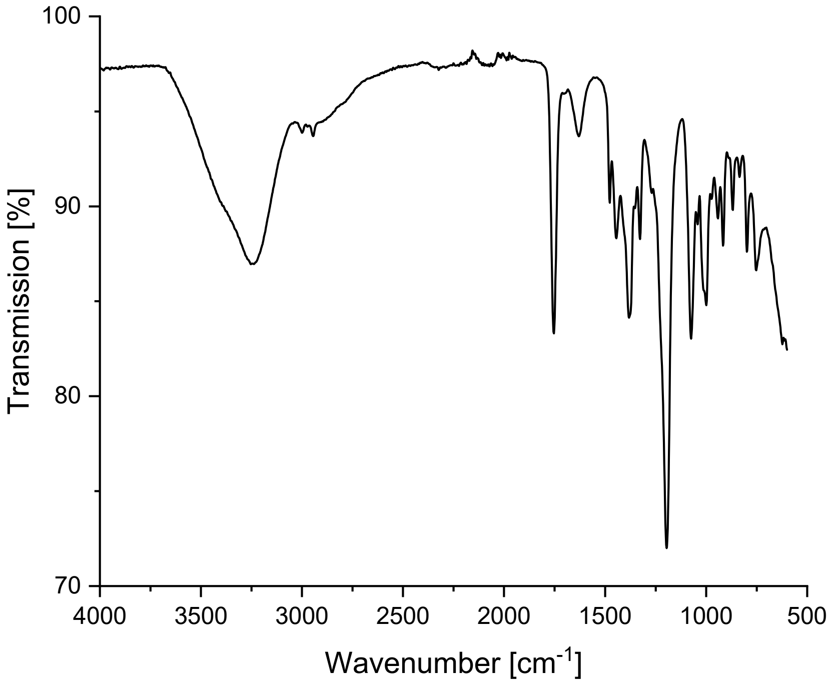

Supplementary Figure S29. IR spectrum of tris(TAADacetoxy)propane

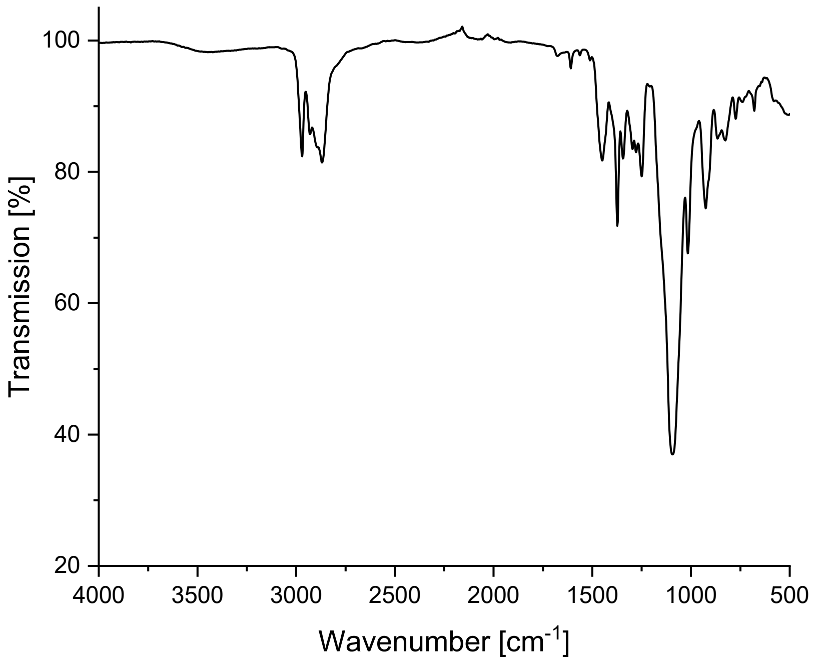

Supplementary Figure S30. IR spectrum of PBA-PPG-PBA

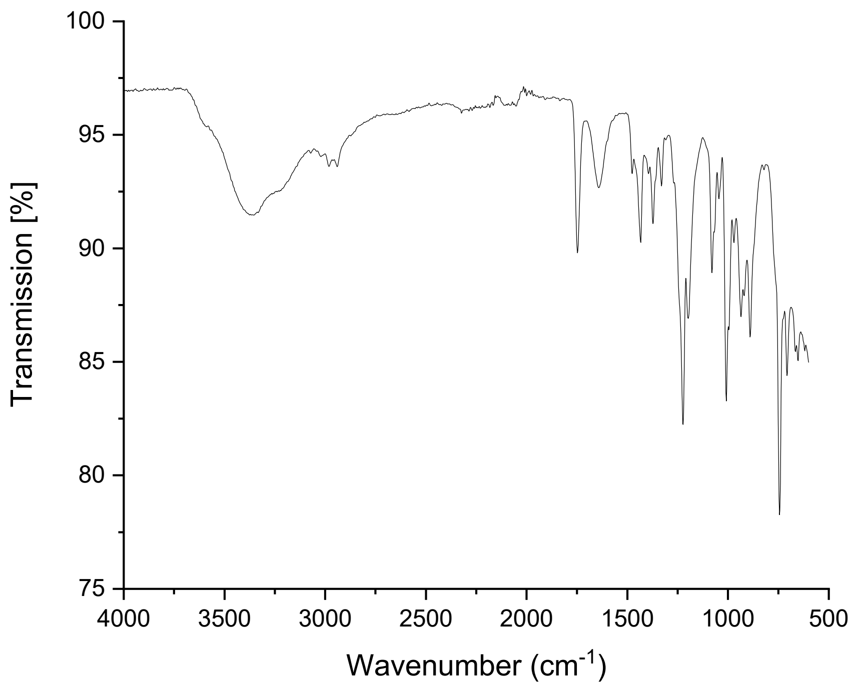

Supplementary Figure S31. IR spectrum of N-methylacetate-O-phenylboronate-TAAD

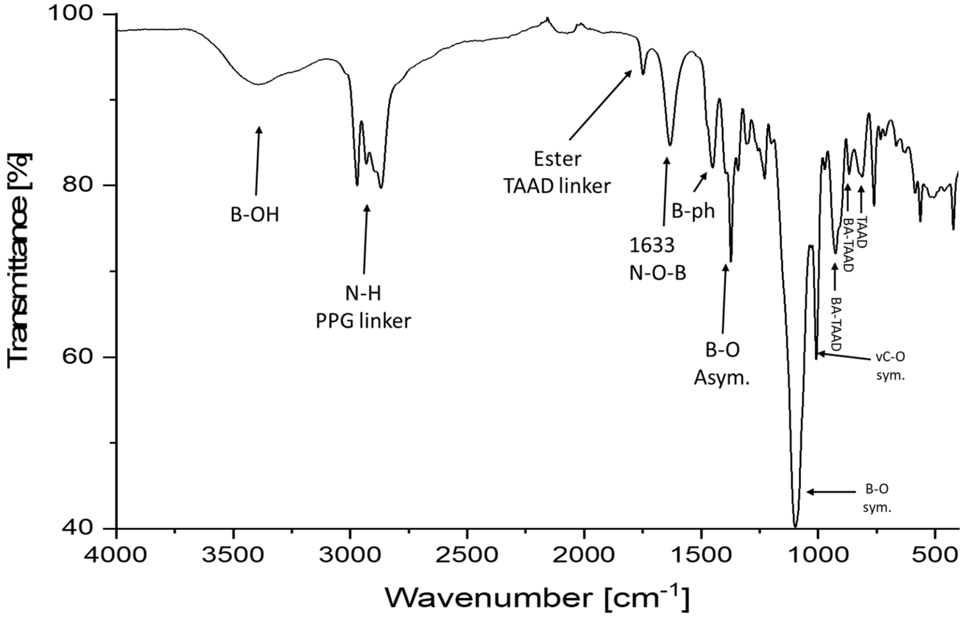

Supplementary Figure S32. assigned IR spectrum of a 33% crosslinked boronate-TAAD network

4 Mass spectrometry data

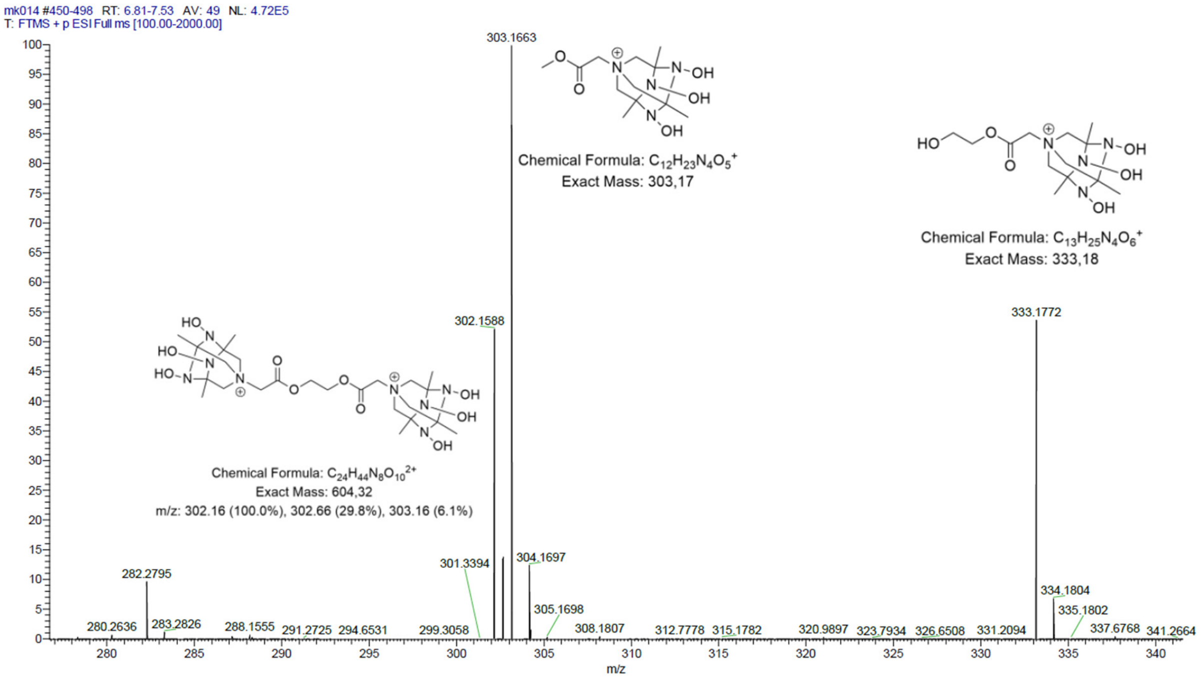

Supplementary Figure S33. mass spectrum of bis(TAAD acetoxy)ethane

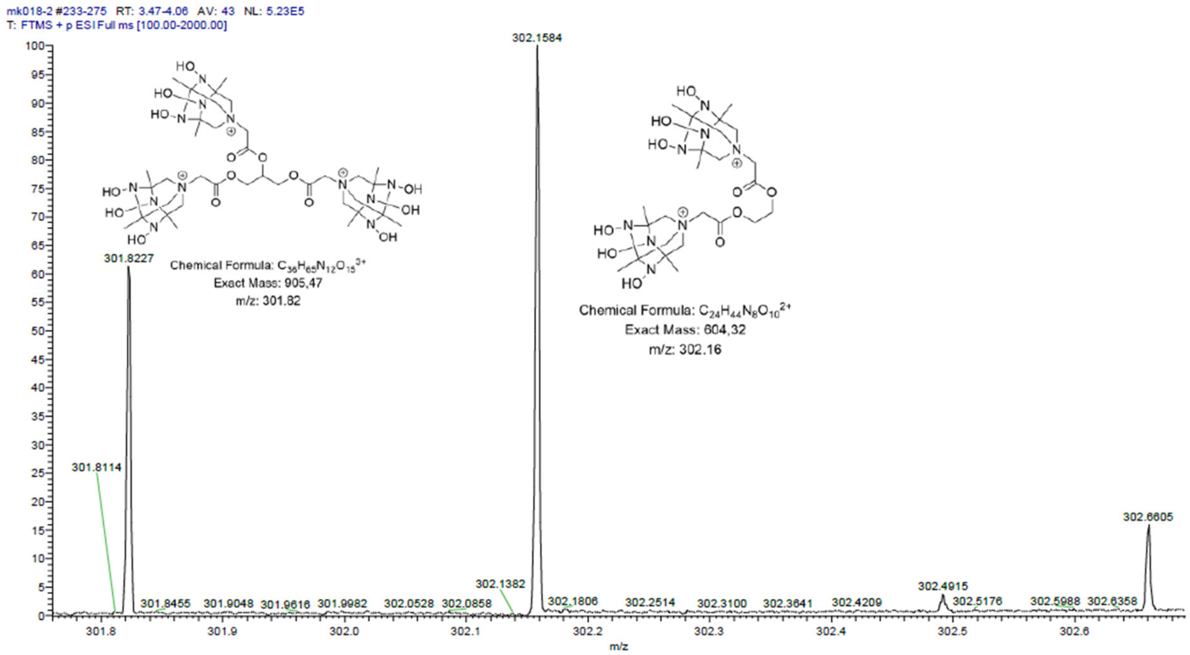

Supplementary Figure S34. mass spectrum of tris(TAAD acetoxy)propane

5 Exchange equilibrium data

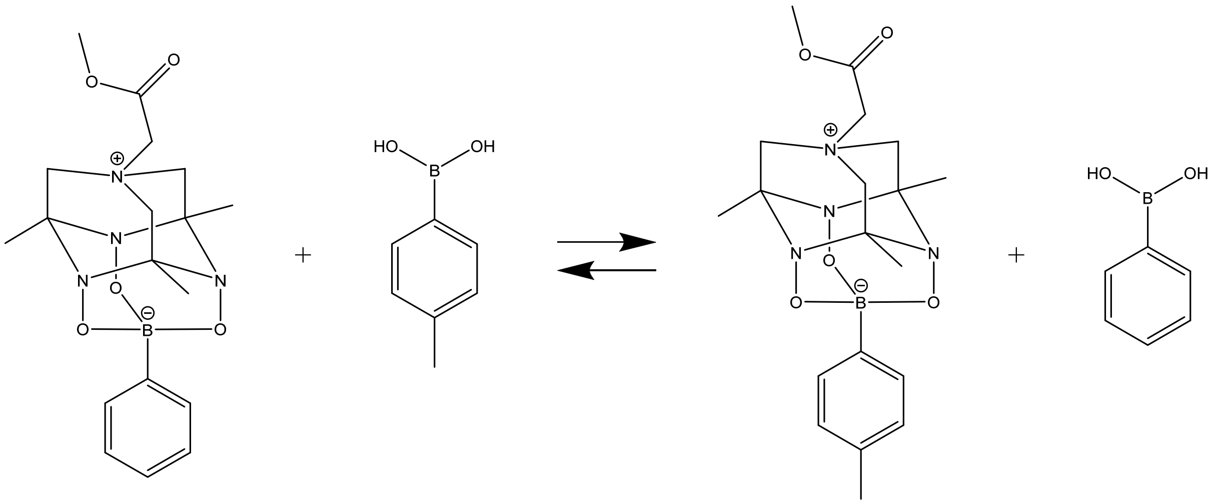

Supplementary Scheme S8. Equilibrium reaction between N-methylacetate-O-phenylboronate-TAAD and p-tolylboronic acid.

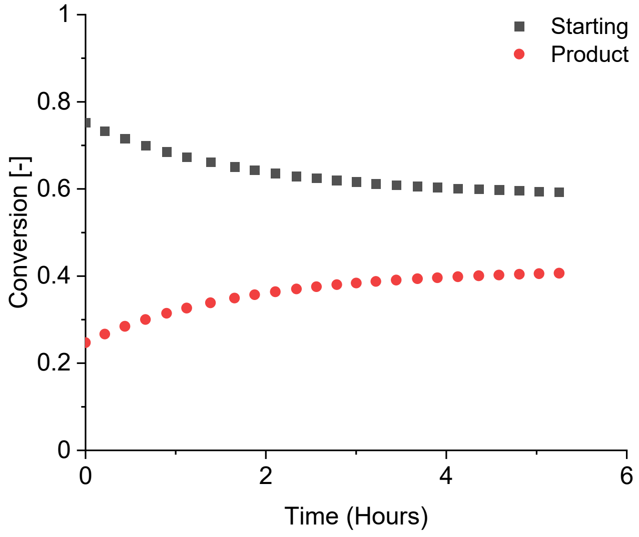

Supplementary Figure S35. Exchange reaction between N-methylacetate-O-phenylboronate-TAAD and p-tolylboronic acid after a 3 day stability test, as monitored by ^{1}H NMR.

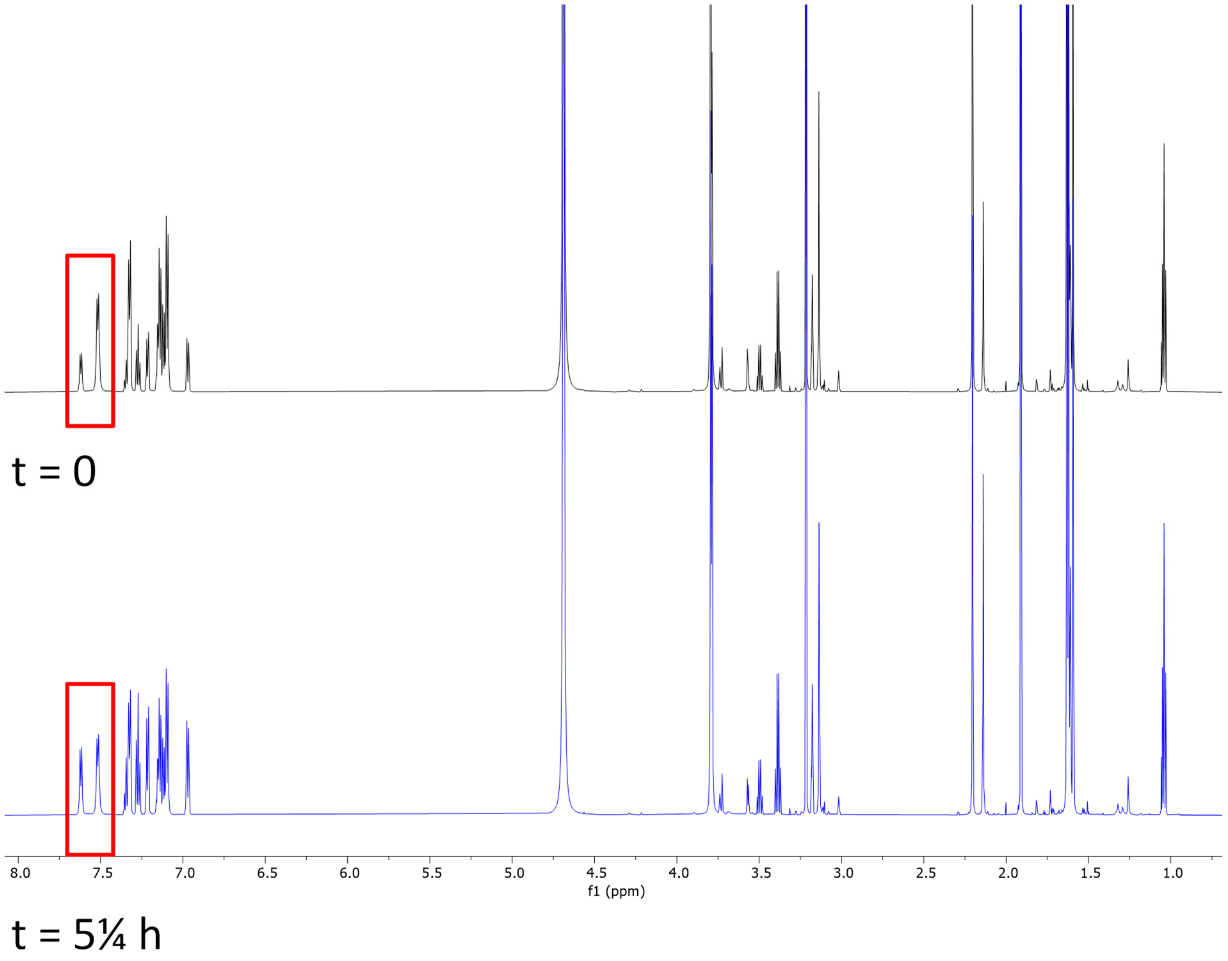

Supplementary Figure S36. Representative ^{1}H-NMR data of the exchange reaction between N-methylacetate-O-phenylboronate-TAAD and p-tolylboronic acid after a 3 day stability test. The peak at 7.72 ppm belongs to the product, while the peak at 7.62 ppm belongs to the starting complex. In both cases the signal corresponds to the aromatic protons *ortho* to the boronic acid. By integration of the peaks the progress of the exchange reaction could be monitored as function of time (see Supplementary Figure S35).

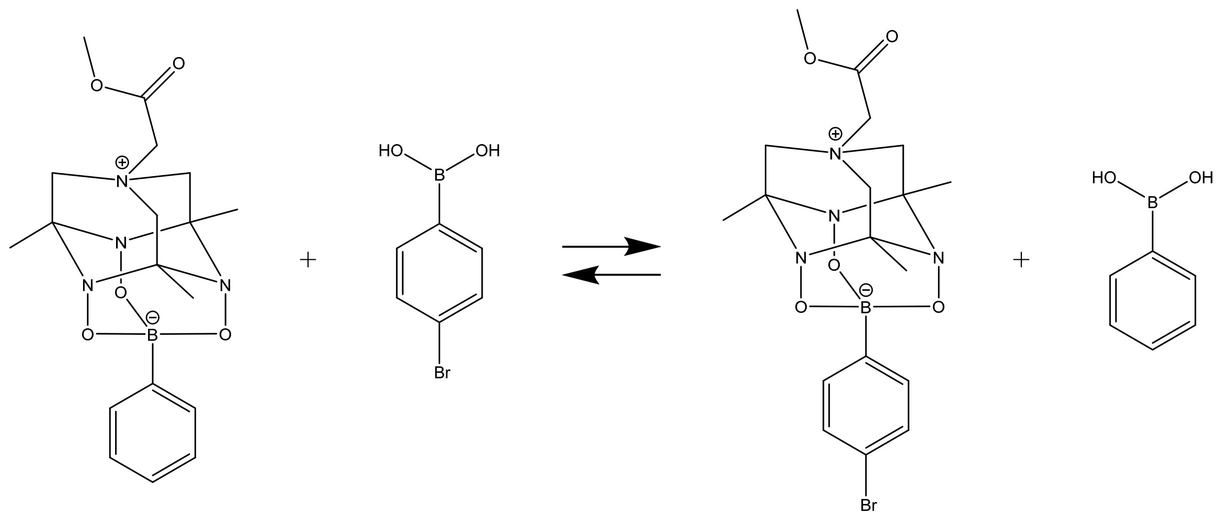

Supplementary Scheme S9. Equilibrium reaction between N-methylacetate-O-phenylboronate-TAAD and 4-bromophenylboronic acid.

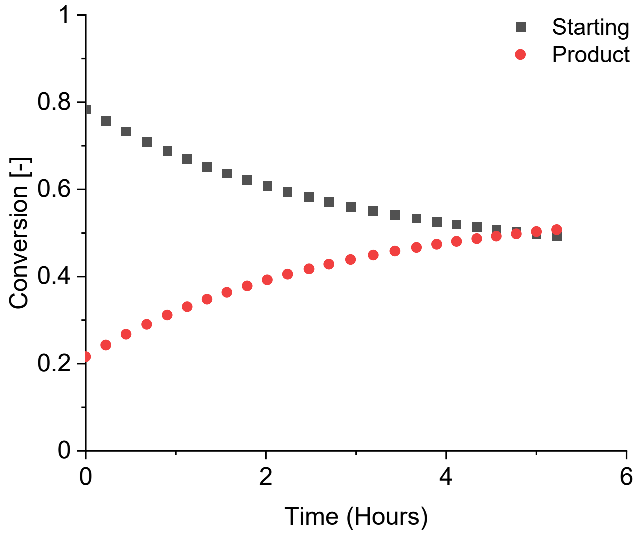

Supplementary Figure S37. Exchange reaction between N-methylacetate-O-phenylboronate-TAAD and 4-bromophenylboronic acid after a 3 day stability test, as monitored by ^{1}H NMR.

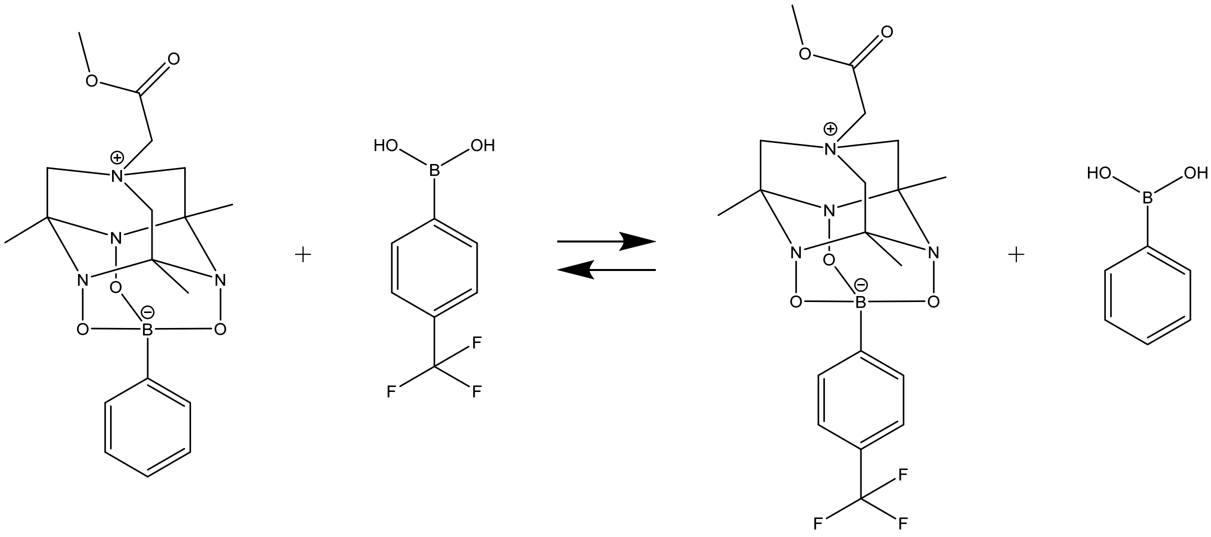

Supplementary Scheme S10. Equilibrium reaction between N-methylacetate-O-phenylboronate-TAAD and 4-(trifluoromethyl)phenylboronic acid.

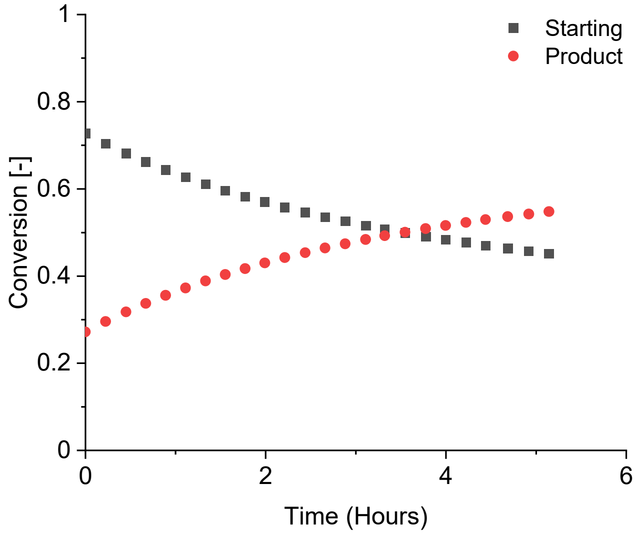

Supplementary Figure S38. Exchange reaction between N-methylacetate-O-phenylboronate-TAAD and 4-(trifluoromethyl)phenylboronic acid after a 3 day stability test, as monitored by ^{1}H NMR.

6 Rheology data

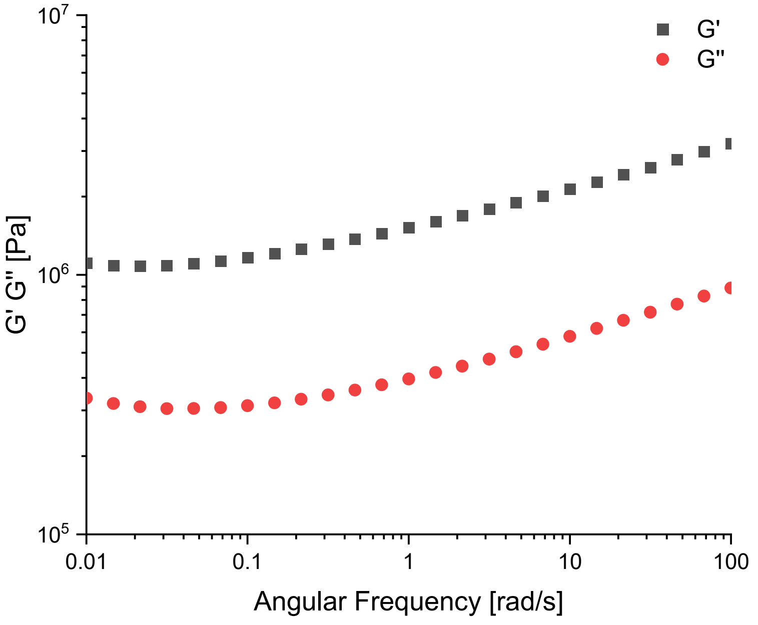

Supplementary Figure S39. Frequency sweep (0.5% strain; 25 °C) of a 20% crosslinked boronate-TAAD network.

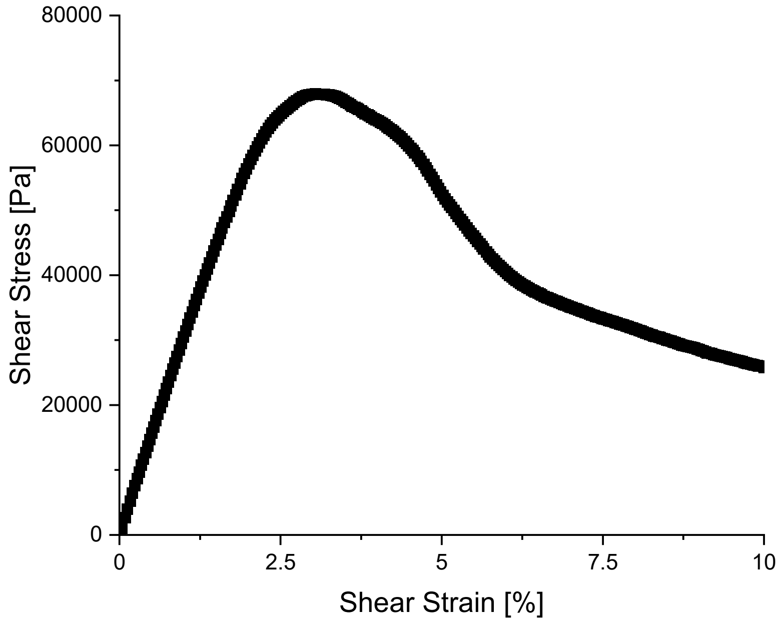

Supplementary Figure S40. Stress strain curve (25 °C) of a a 20% crosslinked boronate-TAAD network.

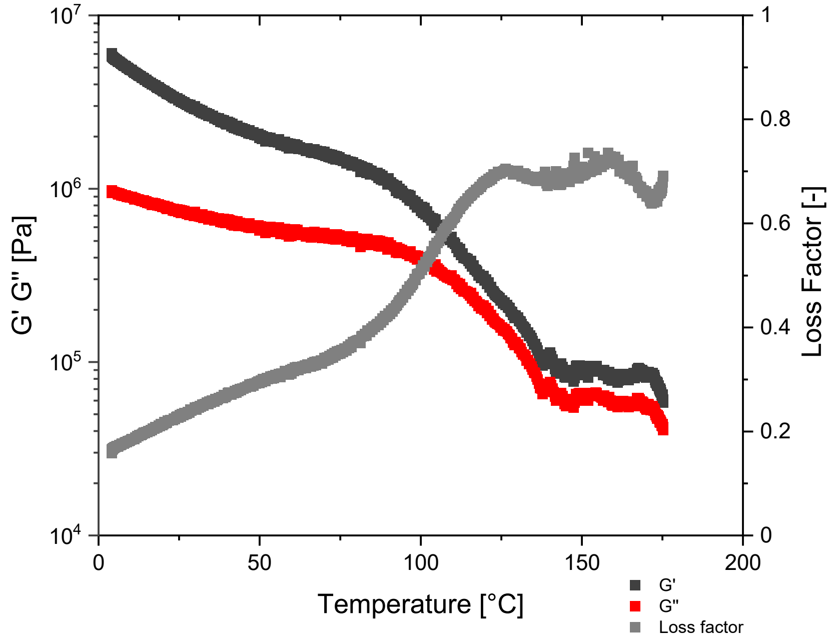

Supplementary Figure S41. Temperature sweep (0.5% strain) of a a 20% crosslinked boronate-TAAD network.

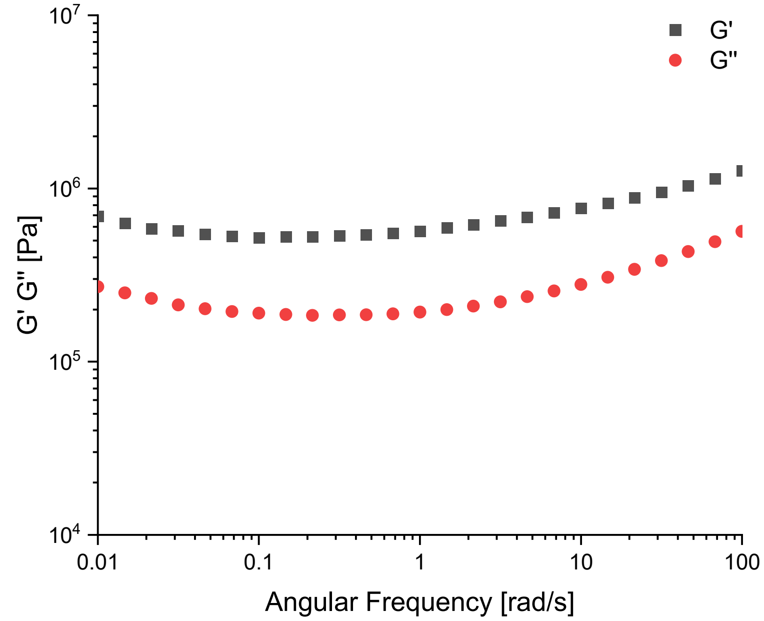

Supplementary Figure S42. Frequency sweep (0.5% strain; 25 °C) of a 33% crosslinked boronate-TAAD network.

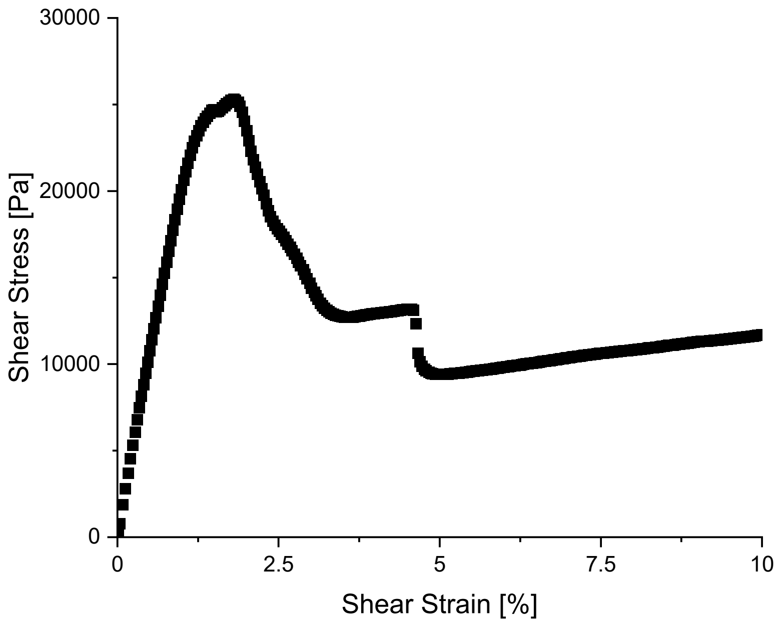

Supplementary Figure S43. Stress strain curve (25 °C) of a 33% crosslinked boronate-TAAD network.

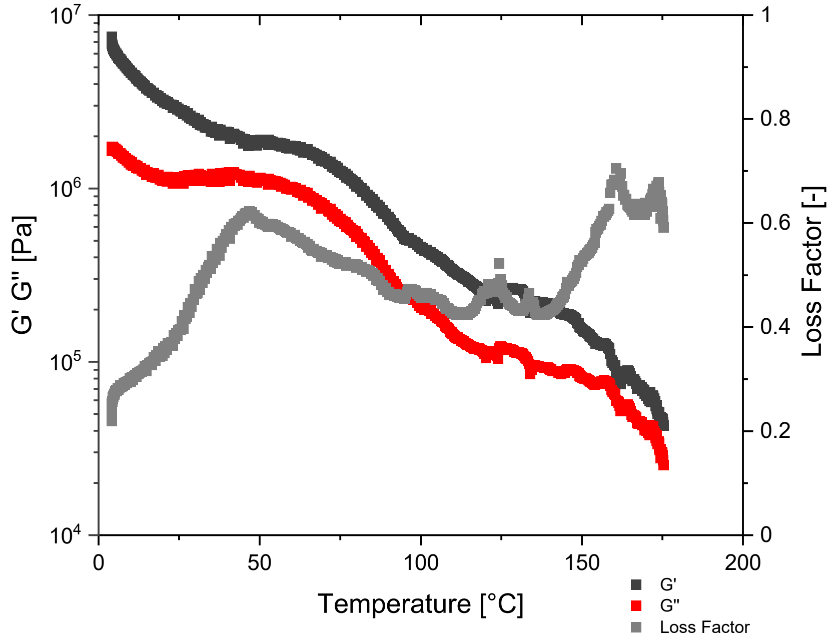

Supplementary Figure S44. Temperature sweep (0.5% strain) of a 33% crosslinked boronate-TAAD network.

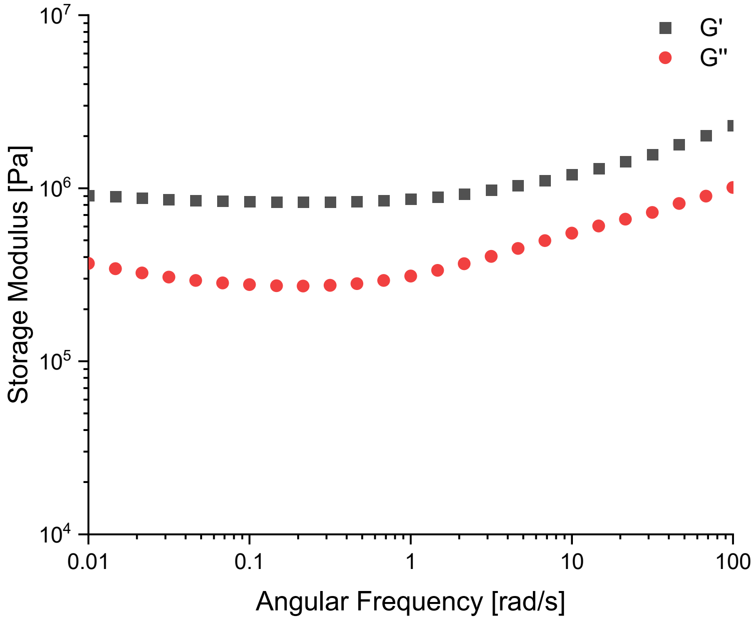

Supplementary Figure S45. Frequency sweep (0.5% strain; 25 °C) of a 33% crosslinked boronate-TAAD network with 0.25 wt% PTSA.

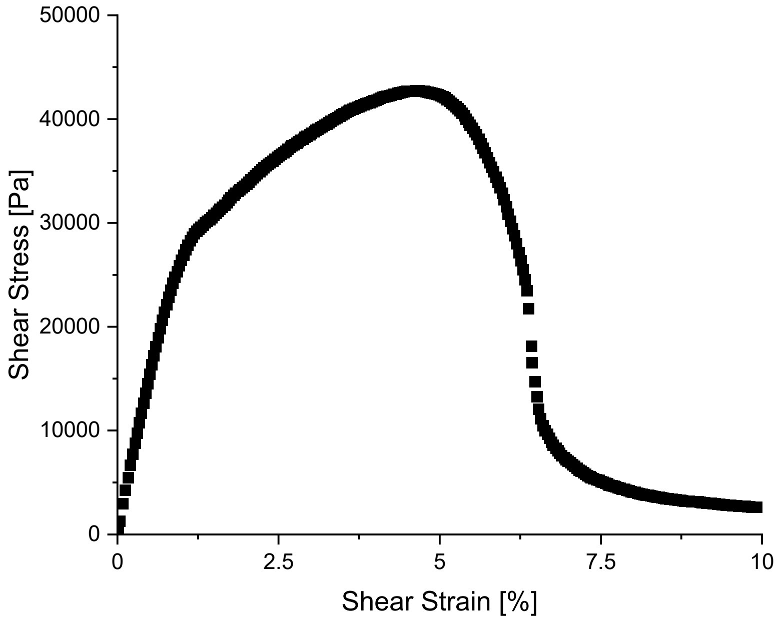

Supplementary Figure S46. Stress strain curve (25 °C) of a 33% crosslinked boronate-TAAD network with 0.25 wt% PTSA.

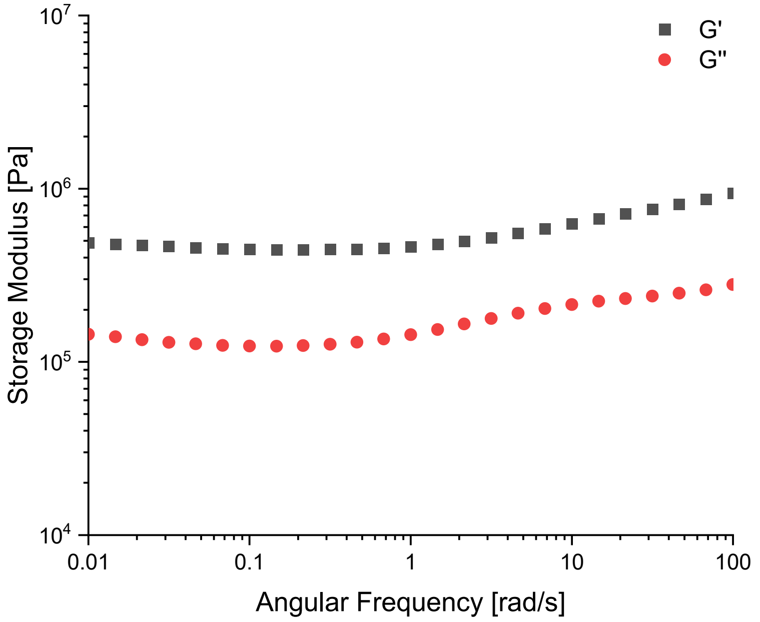

Supplementary Figure S47. Frequency sweep (0.5% strain; 25 °C) of a 33% crosslinked boronate-TAAD network with 0.5 wt% PTSA.

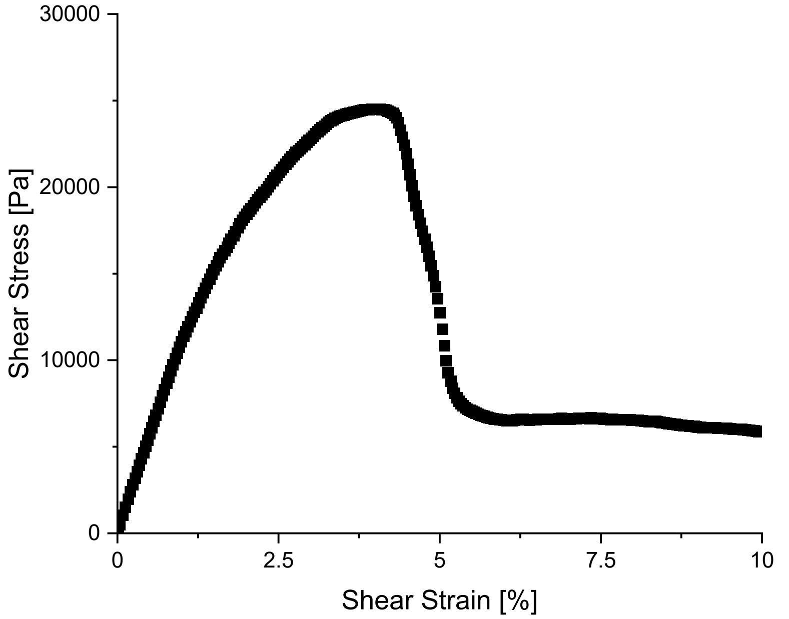

Supplementary Figure S48. Stress strain curve (25 °C) of a 33% crosslinked boronate-TAAD network with 0.5 wt% PTSA.

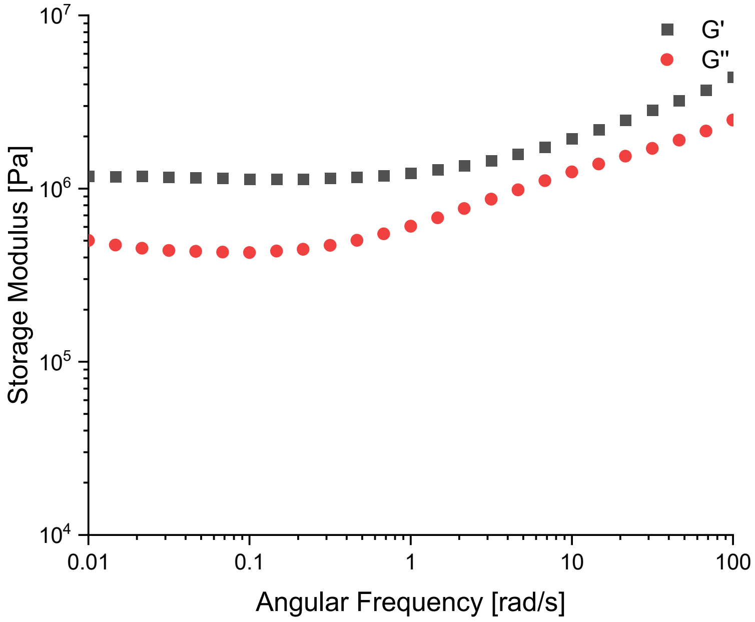

Supplementary Figure S49. Frequency sweep (0.5% strain; 25 °C) of a 33% crosslinked boronate-TAAD network with 1 wt% PTSA.

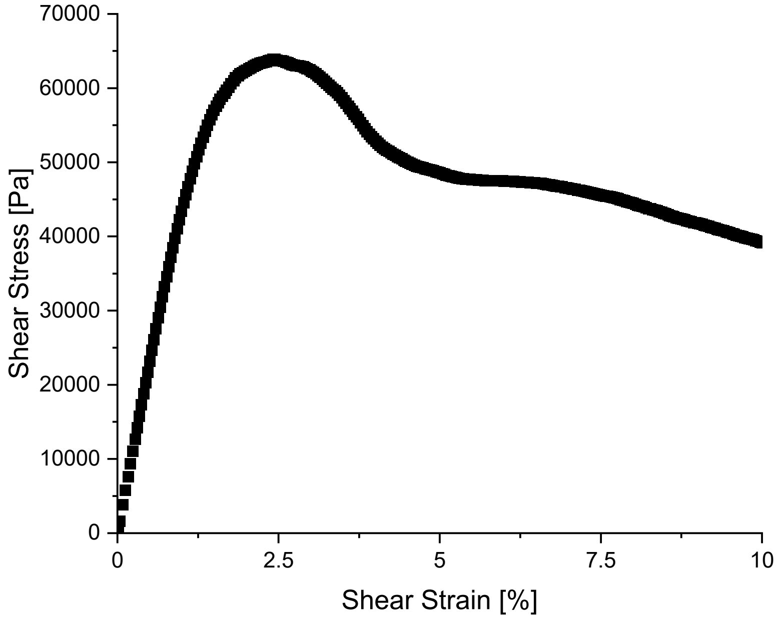

Supplementary Figure S50. Stress strain curve (25 °C) of a 33% crosslinked boronate-TAAD network with 1 wt% PTSA.

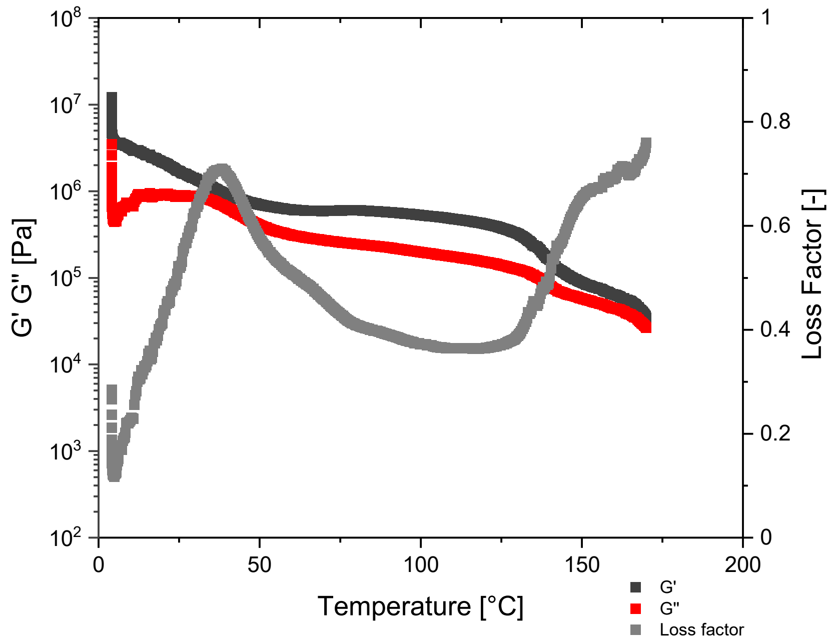

Supplementary Figure S51. Temperature sweep (0.5% strain) of a 33% crosslinked boronate-TAAD network with 1 wt% PTSA.

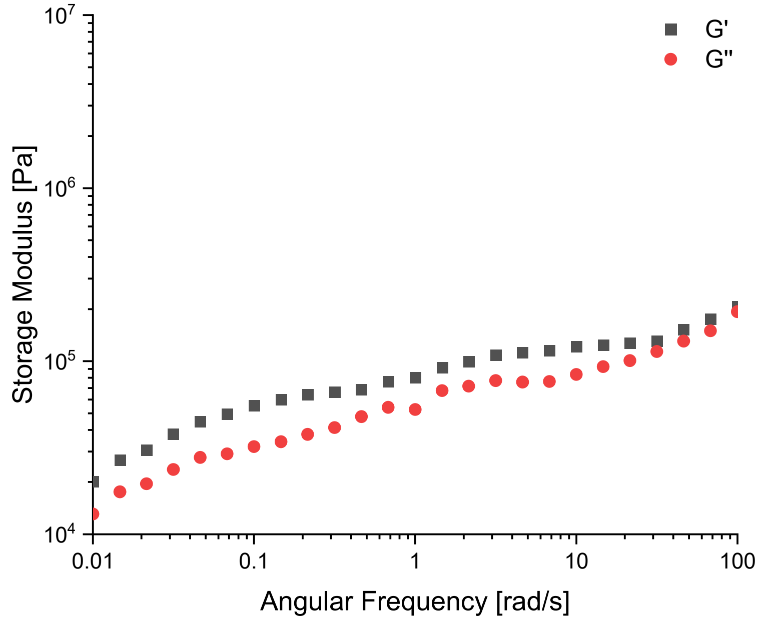

Supplementary Figure S52. Frequency sweep (0.5% strain; 25 °C) of a 33% crosslinked boronate-TAAD network with 2.5 wt% PTSA.

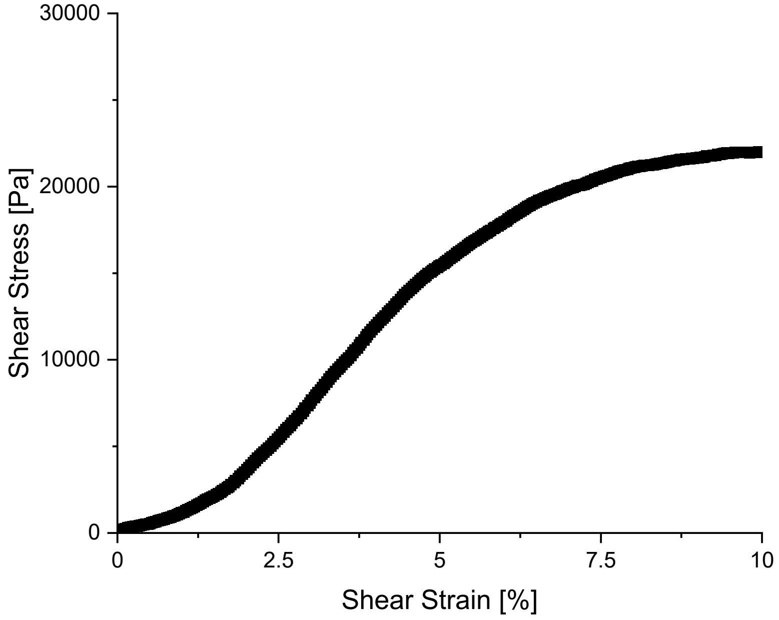

Supplementary Figure S53. Stress strain curve (25 °C) of a 33% crosslinked boronate-TAAD network with 2.5 wt% PTSA.

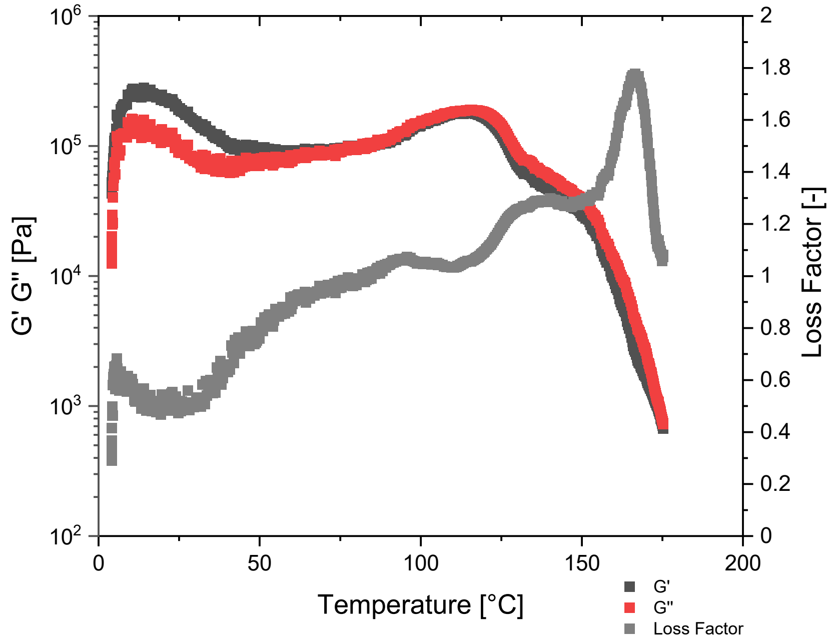

Supplementary Figure S54. Temperature sweep (0.5% strain) of a 33% crosslinked boronate-TAAD network with 2.5 wt% PTSA.

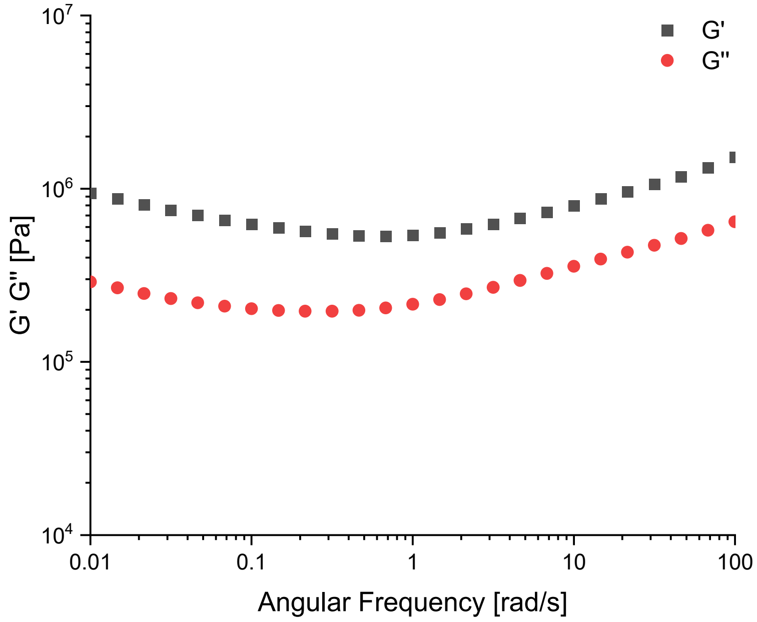

Supplementary Figure S55. Frequency sweep (0.5% strain; 25 °C) of a 33% crosslinked boronate-TAAD network with 5 wt% PTSA.

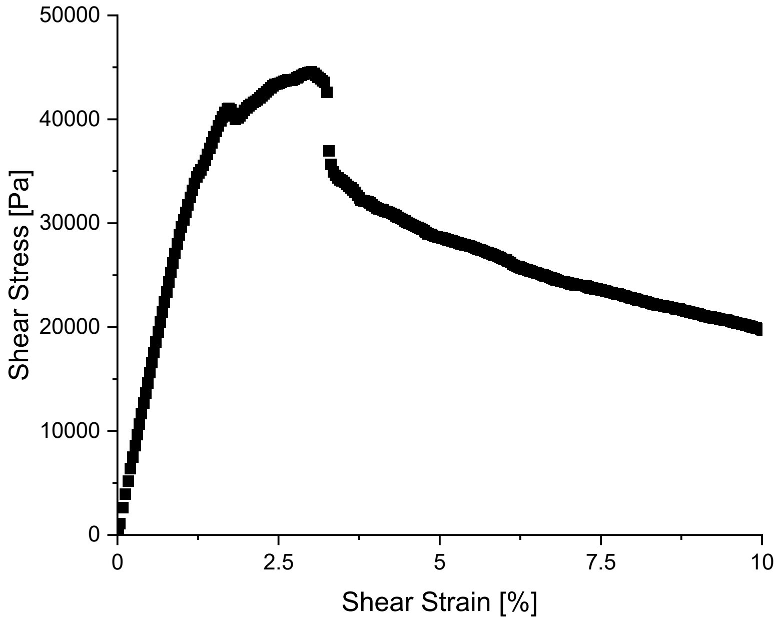

Supplementary Figure S56. Stress strain curve (25 °C) of a 33% crosslinked boronate-TAAD network with 5 wt% PTSA.

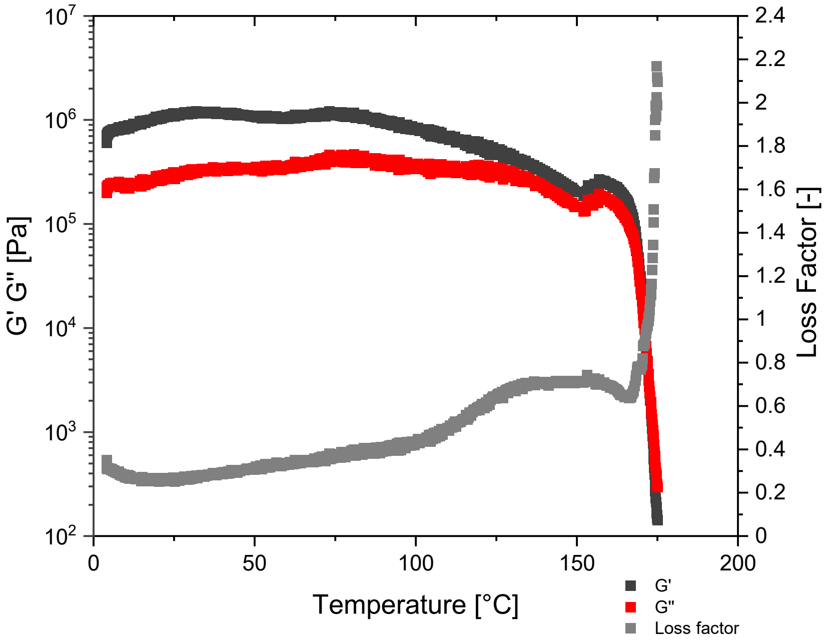

Supplementary Figure S57. Temperature sweep (0.5% strain) of a 33% crosslinked boronate-TAAD network with 5 wt% PTSA.

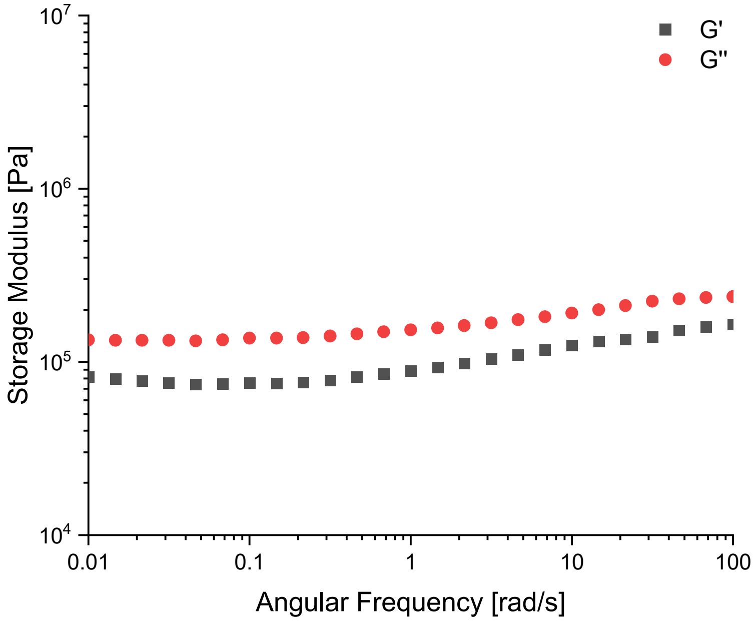

Supplementary Figure S58. Frequency sweep (0.5% strain; 25 °C) of a 33% crosslinked boronate-TAAD network with 7.5 wt% PTSA.

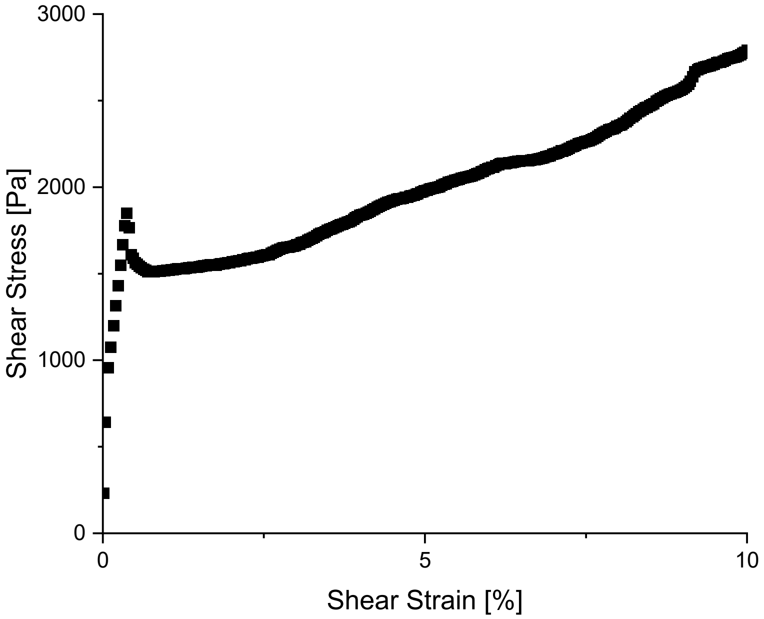

Supplementary Figure S59. Stress strain curve (25 °C) of a 33% crosslinked boronate-TAAD network with 7.5 wt% PTSA.

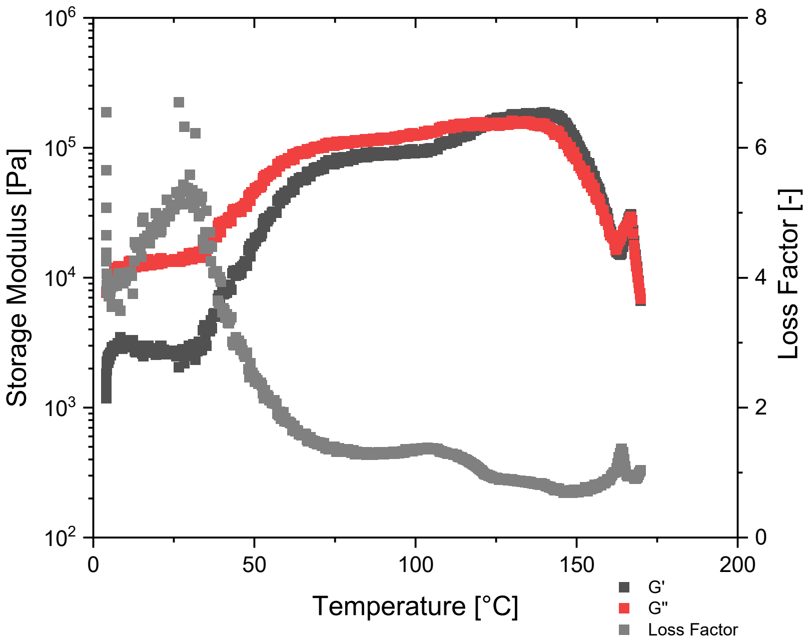

a

Supplementary Figure S60. Temperature sweep (0.5% strain; 25 °C) of a 33% crosslinked boronate-TAAD network with 7.5 wt% PTSA.

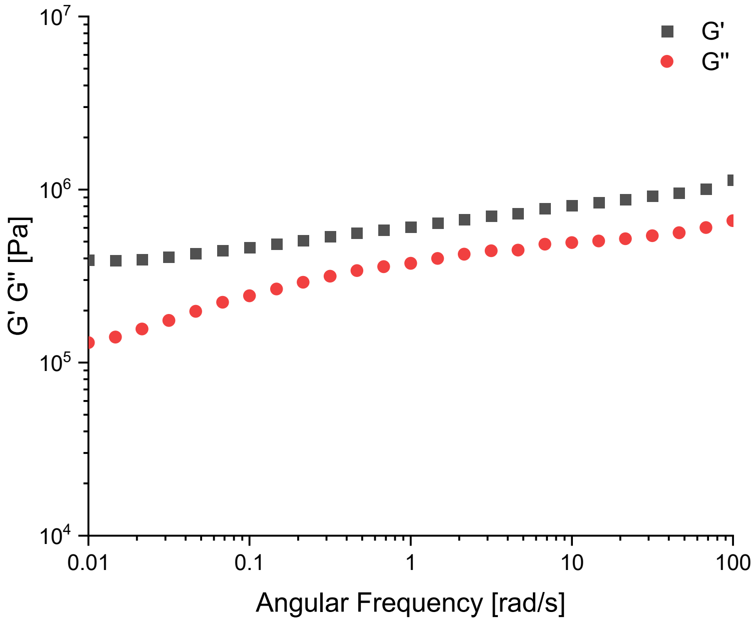

Supplementary Figure S61. Frequency sweep (0.5% strain; 25 °C) of a 33% crosslinked boronate-TAAD network with 10 wt% PTSA.

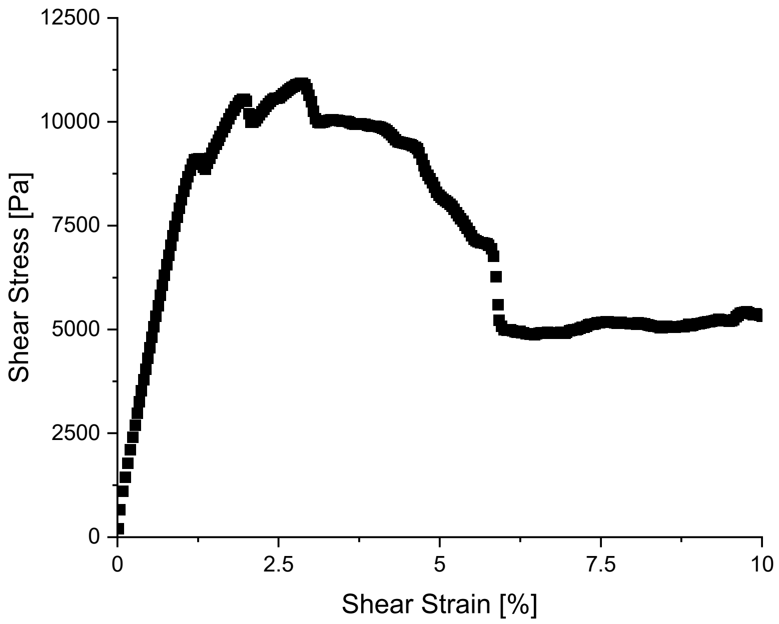

Supplementary Figure S62. Stress strain curve (25 °C) of a 33% crosslinked boronate-TAAD network with 10 wt% PTSA.

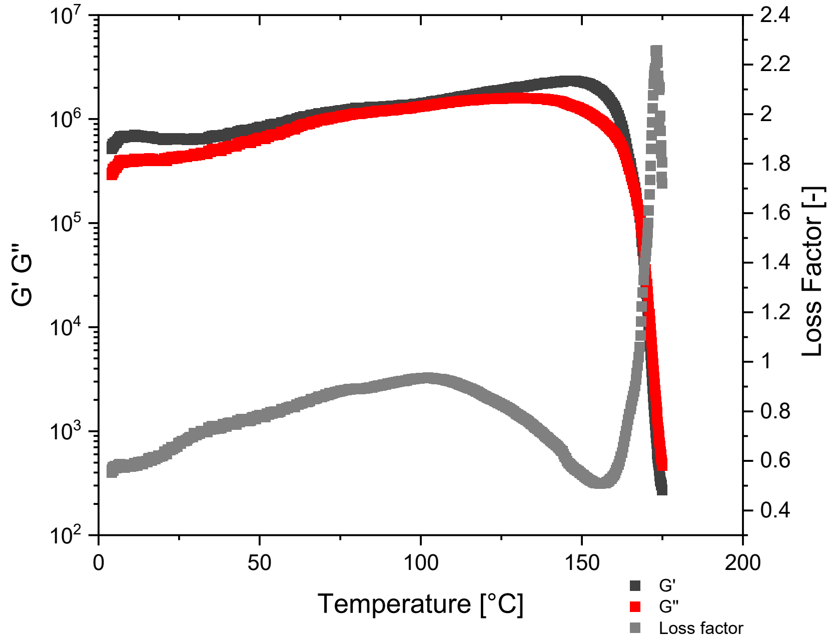

Supplementary Figure S63. Temperature sweep (0.5% strain) of a 33% crosslinked boronate-TAAD network with 10 wt% PTSA.

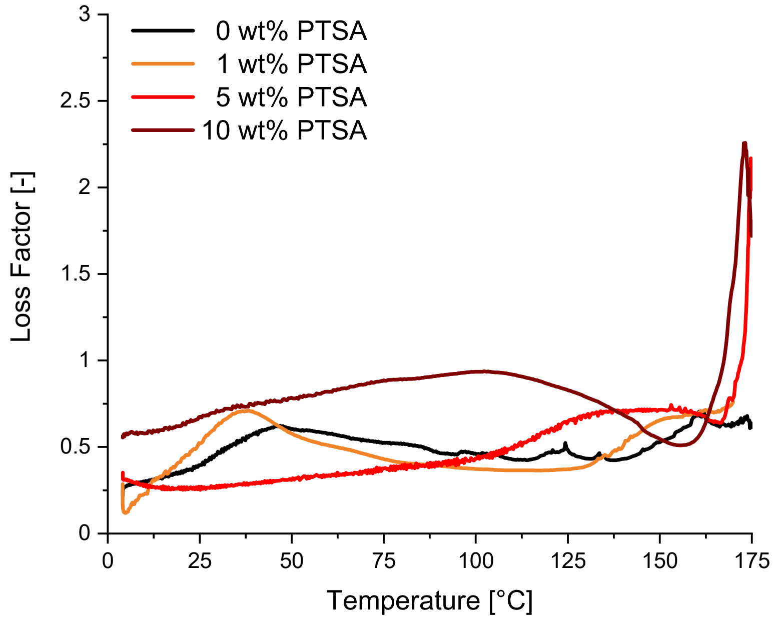

Supplementary Figure S64. Loss factor as function of temperature for a series of 33% crosslinked boronate-TAAD networks with varying wt% of PTSA.

7 TGA data

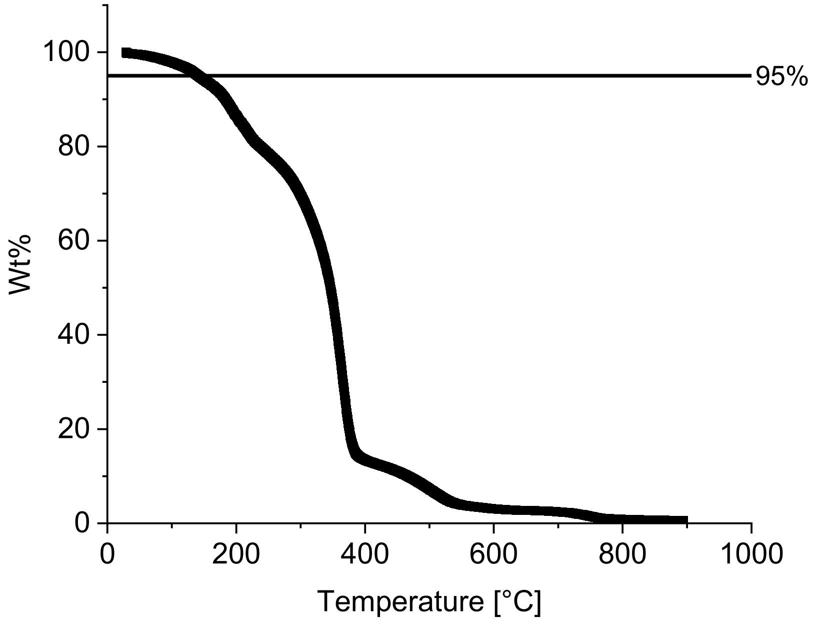

Supplementary Figure S65. TGA data of a 10% crosslinked material.

8 References

Bao, C.Y., Jiang, Y.J., Zhang, H.Y., Lu, X.Y., and Sun, J.Q. (2018). Room-Temperature Self-Healing and Recyclable Tough Polymer Composites Using Nitrogen-Coordinated Boroxines. *Adv. Funct. Mater.* 28(23), 1800560. doi: 10.1002/adfm.201800560.

Golovanov, I.S., Mazeina, G.S., Nelyubina, Y.V., Novikov, R.A., Mazur, A.S., Britvin, S.N., et al. (2018). Exploiting Coupling of Boronic Acids with Triols for a pH-Dependent "Click-Declick" Chemistry. *J. Org. Chem.* 83(17), 9756-9773. doi: 10.1021/acs.joc.8b01296.
